# Oxidative stress-induced circKIF18A downregulation impairs MCM7-mediated anti-senescence in intervertebral disc degeneration

**DOI:** 10.1038/s12276-022-00732-0

**Published:** 2022-03-24

**Authors:** Jianle Wang, Dongdong Xia, Yan Lin, Wenbin Xu, Yaosen Wu, Jiaoxiang Chen, Junjie Chu, Panyang Shen, Sheji Weng, Xiangyang Wang, Lifeng Shen, Shunwu Fan, Shuying Shen

**Affiliations:** 1 Department of Orthopedics, Sir Run Run Shaw Hospital, Zhejiang University School of Medicine, 310020 Hangzhou, Zhejiang China; 2 Key Laboratory of Musculoskeletal System Degeneration and Regeneration Translational Research of Zhejiang Province, 310020 Hangzhou, Zhejiang China; 3 Department of Orthopedics, Ningbo First Hospital, 315010 Ningbo, Zhejiang China; 4 Department of Orthopaedics, The Second Affiliated Hospital and Yuying Children’s Hospital of Wenzhou Medical University, 325088 Wenzhou, Zhejiang Province China; 5 Key Laboratory of Orthopaedics of Zhejiang Province, 325088 Wenzhou, Zhejiang Province China; 6 Department of Head and Neck Surgery, Institute of Micro-Invasive Surgery of Zhejiang University, Sir Run Run Shaw Hospital, Zhejiang University School of Medicine, 310020 Hangzhou, Zhejiang China

**Keywords:** Diseases, Ageing

## Abstract

Low back pain, triggered by intervertebral disc degeneration (IVDD), is one of the most common causes of disability and financial expenditure worldwide. However, except for surgical interventions, effective medical treatment to prevent the progression of IVDD is lacking. This study aimed to investigate the effects of circKIF18A, a novel circRNA, on IVDD progression and to explore its underlying mechanism in IVDD. In this study, we found that oxidative stress was positively correlated with nucleus pulposus cell (NPC) senescence in IVDD and that circKIF18A was downregulated in IVDD and attenuated senescent phenotypes such as cell cycle arrest and extracellular matrix degradation in NPCs. Mechanistically, circKIF18A competitively suppressed ubiquitin-mediated proteasomal degradation of MCM7, and the protective effects of circKIF18A on NPCs were partially mediated by MCM7 under oxidative stress. Intradiscal injection of adenoviral circKIF18A ameliorated IVDD in a rat model. This study revealed that circKIF18A regulates NPC degeneration by stabilizing MCM7 and identified a novel signaling pathway, the circKIF18A-MCM7 axis, for anti-senescence molecular therapy in IVDD.

## Spinal disease: Circular RNA protects against disk degeneration

A non-coding circular RNA molecule that prevents spinal cells from undergoing premature ageing offers a new therapeutic target for treating intervertebral disc degeneration (IVDD), a major cause of lower back pain. Shuying Shen of Zhejiang University School of Medicine, China, and colleagues took samples from the soft, gelatinous central portion of the intervertebral disk, the so-called nucleus pulposus, and looked for circular RNAs with high expression levels in healthy individuals and low levels in people with IVDD. They identified a specific RNA in this way, and showed how this regulatory molecule promotes the activity of a protein involved in enhancing the proliferative capacity of nucleus pulposus tissues. In rats, injections of a gene therapy vector encoding this RNA helped ameliorate signs of IVDD, highlighting the potential for similar therapeutic strategies in people with IVDD.

## Introduction

Low back pain, triggered by intervertebral disc degeneration (IVDD), is one of the most common causes of disability and financial expenditure worldwide^1^. Surgical interventions, including percutaneous endoscopic lumbar discectomy, intervertebral disc fusion, and fixation, are often applied clinically for severe IVDD but are accompanied by many complications, such as recurrence of neurological symptoms and degeneration of adjacent intervertebral discs (IVDs)^2,3^. Hence, it is essential to explore the mechanism of IVDD and improve the strategy for molecular targeted treatment. As the largest avascular tissue in the body, IVDs consist of the annulus fibrosus (AF), cartilage endplate, and nucleus pulposus (NP). It has been demonstrated that nucleus pulposus cells (NPCs), which are surrounded by extracellular matrix (ECM), play a critical role in regulating IVDD^4^. To date, different theories, including apoptosis, autophagy, and inflammation, have been proposed to determine the mechanism of IVDD^5,6^. More importantly, senescence contributes to cell cycle arrest and ECM degradation in NPCs^7,8^, suggesting that anti-senescence is also a key point in attenuating the progression of IVDD.

With advances in bioinformatics and RNA high-throughput sequencing technology in recent years, circular RNAs (circRNAs), single-stranded covalently closed-loop structures, have attracted attention. The biogenesis of circRNAs is robust, conserved, and tissue- or time-specific, indicating that these molecules might have a significant role in biological processes and cell fate^9,10^. Emerging evidence demonstrates that circRNAs are associated with various diseases, such as cancer and degenerative diseases^11,12^. Because of their circular structure, the identification and quantification of circRNAs, as well as the characterization of their biological functions, is challenging, resulting in limited knowledge of circRNAs in diseases.

Senescence, characterized by a state of proliferation arrest with different degenerative phenotypes, has been suggested to be correlated with chronic diseases^13^. It has been reported that accumulation of senescent NPCs is observed in degenerative IVDs, implying that senescence might play an important role in the process of IVDD^14^. However, the underlying mechanism of NPC senescence is exceptionally complicated. Thus, identifying critical senescence-related molecular targets in NPCs appears to be important in delaying the progression of IVDD.

The minichromosome maintenance (MCM) complex, composed of six highly conserved MCM proteins, is considered an essential regulator in the step of prereplication complex formation, which melts DNA at the origin and unwinds DNA at replication forks^15,16^. MCM7 is correlated with tumorigenesis and has been found to be a potential human malignant tumor biomarker^17^. Recent studies have demonstrated that MCM7 knockdown promotes senescent phenotypes (e.g., cell cycle arrest and senescence-associated morphological changes) in fibroblasts^17,18^, indicating that MCM7 might also act as a crucial mediator of NPC degeneration.

Here, we demonstrated that oxidative stress was positively correlated with NPC senescence and verified circKIF18A (hsa_circ_0021535) in IVDD. CircKIF18A competitively inhibited UBE3A-mediated proteasomal degradation of MCM7 and subsequently suppressed NPC senescence via the p53-p21 and p16 signaling pathways. In addition, circKIF18A protected NPCs from oxidative stress-induced senescence in a MCM7-dependent manner. This study identified a novel circRNA-RNA-binding protein (RBP) axis in NPC senescence and augmented molecular therapeutic strategies for IVDD.

## Materials and methods

### Ethics statement

All interventions, treatments, and animal care procedures were performed according to the guidelines of the Ethics Committee of Sir Run Run Shaw Hospital and the Guide for the Care and Use of Laboratory Animals published by the National Institutes of Health. The collection of human NPs and further experiments involving human NPs were approved by the Sir Run Run Shaw Hospital of Zhejiang University School of Medicine in accordance with the guidelines of the Declaration of Helsinki; all participants provided written informed consent.

### Human NPs collection

One hundred human NP tissues from patients who underwent lumbar surgery and had no history of diabetes, hypertension, or smoking were collected for subsequent experiments (Supplementary Table 5). In this study, we divided these tissues into five grades in strict accordance with the Pfirrmann grading scale^19^. Patients with Pfirrmann grade I and II disc degeneration were selected for the control group, and the patients in the IVDD group were those who would like to accept discectomy and vertebral fusion and had Pfirrmann grade III, IV, or V disc degeneration.

### Human NPC isolation and culture

Human NPs were collected and taken to the laboratory as soon as possible. After washing with PBS and cutting into pieces, NPs were digested in 0.2% type II collagenase (Sigma, USA) for 2–3 h at 37 °C. The digested tissues were washed again with PBS and cultured in Dulbecco’s modified Eagle’s medium (Thermo Fisher Scientific, USA) supplemented with 10% fetal bovine serum (Thermo Fisher Scientific) and antibiotics (1% penicillin/streptomycin/amphotericin B) in an incubator with 5% CO_2_ at 37 °C.

### ROS detection assay

A ROS assay kit (S0033S, Beyotime, Shanghai, China) was applied to measure cellular ROS levels according to the instructions. Briefly, after treatment as described above, NPCs were incubated with DCFH-DA solution (10 μM) at 37 °C for 30 min. After washing with basal culture medium three times, images of DCFH-DA staining were acquired using a fluorescence microscope (Carl Zeiss, Jena, Germany).

### IVDD model establishment and injection of virus

A total of 27 male SD rats (250–300 g) were randomly divided into three groups (namely, the control group, IVDD group, and IVDD + circKIF18A group; *n* = 9 rats per group). Following anesthetization with 2% (w/v) pentobarbital (40 mg/kg), the discs (Co7/8) were punctured and injected with 2 μL of adenovirus solution using a 33-gauge needle connected to a microliter syringe (Hamilton, Bonaduz, Switzerland). Rats in the control group underwent only a sham operation, and rats in the IVDD group were injected with empty vector adenovirus. Rats in the IVDD + circKIF18A group were injected with circKIF18A overexpression adenovirus. Two weeks later, rats were sacrificed. The discs were collected, fixed with 4% paraformaldehyde for 72 h, and subsequently decalcified in 10% EDTA. Because needle puncture induced complete NP loss in four IVDD rats, the rest of the IVDD rats (*n* = 5) were used for immunofluorescence and immunohistochemical analyses.

### Fluorescence in situ hybridization (FISH)

The circKIF18A probe was designed and purchased from GenePharma (Shanghai, China). Probe signals were detected with a FISH kit (GenePharma) according to the manufacturer’s instructions, and nuclei were stained with 4′,6-diamidino-2-phenylindole (DAPI). Finally, fluorescence images were acquired using a fluorescence microscope (Carl Zeiss, Jena, Germany).

### Immunofluorescence

Cells treated with different treatments were incubated with 0.5% Triton X-100 for 1–5 min and were then washed with PBS. The cells were then blocked with 10% goat serum for 15 min at 37 °C and incubated with primary antibodies against MCM7 (A11325, Abclonal, Wuhan, China), p21 (27296-1-AP, Proteintech, Wuhan, China), and p16 (A0262, Abclonal) diluted in PBS overnight at 4 °C. The next day, the cells were incubated with Alexa Fluor 488-conjugated or Alexa Fluor 594-conjugated secondary antibodies (Abcam, MA, USA) and were then stained with DAPI. Finally, images were acquired using a fluorescence microscope (Carl Zeiss, Jena, Germany).

### Immunohistochemistry

Endogenous peroxidase activity was blocked with 3% hydrogen peroxide after deparaffinization and rehydration of paraffin sections. The sections were then treated with 0.4% pepsin (Meilunbio, Dalian, Liaoning, China) in 5 mM HCl at 37 °C for 30 min for antigen retrieval. Next, the sections were blocked with 10% goat serum for 30 min at 37 °C, incubated with primary antibodies against aggrecan (13880-1-AP, Proteintech), collagen II (28459-1-AP, Proteintech), MMP-3 (17873-1-AP, Proteintech), and MMP-13 (18165-1-AP, Proteintech) overnight at 4 °C, and finally incubated with an HRP-conjugated secondary antibody and components from a DAB horseradish peroxidase color development kit (Cwbiotech, Beijing, China). The rewashed sections were stained with hematoxylin. Immunohistochemical images were acquired using a light microscope (Nikon, Tokyo, Japan).

### Adenovirus construction

The vector pAdtrack-CMV (Hanbio, Shanghai, China) was cut to obtain a purified linearized vector. The full-length circKIF18A fragment was amplified using KOD-Plus-Neo (Toyoko, Osaka, Japan) according to the manufacturer’s instructions. Ligation of the linearized vector and fragments was performed using an HB In-Fusion^TM^ kit (Hanbio), and the ligated vector was then transformed into competent DH5α cells. After plasmid amplification, identification, and extraction, the extracted plasmids were transfected with packaging plasmids (pHBAd-BHG) into HEK-293T cells using LipoFiter^TM^ transfection reagent (HanBio). After 72 h of transfection, HEK-293T cells were collected and lysed, and the supernatant of the transfected HEK-293T cells was collected for virus purification using a ViraTrap^TM^ Adenovirus Purification Maxi Kit (Biomiga, Santiago, USA).

### Lentivirus construction

The full-length circKIF18A fragment was inserted into the pHBLV-CMV-T2A-PURO vector (Hanbio, Shanghai, China). Full-length circKIF18A, full-length MCM7, fragments of MCM7 (141–719, 1–526, and 1–140 + 527–719), and full-length UBE3A were inserted into the pGMLV-CMV-MCS-EF1-ZsGreen-T2A-Puro vector (Genomeditech, Shanghai, China). The sh-circKIF18A and sh-MCM7 fragments were inserted into the pHBLV-U6-RFP-T2A-Puro vector (Hanbio, Shanghai, China). Gene fragments for overexpression were ligated into the linearized vector using the HB In-Fusion assay kit (Hanbio). Gene fragments for knockdown were ligated into the linearized vector using T4 ligase (Beyotime, Shanghai, China). After amplification, identification, and extraction, the lentiviruses were stored at −20 °C.

### Transfection

Packaging and envelope plasmids, including pSPAX2 and pMD2.G, were cotransfected with the viral vectors into HEK-293T cells. After 48 h, the culture medium was filtered using a 0.45 μm filter, 2 μg/mL polybrene was added, and the viruses were subsequently used to transfect NPCs. Plasmid transfection was performed using Lipofectamine 3000 (Life Technologies). All transfections were performed according to the manufacturer’s instructions.

### RNA immunoprecipitation (RIP)

RIP assays were performed using a Magna RIP RNA-Binding Protein Immunoprecipitation Kit (Millipore, Billerica, MA, USA). NPCs or HEK-293T cells were transfected with the plasmids described above. An equal volume of a solution containing RIP lysis buffer, protease inhibitor cocktail, and an RNase inhibitor was added to approximately 1 × 10^7^ cells from each sample. The cell lysates were incubated with IgG, an anti-MCM7 antibody, or anti-Myc-coated beads at 4 °C overnight. Proteinase K buffer-treated RNAs were extracted using a RNeasy MinElute Cleanup Kit (Qiagen, Valencia, CA, USA) prior to reverse transcription (Accurate Biology, Hunan, China). The levels of full-length circKIF18A and different fragments of circKIF18A were measured using qRT–PCR.

### RNA extraction and quantitative real-time PCR analysis

Total cellular RNA was extracted from the cultured cells using TRIzol reagent (Accurate Biology, Hunan, China). For reverse transcription, total RNA was processed using an Evo M-MLV RT Kit for qRT-PCR (Accurate Biology). For qRT-PCR, cDNA was reverse transcribed from total RNA using a Premix Pro TaqHS qPCR Kit (Accurate Biology) in an ABI 7500 sequencing detection system (Applied Biosystems, Foster City, CA, USA). The relative quantities of full-length circKIF18A, circKIF18A fragments, and mRNAs were normalized to ACTB.

### Pulldown assay and mass spectrometry

Biotin-labeled oligonucleotide probes (Supplementary Table 6) targeting the junction site of circKIF18A were synthesized (GenePharma, Shanghai, China). Briefly, using a Biotin RNA Labeling Mix Kit (Roche, Indianapolis, IN, USA) and T7 RNA polymerase (Thermo Fisher Scientific, Massachusetts, USA), biotin-labeled RNA probes for circKIF18A were transcribed in vitro as previously described^20^. The RNA pulldown assay was performed at room temperature, and the precipitated proteins were detected using mass spectrometry at Shice Bio-tech Co., Ltd (Shanghai, China).

### Cell proliferation assay

Cell proliferation was evaluated using a Yefluor 488 EdU Flow Cytometry Assay Kit (40278ES60, Yeasen, Shanghai, China). First, NPCs were treated with prewarmed 1 × EdU solution for 24 h. After fixation with 4% paraformaldehyde, the cells were incubated with 2 mg/ml glycine solution and 0.5% Triton X-100 solution at room temperature. Then, the NPCs were treated with Click-iT solution for 30 min at room temperature. After staining with 1× Hoechst 33342, NPCs were observed with a fluorescence microscope (Carl Zeiss).

### Western blot analysis

NPCs were lysed with RIPA lysis buffer (Fudebio, Hangzhou, China) containing PMSF (Fudebio, Hangzhou, China) in an ice bath. After boiling with loading buffer (Fudebio), proteins from the samples were electrophoresed using SDS–PAGE gels at 80 V for 2 h and transferred to 0.22 μm polyvinylidene difluoride (PVDF) membranes (Millipore, MA, USA) at 280 mA for 120 min. The membranes were blocked with 5% skim milk powder in TBST for 2 h at room temperature and incubated with antibodies specific for β-actin (AF5001, Beyotime), MCM7 (A11325, Abclonal, Wuhan, China), p21 (27296-1-AP, Proteintech, Wuhan, China), p16 (A0262, Abclonal), aggrecan (13880-1-AP, Proteintech), collagen II (28459-1-AP, Proteintech), MMP-13 (18165-1-AP, Proteintech), and MMP-3 (17873-1-AP, Proteintech) overnight at 4 °C. The next day, the PVDF membranes were washed with TBST and incubated with an HRP-conjugated secondary antibody (Fudebio). After washing with TBST, bands were detected using a ChemiDoc Touch Imaging System (Bio–Rad, Hercules, CA, USA) with a chemiluminescence kit (Fudebio).

### Magnetic resonance imaging methods

IVD signals and structural changes were detected by magnetic resonance imaging on sagittal T2-weighted images using a 1.5-T clinical magnet (Philips, Amsterdam, Netherlands). The parameters for T2-weighted imaging were set according to the methods described in a previous study. Then, the degree of IVDD was evaluated using the Pfirrmann grading system^19^.

### Histological evaluation

After IVD tissues were embedded in paraffin, section (4 μm) was processed with a safranin O-fast green staining kit (Solarbio). For safranin O-fast green staining, histological images were acquired using light microscopy (Nikon, Tokyo, Japan), and the histological scores were determined according to previously described criteria^21^. Image assessment was conducted by two independent observers.

### SA-β-gal assay

After seeding in a six-well plate and washing three times with PBS, NPCs were sequentially fixed with 0.2% glutaraldehyde for 20 min at room temperature, washed twice with PBS, and stained with X-gal staining solution (1 mg/mL X-gal, 40 mM citric acid/sodium phosphate, 5 mM potassium ferricyanide, 5 mM potassium ferrocyanide, 150 mM NaCl, and 2 mM MgCl_2_) at pH 6.0. Images were randomly acquired with a Nikon microscope, and the percentage of SA-β-gal-positive cells was quantified for statistical analysis.

### Statistical analysis

Data are presented as the mean ± standard deviation of at least three independent experiments performed with samples from at least three different donors. Statistical analysis was performed using GraphPad Prism 9.0 (GraphPad Software, La Jolla, CA, USA). The data were analyzed using Student’s *t*-test for comparisons between two groups and using one-way analysis of variance followed by Tukey’s test for comparisons among different groups (three or more groups). Nonparametric data (MRI-based Pfirrmann grades and histological scores) were analyzed using the Kruskal–Wallis *H* test. Statistical significance was set at *P* < 0.05.

## Results

### Oxidative stress is associated with NPC senescence in IVDD

**Fig. 1:**
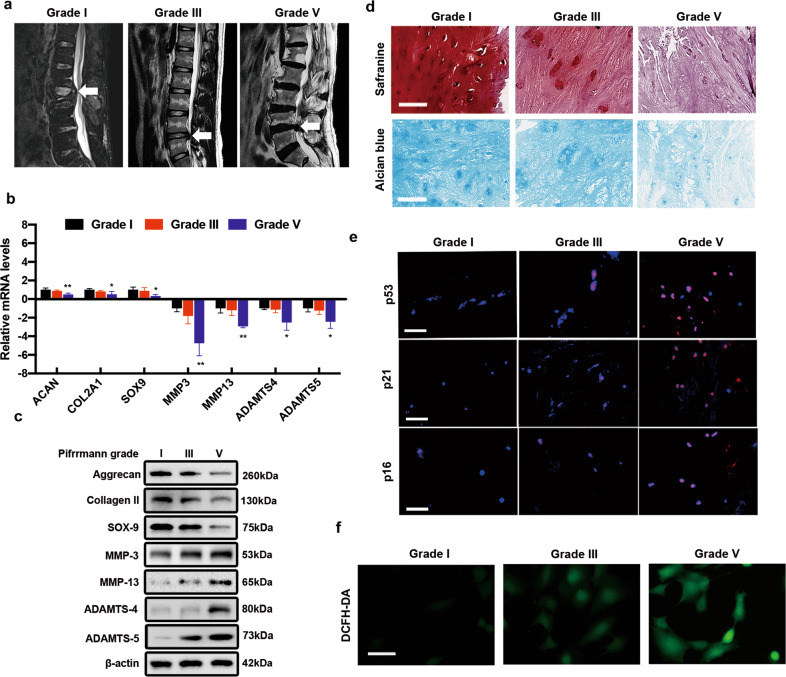
Oxidative stress is associated with NPC senescence in IVDD. **a** Representative MRIs of patients with Pfirrmann grade I, III, and V disc degeneration. White arrow: the operated segment. **b** The mRNA levels of ECM markers in NPCs extracted from different NP tissues (*n* = 3, three different donors for three independent experiments; *: versus grade I, *P* < 0.05, **P < 0.01). **c** The protein levels of ECM markers in NPCs from tissues of different Pfirrmann grades (*n* = 3, three different donors). **d** Representative images of safranin staining and alcian blue staining of different NP tissues (*n* = 5, five different donors; scale bar: 20 μm). **e** p53, p21, and p16 expression in different NP tissues was visualized by immunofluorescence staining (*n* = 5, five different donors; scale bar: 100 μm). **f** The ROS level in NPCs was evaluated using a DCFH-DA staining assay (*n* = 5, five different donors; scale bar: 25 μm). All data are presented as the mean ± SD values.

To determine whether oxidative stress is positively associated with NPC senescence in IVDD, we collected NP samples from patients with different degrees of degeneration, as described previously^6^. First, representative lumbar magnetic resonance images (MRIs) of patients with different IVDD degrees according to the Pfirrmann grading system were obtained and are presented in Fig. 1a^22^. After digesting NP tissues to obtain primary human NPCs, we performed quantitative real-time polymerase chain reaction (qRT-PCR) and western blot analyses to evaluate ECM metabolism in NPCs, which showed that the levels of catabolic markers, including MMP-13, MMP-3, ADAMTS-4, and ADAMTS-5, increased, while those of anabolic markers, such as aggrecan, collagen II, and SOX-9, decreased with aggravation of IVDD (Fig. 1b, c). In addition, safranin O staining and alcian blue staining revealed that the proteoglycan content in the grade V group was lower than that in either the grade I or grade III group (Fig. 1d). To investigate the expression of senescence markers in IVDD, we performed immunofluorescence staining and found that p53, p21, and p16 levels were increased in degenerative NPs (Fig. 1e). Emerging evidence has demonstrated that degenerative diseases are involved in oxidative stress accumulation and that in vitro oxidative stimuli induce degenerative phenotypes in NPCs^5,6,23^. However, there is no study showing the relationship between oxidative stress and senescence in IVDD. We then performed a DCFH-DA staining assay to determine the level of cellular reactive oxygen species (ROS) and found that degenerative NPCs showed higher DCFH-DA fluorescence intensities than those with lower degeneration degrees (Fig. 1f). Collectively, these results indicate that NPC senescence is accompanied by oxidative stress accumulation in IVDD.

### CircKIF18A expression is downregulated in NP tissues from IVDD patients

**Fig. 2:**
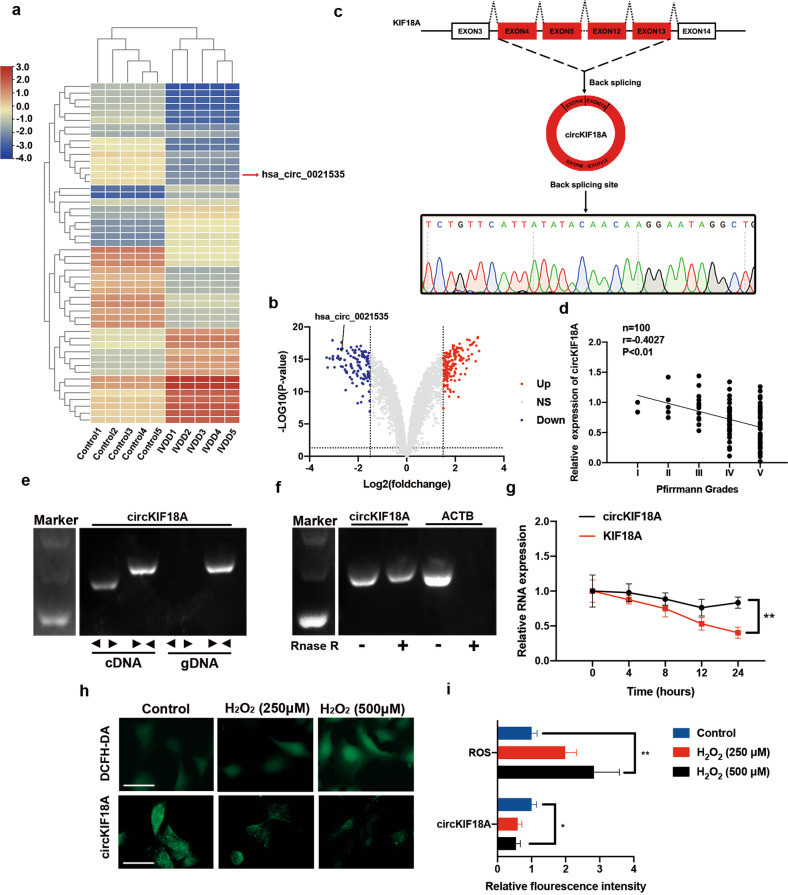
Identification of circKIF18A in IVDD. **a** Heatmap of the top 50 differentially expressed circRNAs in the GSE67566 microarray. **b** Volcano plot of 2893 circRNAs in the GSE67566 microarray. The vertical line corresponds to 1.5-fold upregulation or downregulation between the IVDD group and the control group, and the horizontal line indicates a *P* value of 0.05. **c** Schematic illustration showing the circularization of KIF18A exons 4–13 to generate circKIF18A. After RT–PCR, circKIF18A was validated using Sanger sequencing. The black arrow indicates the head-to-tail splicing site in circKIF18A. **d** The correlation between circKIF18A expression and Pfirrmann grade in patients with IVDD. **e** CircKIF18A was validated using RT-PCR. Divergent primers (< >) amplified circKIF18A, and convergent primers (> <) amplified KIF18A. **f** The effect of RNase R on the levels of circKIF18A and ACTB. **g** Time course analysis of the relative expression of circKIF18A and KIF18A in NPCs treated with actinomycin D (2 μg/mL) (*n* = 3; three different donors; *P < 0.05, **P < 0.01). **h** and **i** DCFH-DA staining and FISH assays were conducted in NPCs treated with different concentrations of H_2_O_2_ (0, 250, and 500 μM) (*n* = 5; *P < 0.05, **P < 0.01; scale bar: 50 μm). All data are presented as the mean ± SD values.

Differential expression profiles of circRNAs in NPCs were obtained from the National Center for Biotechnology Information Gene Expression Omnibus database (GSE67566)^24^. Among the 2893 circRNAs analyzed using a circRNA microarray, 113 exhibited decreased levels in degenerative NP tissues compared with control tissues in accordance with the criteria of a mean fold change of <−1.5 and a *p* value of < 0.05 (Fig. 2a, b and Supplementary Table 1). To perform a preliminary search for novel potential biomarkers for IVDD, we performed qRT–PCR to identify the top 20 circRNAs based on the absolute logFC values for the downregulated circRNAs. We found that the expression of hsa_circRNA_100772 (circBase ID: hsa_circ_0021535, also termed circKIF18A) showed the most significant reduction in the IVDD group compared to the control group (Supplementary Fig. 1a). The circKIF18A RT–PCR product was amplified by divergent primers, and the back-splice junction sequence of circKIF18A was validated using Sanger sequencing (Fig. 2c). The qRT–PCR results showed that the level of circKIF18A was negatively associated with the IVDD degree (Fig. 2d). In addition, circKIF18A was detectable in cDNA but not in gDNA of NPCs (Fig. 2e) and was more stable than its linear counterpart in NPCs treated with RNase R or actinomycin D (Fig. 2f, g). Moreover, oxidative stress is a potential indicator of NPC senescence, as shown in Fig. 1; thus, we measured the levels of circKIF18A and ROS in NPCs treated with different concentrations of H_2_O_2_. The results of fluorescence in situ hybridization (FISH) and DCFH-DA staining assays showed that circKIF18A expression was decreased and the ROS level was increased in NPCs under H_2_O_2_-induced oxidative stress (Fig. 2h, i). Therefore, circKIF18A was identified in NPCs and is negatively correlated with oxidative stress in IVDD.

### CircKIF18A regulates senescent phenotypes in NPCs

**Fig. 3:**
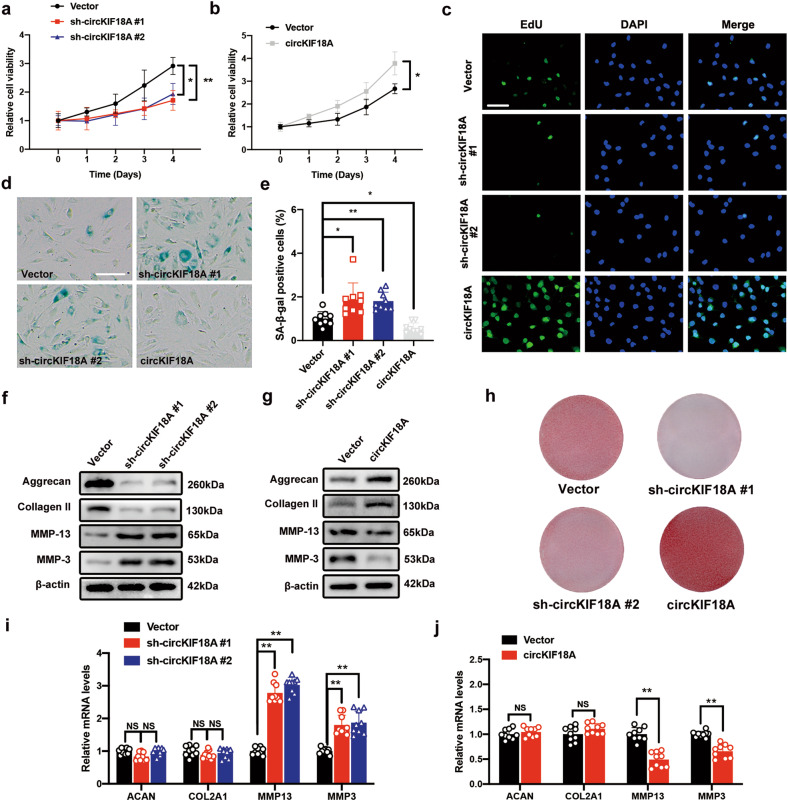
CircKIF18A regulates NPC senescent phenotypes. **a** The CCK-8 assay showed the effects of sh-circKIF18A on NPC viability (*n* = 3; three different donors; *P < 0.05, **P < 0.01). **b** The CCK-8 assay showed the impact of circKIF18A overexpression on NPC viability (*n* = 3; three different donors; *P < 0.05, **P < 0.01). **c** CircKIF18A functions in cell proliferation, as detected using an EdU assay (*n* = 5, five different donors; scale bar: 50 μm). **d** and **e** CircKIF18A overexpression reduced the number of SA-β-gal-positive cells, but circKIF18A knockdown enhanced senescence in NPCs (n = 3, three different donors for three individual experiments; *P < 0.05, **P < 0.01; scale bar: 50 μm). **f** and **g** The protein levels of aggrecan, collagen II, MMP-13, and MMP-3 in NPCs with or without circKIF18A knockdown and circKIF18A overexpression were determined using western blotting (*n* = 3, three different donors). **h** Representative safranin O staining images of NPCs transfected with sh-circKIF18A or circKIF18A overexpression lentiviruses (*n* = 3, three different donors). **i** and **j** The qRT–PCR assay showing the mRNA expression of *ACAN*, *COL2A1*, *MMP13*, and *MMP3* in circKIF18A-silenced and circKIF18A-overexpressing NPCs (*n* = 3, three different donors for three individual experiments; NS, no significance; *P < 0.05; **P < 0.01). All data are presented as the mean ± SD values.

To explore the role of circKIF18A in NPCs, we performed a CCK-8 assay to examine the viability of NPCs transfected with circKIF18A knockdown or overexpression lentivirus. As shown in Fig. 3a, b, circKIF18A knockdown reduced NPC viability, whereas the viability of circKIF18A-overexpressing NPCs increased. The results of the EdU assay revealed that circKIF18A overexpression enhanced NPC proliferation but circKIF18A knockdown had the opposite effect (Fig. 3c). Previous studies have demonstrated that senescence contributes to cell viability reduction and proliferation inhibition^5,6^. Next, we performed a β-galactosidase staining assay and found that circKIF18A overexpression decreased the number of SA-β-gal-positive cells, whereas circKIF18A knockdown enhanced NPC senescence (Fig. 3d, e). Homeostasis of ECM metabolism maintains IVD hydrostatic pressure and further supports spinal biomechanical force^5,25^. To explore the mechanism of circKIF18A in ECM metabolism, we performed western blotting and safranin O staining and found that the levels of ECM anabolic markers (aggrecan and collagen II) increased and that those of ECM catabolic markers (MMP-3 and MMP-13) decreased in circKIF18A-overexpressing NPCs; in contrast, circKIF18A silencing accelerated ECM degradation (Fig. 3f–h). Interestingly, our qRT-PCR results revealed that the mRNA expression of *MMP13* and *MMP3* was negatively regulated by circKIF18A, although the expression of *ACAN* and *COL2A1* was not affected, indicating that circKIF18A probably increased aggrecan and collagen II protein levels by suppressing catabolic enzyme (MMP-13 and MMP3)-mediated ECM degradation (Fig. 3i, j). Together, these results indicate that circKIF18A suppresses senescence in NPCs by promoting cell proliferation and balancing ECM metabolism.

### CircKIF18A increases the MCM7 level by inhibiting its ubiquitination in NPCs

**Fig. 4:**
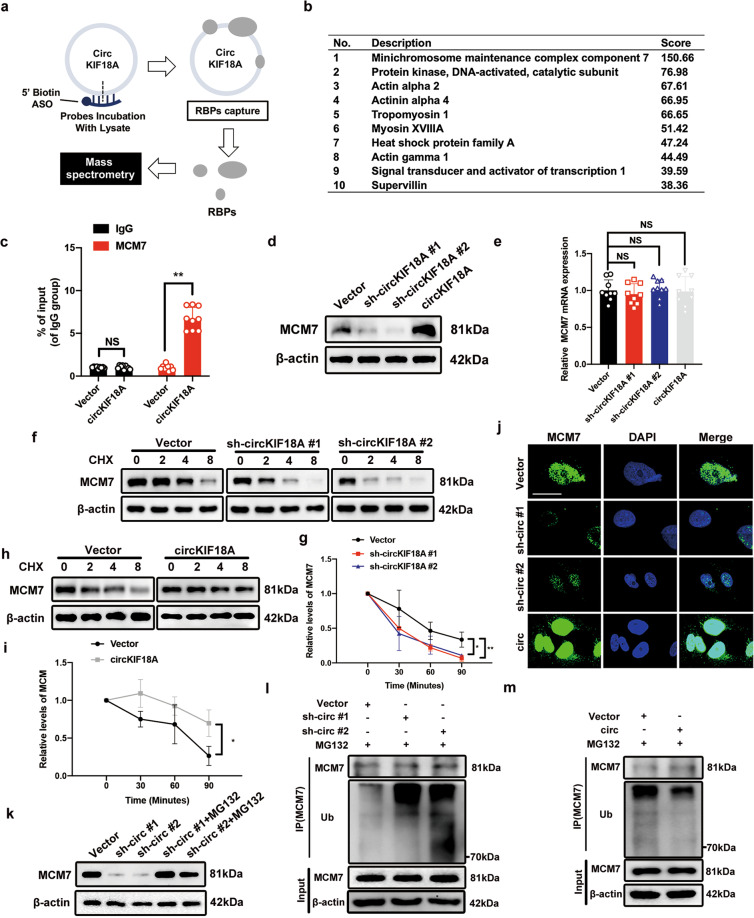
The MCM7 protein level is increased by circKIF18A in NPCs. **a** Schematic illustration of the process for identifying RBPs binding to circKIF18A. **b** The ranking list of the top ten proteins captured by biotinylated antisense oligonucleotides (ASOs) complementary to circKIF18A. **c** The interaction of circKIF18A with MCM7 was verified using RIP with an anti-MCM7 antibody and IgG in NPCs (*n* = 3, three different donors for three individual experiments; NS, no significance; *P < 0.05; **P < 0.01). **d** CircKIF18A regulated the protein level of MCM7 in NPCs (*n* = 3, three different donors). **e** The mRNA expression of MCM7 in NPCs treated as described above was detected by qRT–PCR (*n* = 3, three different donors for three individual experiments; NS, no significance; *P < 0.05; **P < 0.01). **f** and **g** Western blot analysis of MCM7 in circKIF18A-silenced NPCs at 2 h intervals under CHX (20 μM)-mediated protein synthesis inhibition (*n* = 3, three different donors; *P < 0.05; **P < 0.01). **h** and **i** The effect of circKIF18A overexpression on the MCM7 level in NPCs treated with CHX at 2 h intervals (*n* = 3, three different donors; *P < 0.05; **P < 0.01). **j** Representative immunofluorescence images of NPCs transfected with sh-circKIF18A #1, sh-circKIF18A #2, circKIF18A, and empty vector lentiviruses (*n* = 5, five different donors; scale bar: 10 μm). **k** The proteasome inhibitor MG132 abolished the effect of sh-circKIF18A on reducing the MCM7 protein level in NPCs (*n* = 3, three different donors). **l** and **m** The ubiquitination level of MCM7 in circKIF18A-silenced or circKIF18A-overexpressing NPCs treated with MG132 (*n* = 3, three different donors). All data are presented as the mean ± SD values.

Based on the functions of circKIF18A shown above, we performed a pulldown assay followed by mass spectrometry to examine the potential RBPs binding to circKIF18A and further explored the mechanism underlying the role of circKIF18A in NPCs (Fig. 4a). In analyzing the RNA immunoprecipitation (RIP)-mass spectrometry results, we removed proteins of the negative control probe group from the circKIF18A protein set (Supplementary Fig. 2a and Supplementary Tables 2–4). In the ranking list, the top-ranked protein was MCM7, with a score of 150.66 (Fig. 4b). We then performed qRT–PCR after RIP and found that circKIF18A was enriched by the anti-MCM7 antibody and that circKIF18A overexpression promoted circKIF18A enrichment in NPCs (Fig. 4c), suggesting that circKIF18A interacts with MCM7 in NPCs.

To further investigate the effect of circKIF18A on MCM7, we transfected NPCs with sh-circKIF18A and circKIF18A overexpression lentiviruses to determine the MCM7 protein and mRNA levels. As shown in Fig. 4d, circKIF18A knockdown decreased the MCM7 protein level, which was increased in circKIF18A-overexpressing NPCs. However, the qRT-PCR results showed that circKIF18A overexpression did not significantly change the MCM7 mRNA level, indicating that circKIF18A might regulate MCM7 expression posttranscriptionally (Fig. 4e). Treatment with cycloheximide (CHX), a protein synthesis inhibitor, led to faster degradation of MCM7 in circKIF18A-silenced NPCs than that in vector group (Fig. 4f, g), and circKIF18A overexpression slowed the degradation rate of MCM7 in NPCs (Fig. 4h, i). In addition, immunofluorescence staining showed that MCM7 expression was enhanced by circKIF18A overexpression (Fig. 4j).

It has been reported that MCM7 degradation is involved in the ubiquitin–proteasome pathway^26^. Based on our results in this study, we presumed that circKIF18A might increase the MCM7 protein level by suppressing its ubiquitin-mediated proteasomal degradation. To investigate this possibility, we treated circKIF18A-silenced NPCs with or without MG132, a proteasome inhibitor, to measure the MCM7 protein level and found that MG132 treatment increased the MCM7 level in circKIF18A-silenced NPCs (Fig. 4k). Additionally, MCM7 was more ubiquitinated in circKIF18A-silenced cells and less ubiquitinated in circKIF18A-overexpressing NPCs (Fig. 4l, m). Together, these data indicate that circKIF18A increases the MCM7 protein level in NPCs by inhibiting its ubiquitin-mediated proteasomal degradation.

### CircKIF18A interacts with MCM7 and prohibits its degradation by UBE3A

**Fig. 5:**
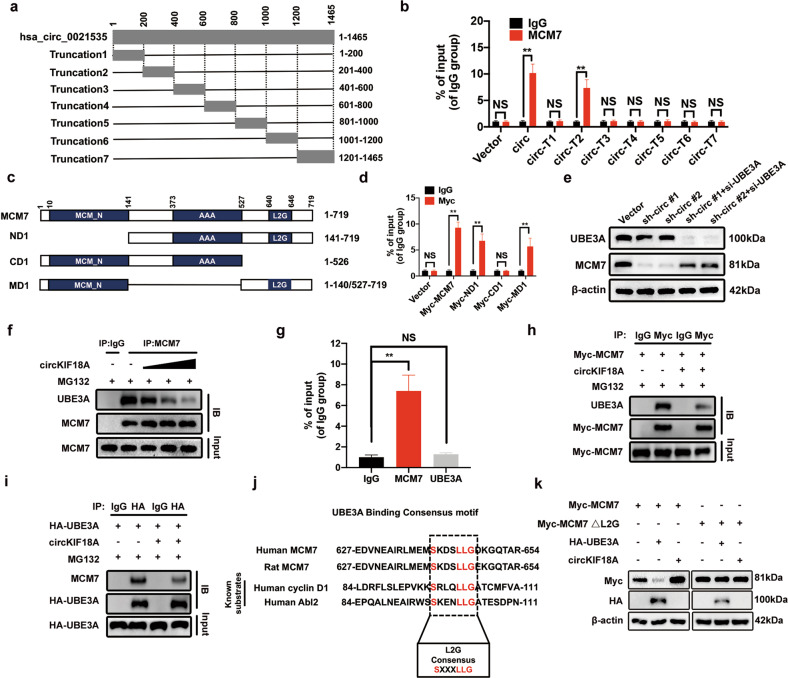
CircKIF18A prevents UBE3A-mediated degradation of MCM7 in NPCs. **a** A schematic showing six constructs, including full-length circKIF18A and truncations with different regions of circKIF18A. **b** RIP showed that the full-length and truncation 2 (circ-T2) mutant of circKIF18A interacted with MCM7 in HEK-293T cells. HEK-293T cells were separately transfected with plasmids containing full-length circKIF18A, its truncations and empty vector (*n* = 3; NS no significance; *P < 0.05; **P < 0.01). **c** Schematic illustration of full-length MCM7 and its three constructs containing different domains. **d** The results of RIP showed that only the CD1 mutant construct did not interact with circKIF18A. HEK-293T cells were separately transfected with plasmids containing full-length Myc-MCM7; plasmids containing the Myc-ND1, Myc-CD1, and Myc-MD1 mutant constructs; or empty vector (*n* = 3; NS no significance; *P < 0.05; **P < 0.01). **e** The effect of UBE3A on the MCM7 protein level in circKIF18A-silenced NPCs was detected using western blot analysis (*n* = 3, three different donors). **f** IP analysis of the interaction between UBE3A and MCM7 in NPCs transfected with increasing concentrations of the circKIF18A lentivirus (*n* = 3, three different donors). **g** RIP confirmed that circKIF18A directly interacted with MCM7 but not UBE3A in NPCs (*n* = 3, three different donors for three individual experiments; NS no significance; *P < 0.05; **P < 0.01). **h** and **i** IP analysis of the interaction between UBE3A and MCM7 in HEK-293T cells transfected with plasmids encoding circKIF18A, Myc-MCM7 or HA-UBE3A in NPCs (*n* = 3). **j** Sequence alignment between the UBE3A-binding motif in MCM7 and those in other known UBE3A substrates. **k** HEK-293T cells were transfected with plasmids encoding Myc-MCM7, Myc-MCM7△L2G, HA-UBE3A, or circKIF18A. Subsequent western blot analyses were performed to determine Myc-tagged MCM7 and HA-tagged UBE3A levels (*n* = 3). All data are presented as the mean ± SD values.

As shown in Supplementary Fig. 3a–c, we predicted the mutual binding sites in circKIF18A and MCM7 via the catRAPID database (http://service.tartaglialab.com/page/catrapid_group). To identify the region in circKIF18A that interacts with MCM7, a series of circKIF18A truncations were constructed to map circKIF18A regions (Fig. 5a), and the results of RIP showed that the full length and truncation 2 (circ-T2) of circKIF18A were enriched by the anti-MCM7 antibody (Fig. 5b). To investigate the circKIF18A-binding regions in MCM7, three deletion mutation constructs named ND1 (deletion of one N-terminal domain), CD1 (deletion of one C-terminal domain), and MD1 (deletion of the 142-526 amino acid (aa) domain) were constructed (Fig. 5c), and RIP was then performed to evaluate their binding capacity to circKIF18A. Our results showed that only the CD1 construct could not bind to circKIF18A, suggesting that the 527–719 aa region of MCM7 was necessary for its interaction with circKIF18A (Fig. 5d). It has been documented that ubiquitin ligase E3A (UBE3A) can ubiquitinate MCM7 through a specific homotypic motif^26^. Our western blot analysis results showed that UBE3A knockdown increased the MCM7 level, which was decreased by circKIF18A knockdown in NPCs (Fig. 5e, Supplementary Fig. 4a, b). In addition, the interaction between UBE3A and MCM7 was inhibited by circKIF18A overexpression in a dose-dependent manner (Fig. 5f). Therefore, we hypothesized that circKIF18A might compete with UBE3A to inhibit ubiquitin-mediated proteasomal degradation of MCM7 by disrupting the interaction between UBE3A and MCM7. To verify our hypothesis, we performed RIP and found that circKIF18A interacted with MCM7 but not UBE3A (Fig. 5g). Moreover, the interaction of MCM7 and UBE3A was suppressed by circKIF18A in NPCs (Fig. 5h, i). UBE3A substrates share a UBE3A-binding consensus motif, which is termed the L2G box^26^. The L2G box, conserved between the MCM7 amino acid sequences of human and rats, is located in the predicted circKIF18A-binding region of MCM7 (Fig. 5j and Supplementary Fig. 3c). Notably, mutation of the L2G box motif abolished the effects of UBE3A and circKIF18A on the exogenous MCM7 protein level, demonstrating that the L2G box motif overlaps with the circKIF18A-binding region (Fig. 5k and Supplementary Fig. 3d). Together, these data indicate that circKIF18A interacts with MCM7 and inhibits the degradation of MCM7 by competing with UBE3A.

### MCM7 mediates the effects of circKIF18A on NPC degeneration by regulating senescence under oxidative stress

**Fig. 6:**
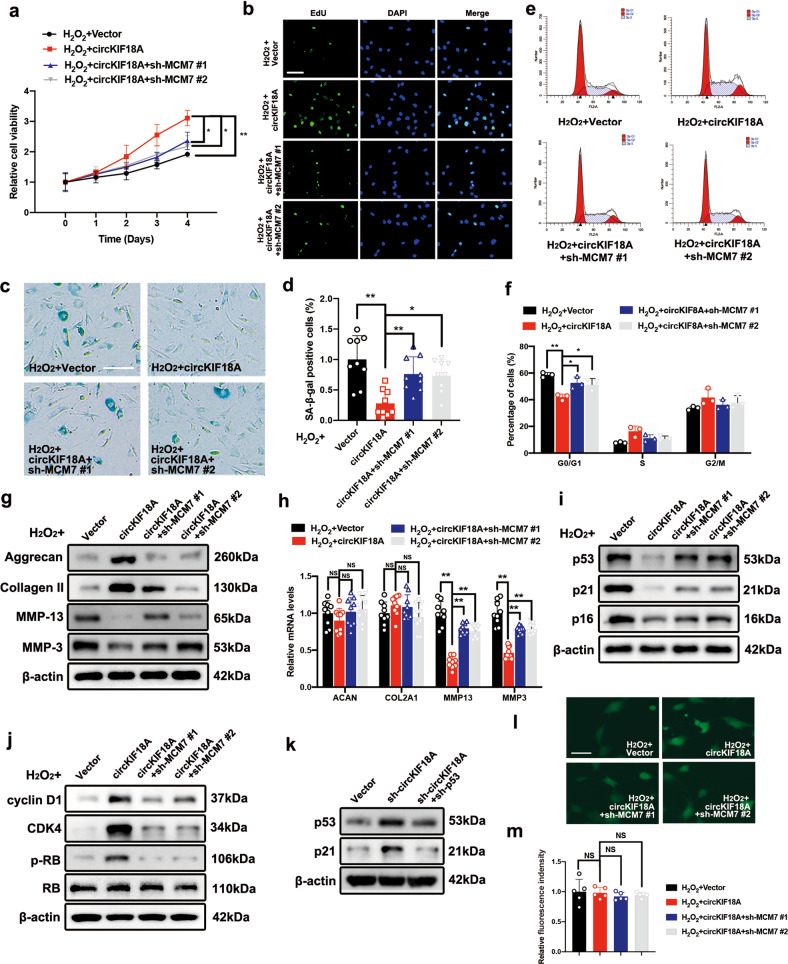
MCM7 mediates the effects of circKIF18A on NPC senescence under oxidative stress. **a** The CCK-8 assay showed the effects of circKIF18A and sh-MCM7 on NPC viability (*n* = 3, three different donors; *P < 0.05; **P < 0.01). **b** The EdU assay revealed that MCM7 mediated the effects of circKIF18A on proliferation in H_2_O_2_-treated NPCs (*n* = 5, five different donors; scale bar: 50 μm). **c** and **d** SA-β-gal staining assay of NPCs transfected with or without circKIF18A and sh-MCM7 lentiviruses under oxidative stress (*n* = 3, three different donors for three individual experiments; *P < 0.05, **P < 0.01; scale bar: 50 μm). **e** and **f** MCM7 knockdown promoted G0/G1 arrest, which was rescued by circKIF18A overexpression, in NPCs under oxidative stress (*n* = 3, three different donors; *P < 0.05; **P < 0.01). **g** The protein levels of aggrecan, collagen II, MMP-13, and MMP-3 in NPCs transfected with circKIF18A, sh-MCM7, and empty lentiviruses were determined using western blotting (n = 3, three different donors). **h** The qRT-PCR assay showed the effects of MCM7 on the mRNA expression of *ACAN*, *COL2A1*, *MMP13* and *MMP3* in circKIF18A-overexpressing NPCs (*n* = 3, three different donors for three individual experiments; NS, no significance; *P < 0.05; **P < 0.01). **i** The protein levels of p53, p21, and p16 were measured using western blotting. NPCs were stably transfected with circKIF18A, sh-MCM7, and empty lentiviruses (*n* = 3, three different donors). **j** Western blot assays showed the effects of MCM7 on the protein levels of cyclin D1, CDK4, p-RB and RB in NPCs treated as described above (*n* = 3, three different donors). **k** The effect of p53 knockdown on the p21 level in sh-circKIF18A NPCs (*n* = 3, three different donors). **l** and **m** Representative images of the DCFH-DA staining assay in NPCs with circKIF18A overexpression or MCM7 knockdown under oxidative stress (*n* = 5, five different donors; NS no significance. Scale bar: 20 μm). All data are presented as the mean ± SD values.

We next investigated whether MCM7 is necessary for the functions of circKIF18A in NPC degeneration. First, MCM7 knockdown inhibited the high NPC viability mediated by circKIF18A under oxidative stress (Fig. 6a, Supplementary Fig. 5a, b). Then, we performed an EdU assay and found that MCM7 knockdown decreased the proliferative capacity of circKIF18A-overexpressing NPCs treated with H_2_O_2_ (Fig. 6b). The results of the SA-β-gal assay showed that the number of SA-β-gal-positive cells was elevated by MCM7 knockdown in circKIF18A-overexpressing NPCs, implying that MCM7 mediates the effects of circKIF18A on NPC senescence under oxidative stress (Fig. 6c, d). Additionally, the percentage of cells in G0/G1 phase was reduced by circKIF18A overexpression, and MCM7 knockdown partially abolished the inhibitory effect of circKIF18A on cell cycle progression under oxidative stress (Fig. 6e, f). Furthermore, circKIF18A overexpression promoted ECM anabolism and prohibited ECM catabolism, whereas MCM7 knockdown partially inhibited the effects of circKIF18A in H_2_O_2_-treated NPCs (Fig. 6g). Similar to the results shown in Fig. 3h, i, MCM7 knockdown promoted the mRNA expression of *MMP13* and *MMP3* but not *ACAN* and *COL2A1* (Fig. 6h). It has been reported that the p53-p21 and p16 pathways are two major pathways involved in NPC senescence^27^. To explore the specific mechanism of the circKIF18A-MCM7 axis in senescence, we determined the protein levels of p53, p21 and p16 in circKIF18A-overexpressing NPCs with or without MCM7 knockdown. As shown in Fig. 6i, MCM7 knockdown alleviated circKIF18A-induced inhibition of p53, p21, and p16 expression. In addition, we evaluated the proteins downstream of p21 and p16, such as cyclin D1, cyclin-dependent kinase 4 (CDK4), and retinoblastoma protein (RB), and found that MCM7 knockdown downregulated cyclin D1 and CDK4 expression and inhibited RB phosphorylation (Fig. 6j). A previous study demonstrated that p21 activation can occur in either a p53-dependent or p53-independent manner^28^. In Fig. 6k, the western blot results show that p53 knockdown reduced the p21 level in circKIF18A-silenced NPCs, suggesting that circKIF18A regulates the classical p53-p21 signaling pathway. To evaluate the effect of the circKIF18A-MCM7 axis on the cellular ROS level, we performed DCFH-DA staining and found that the circKIF18A-MCM7 axis showed no predominant effect on the DCFH-DA intensity in NPCs under oxidative stress (Fig. 6l, m), suggesting that the circKIF18A-MCM7 axis alleviates NPC degeneration without affecting the cellular ROS level and that the beneficial role of the circKIF18A-MCM7 axis can be attributed to suppression of senescence-related pathways in NPCs. Thus, these results indicate that circKIF18A prohibits NPC senescence in a MCM7-dependent manner by regulating classical senescence signaling pathways.

### CircKIF18A regulates IVDD in a rat puncture-induced IVDD model

**Fig. 7:**
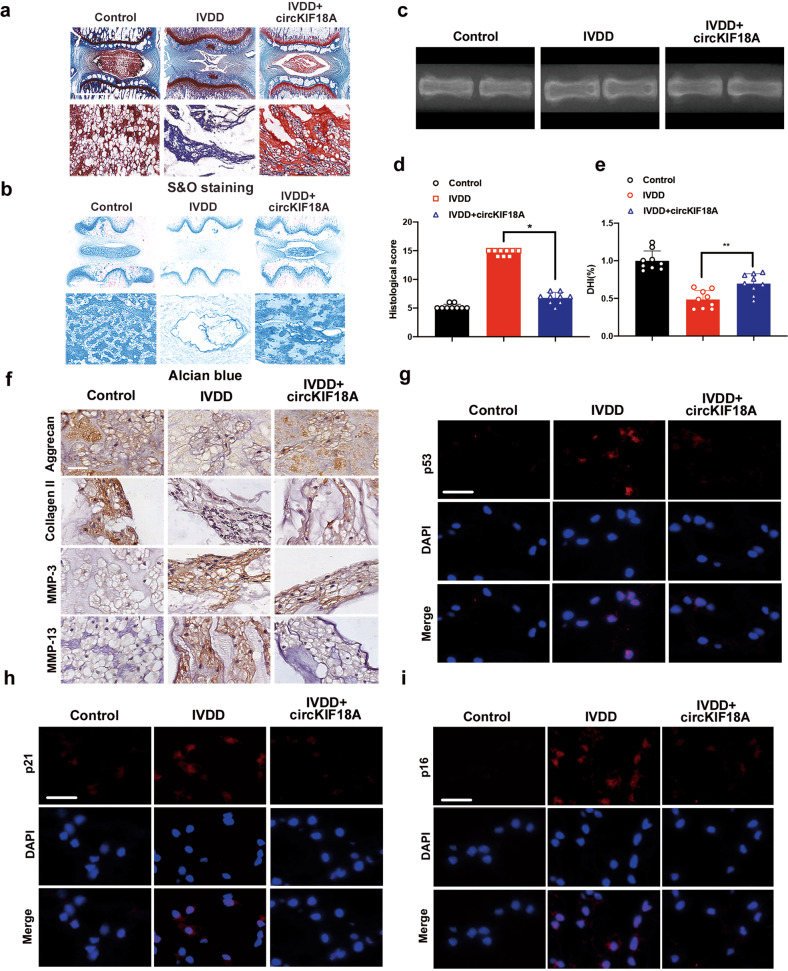
CircKIF18A ameliorates IVDD in a needle puncture rat model. **a** S&O staining revealed the effects of circKIF18A overexpression adenovirus treatment on IVDD in vivo (*n* = 9; scale bar: 1000 μm). **b** Alcian blue staining revealed the effects of circKIF18A overexpression adenovirus treatment on IVDD in rats (*n* = 9; scale bar: 1000 μm). **c** The effects of circKIF18A overexpression adenovirus treatment on disc height in rats were determined using X-ray imaging (*n* = 9). **d** The statistical graph of histological scores in different groups according to S&O staining (*n* = 9; *P < 0.05; **P < 0.01). **e** The statistical graph of the disc height index in different groups according to X-ray imaging (*n* = 9; *P < 0.05; **P < 0.01). **f** The effects of circKIF18A on ECM metabolism in rat IVDs (*n* = 5; scale bar: 50 μm). **g** CircKIF18A overexpression adenovirus treatment increased the p53 level in NPs (*n* = 5; scale bar: 50 μm). **h** Representative images of immunofluorescence staining for p21 in punctured NPs (*n* = 5; scale bar: 50 μm). **i** Representative images of immunofluorescence staining for p16 in punctured NPs (*n* = 5; scale bar: 50 μm). All data are presented as the mean ± SD values.

Because human circKIF18A contains a homologous interaction region with rat MCM7 according to the catRAPID database (Supplementary Figs. 3a–c and 6a–c), we applied the human circKIF18A overexpression adenovirus in rat models of puncture-induced IVDD as previously described^29^. First, we successfully overexpressed circKIF18A in rat tail discs (Supplementary Fig. 6d). The results of safranin O and fast green (S&O) staining and alcian blue staining showed that circKIF18A overexpression attenuated structural disc degeneration and increased the proteoglycan content (Fig. 7a, b, d). Additionally, circKIF18A adenovirus injection reduced disc height loss compared with that in the IVDD group (Fig. 7c, e). Next, the question of whether circKIF18A regulates MCM7 expression in rats needed to be addressed. As shown in Supplementary Fig. 6e, injection of circKIF18A overexpression adenovirus increased the reduced MCM7 level in punctured NPs. Next, we performed immunohistochemistry and found that circKIF18A promoted ECM anabolism and inhibited ECM catabolism in degenerative NPs (Fig. 7f). Moreover, our immunofluorescence results showed that overexpression of circKIF18A reduced the p53, p21, and p16 levels in vivo (Fig. 7g–i). Therefore, our data suggest that circKIF18A ameliorates IVDD by increasing the MCM7 level and inhibiting senescence in NPCs.

## Discussion

With the development of bioinformatics and genomics techniques, numerous circRNAs have been identified in human diseases, and their functions and molecular mechanisms have been gradually elucidated. Our previous study demonstrated that circTADA2A is able to serve as a competing endogenous RNA to accelerate osteosarcoma development by regulating the c-JUN-mediated signaling pathway and that CircSERPINE2 attenuates osteoarthritis by sponging miR-1271 in chondrocytes^11,30^. Moreover, circVMA21 and circERCC2 protect NPCs from degeneration by acting as miRNA sponges^31,32^. CircRNAs, which are highly stable and conserved among species, have diverse regulatory mechanisms due to their specific covalent closed-loop structure. However, except for studies focusing on sponging miRNAs, no study has investigated the novel mechanism of circRNAs in IVDD. Hence, more research is urgently needed to explore the novel role of circRNAs in IVDD and solve the mysteries of IVDD.

Although a number of circRNA functions are still unknown, compelling evidence has shown that circRNAs can interact with RNAs or proteins to affect gene transcription or regulate posttranslational modification by forming complexes in the nucleus or cytoplasm^33–35^. In this study, we found that circKIF18A overexpression increased the protein level of MCM7 by reinforcing its stability. Furthermore, MCM7 acts as a critical regulator of the cell cycle and is well investigated in the field of oncology^17,36^. A previous study showed that the E3 ubiquitin ligase UBE3A can accelerate MCM7 degradation via ubiquitination^26^. We demonstrated that circKIF18A suppress ubiquitin-mediated proteasomal degradation of MCM7 by competing with UBE3A and that the UBE3A-binding motif overlaps with the circKIF18A-binding site. Thus, we concluded that circKIF18A interacts with MCM7 and competitively prohibits UBE3A-induced MCM7 degradation.

Cellular senescence is a durable cell cycle arrest induced by stimuli such as irradiation, toxins, and inflammation^37^. Broadly, the mechanism underlying senescence is considered to involve two classical pathways: the p53-p21 and p16 signaling pathways. To date, MCM7 has been investigated almost solely in cancer, and there is a lack of research on its role in degenerative diseases^36,38^. In this study, we demonstrated that MCM7 mediates the effects of circKIF18A on NPC senescence and that the circKIF18A-MCM7 axis suppresses senescence via two classical signaling pathways—the p53-p21 and p16 signaling pathways.

Aggrecan and collagen II, two major ECM components in NPs, maintain hydrostatic pressure in discs to promote biomechanical balance, and they are proven to be substrates of matrix metalloproteinases (MMPs), such as MMP-3 and MMP-13, in IVDs^39,40^. Most studies have shown that changes in the protein levels of aggrecan and collagen II are the same as those in their mRNA levels^5^. In our study, the circKIF18A-MCM7 axis affected only the protein levels of aggrecan and collagen II and not their mRNA levels, a phenomenon that can be explained by the observation that the circKIF18A-MCM7 axis upregulates the expression levels of these proteins by inhibiting catabolic enzymes (MMP-3 and MMP-13).

This study suggests that NPC senescence is associated with oxidative stress in IVDD and that circKIF18A, as a biomarker of IVDD, alleviates NPC senescence by inhibiting MCM7 degradation and protecting against IVDD. Therefore, our study provides a solid foundation for improving our understanding of IVDD and augments molecular therapeutic approaches for IVDD.

## Supplementary information

Supplementary Information
