# Supplementary material for: Oxidative stress-induced circKIF18A downregulation impairs MCM7-mediated anti-senescence in intervertebral disc degeneration
Source: Exp Mol Med. 2022 Mar 24;54(3):285–97. doi: 10.1038/s12276-022-00732-0 (PMC8979962; doi:10.1038/s12276-022-00732-0)
Supplement: Supplementary file 1 — Supplementary Information [file 12276_2022_732_MOESM1_ESM.pdf]

**Supplementary Table 1. The top 250 circRNAs in GSE67566 dataset.**

| ID | adj.P.Val | P.Value | logFC | SPOT_ID |
|---|---|---|---|---|
| ASCRP0021 | 3.92E-15 | 5.40E-17 | 2.98 | hsa_circRNA_101852 |
| ASCRP0021 | 6.98E-16 | 3.80E-19 | 2.93 | hsa_circRNA_101853 |
| ASCRP0015 | 6.98E-16 | 5.39E-19 | 2.92 | hsa_circRNA_101139 |
| ASCRP0041 | 1.72E-15 | 8.20E-18 | 2.86 | hsa_circRNA_103890 |
| ASCRP0053 | 3.87E-14 | 2.07E-15 | 2.84 | hsa_circRNA_400019 |
| ASCRP0026 | 1.00E-15 | 2.18E-18 | 2.78 | hsa_circRNA_102324 |
| ASCRP0049 | 1.24E-15 | 3.47E-18 | 2.72 | hsa_circRNA_104703 |
| ASCRP0009 | 1.57E-15 | 5.42E-18 | 2.68 | hsa_circRNA_100604 |
| ASCRP0048 | 7.51E-15 | 1.69E-16 | 2.68 | hsa_circRNA_104600 |
| ASCRP0004 | 1.67E-15 | 6.33E-18 | 2.61 | hsa_circRNA_100018 |
| ASCRP0037 | 6.98E-16 | 1.15E-18 | 2.59 | hsa_circRNA_103410 |
| ASCRP0000 | 2.32E-14 | 9.78E-16 | 2.56 | hsa_circRNA_000200 |
| ASCRP0040 | 1.18E-14 | 3.35E-16 | 2.54 | hsa_circRNA_103801 |
| ASCRP0018 | 3.03E-15 | 3.14E-17 | 2.52 | hsa_circRNA_101525 |
| ASCRP0000 | 6.98E-16 | 9.22E-19 | 2.49 | hsa_circRNA_000178 |
| ASCRP0030 | 2.99E-14 | 1.47E-15 | 2.46 | hsa_circRNA_102694 |
| ASCRP0045 | 1.92E-14 | 7.34E-16 | 2.34 | hsa_circRNA_104315 |
| ASCRP0004 | 7.03E-14 | 4.45E-15 | 2.32 | hsa_circRNA_100086 |
| ASCRP0002 | 3.39E-15 | 4.21E-17 | 2.31 | hsa_circRNA_001175 |
| ASCRP0013 | 2.63E-14 | 1.23E-15 | 2.27 | hsa_circRNA_100946 |
| ASCRP0003 | 2.46E-15 | 1.79E-17 | 2.26 | hsa_circRNA_001653 |
| ASCRP0053 | 1.40E-14 | 4.39E-16 | 2.24 | hsa_circRNA_400090 |
| ASCRP0003 | 2.82E-15 | 2.30E-17 | 2.22 | hsa_circRNA_001654 |
| ASCRP0000 | 2.29E-14 | 9.57E-16 | 2.22 | hsa_circRNA_000166 |
| ASCRP0038 | 3.37E-14 | 1.70E-15 | 2.22 | hsa_circRNA_103541 |
| ASCRP0028 | 1.24E-15 | 3.86E-18 | 2.18 | hsa_circRNA_102543 |
| ASCRP0007 | 2.92E-14 | 1.42E-15 | 2.18 | hsa_circRNA_100374 |
| ASCRP0027 | 1.16E-14 | 2.99E-16 | 2.17 | hsa_circRNA_102445 |
| ASCRP0022 | 9.06E-15 | 2.10E-16 | 2.15 | hsa_circRNA_101879 |
| ASCRP0022 | 2.46E-15 | 1.76E-17 | 2.14 | hsa_circRNA_101914 |
| ASCRP0028 | 4.68E-15 | 7.28E-17 | 2.12 | hsa_circRNA_102513 |
| ASCRP0010 | 2.34E-15 | 1.50E-17 | 2.07 | hsa_circRNA_100646 |
| ASCRP0004 | 2.82E-15 | 2.26E-17 | 2.07 | hsa_circRNA_100085 |
| ASCRP0043 | 4.68E-15 | 7.13E-17 | 2.05 | hsa_circRNA_104086 |
| ASCRP0016 | 4.78E-15 | 7.76E-17 | 2.03 | hsa_circRNA_101336 |
| ASCRP0001 | 3.31E-15 | 3.72E-17 | 2.02 | hsa_circRNA_000881 |
| ASCRP0029 | 5.85E-15 | 1.05E-16 | 2.02 | hsa_circRNA_102639 |
| ASCRP0026 | 1.25E-14 | 3.76E-16 | 2.02 | hsa_circRNA_102380 |
| ASCRP0036 | 1.73E-14 | 6.02E-16 | 2.01 | hsa_circRNA_103345 |
| ASCRP0044 | 8.10E-14 | 5.46E-15 | 2.01 | hsa_circRNA_104166 |
| ASCRP0009 | 1.82E-14 | 6.53E-16 | 2 | hsa_circRNA_100571 |
| ASCRP0040 | 3.23E-15 | 3.46E-17 | 1.99 | hsa_circRNA_103749 |
| ASCRP0047 | 6.86E-15 | 1.46E-16 | 1.99 | hsa_circRNA_104503 |
| ASCRP0040 | 2.40E-14 | 1.04E-15 | 1.99 | hsa_circRNA_103712 |
| ASCRP0024 | 4.68E-15 | 7.17E-17 | 1.96 | hsa_circRNA_102093 |
| ASCRP0031 | 1.15E-14 | 2.91E-16 | 1.96 | hsa_circRNA_102844 |

| | | | | |
|---|---|---|---|---|
| ASCRP0012 | 1.72E-15 | 8.41E-18 | 1.95 | hsa_circRNA_100876 |
| ASCRP0053 | 6.86E-15 | 1.42E-16 | 1.95 | hsa_circRNA_400009 |
| ASCRP0026 | 9.57E-14 | 6.68E-15 | 1.95 | hsa_circRNA_102348 |
| ASCRP0001 | 2.91E-15 | 2.89E-17 | 1.92 | hsa_circRNA_000864 |
| ASCRP0027 | 7.11E-14 | 4.55E-15 | 1.92 | hsa_circRNA_102434 |
| ASCRP0033 | 1.35E-13 | 1.04E-14 | 1.92 | hsa_circRNA_103076 |
| ASCRP0006 | 1.72E-15 | 7.22E-18 | 1.9 | hsa_circRNA_100227 |
| ASCRP0029 | 2.34E-15 | 1.54E-17 | 1.9 | hsa_circRNA_102685 |
| ASCRP0001 | 3.09E-14 | 1.53E-15 | 1.88 | hsa_circRNA_000780 |
| ASCRP0028 | 1.00E-13 | 7.21E-15 | 1.88 | hsa_circRNA_102509 |
| ASCRP0024 | 2.77E-14 | 1.33E-15 | 1.87 | hsa_circRNA_102113 |
| ASCRP0022 | 1.69E-13 | 1.37E-14 | 1.82 | hsa_circRNA_101861 |
| ASCRP0037 | 1.18E-14 | 3.48E-16 | 1.81 | hsa_circRNA_103444 |
| ASCRP0025 | 3.85E-15 | 4.92E-17 | 1.8 | hsa_circRNA_102241 |
| ASCRP0002 | 1.86E-13 | 1.55E-14 | 1.8 | hsa_circRNA_001409 |
| ASCRP0023 | 6.52E-15 | 1.25E-16 | 1.78 | hsa_circRNA_101976 |
| ASCRP0037 | 6.86E-15 | 1.42E-16 | 1.78 | hsa_circRNA_103457 |
| ASCRP0024 | 3.87E-14 | 2.03E-15 | 1.77 | hsa_circRNA_102082 |
| ASCRP0039 | 1.18E-14 | 3.26E-16 | 1.76 | hsa_circRNA_103637 |
| ASCRP0004 | 3.87E-14 | 2.08E-15 | 1.75 | hsa_circRNA_100040 |
| ASCRP0015 | 1.60E-14 | 5.34E-16 | 1.71 | hsa_circRNA_101178 |
| ASCRP0018 | 5.94E-14 | 3.57E-15 | 1.71 | hsa_circRNA_101542 |
| ASCRP0005 | 1.40E-13 | 1.09E-14 | 1.7 | hsa_circRNA_100117 |
| ASCRP0027 | 6.86E-15 | 1.35E-16 | 1.69 | hsa_circRNA_102473 |
| ASCRP0005 | 6.86E-15 | 1.49E-16 | 1.69 | hsa_circRNA_100202 |
| ASCRP0009 | 7.88E-14 | 5.20E-15 | 1.67 | hsa_circRNA_100606 |
| ASCRP0022 | 7.92E-14 | 5.28E-15 | 1.67 | hsa_circRNA_101943 |
| ASCRP0001 | 1.61E-13 | 1.30E-14 | 1.66 | hsa_circRNA_001040 |
| ASCRP0039 | 5.72E-15 | 9.83E-17 | 1.65 | hsa_circRNA_103636 |
| ASCRP0041 | 1.18E-14 | 3.46E-16 | 1.64 | hsa_circRNA_103846 |
| ASCRP0016 | 1.37E-14 | 4.28E-16 | 1.64 | hsa_circRNA_101275 |
| ASCRP0032 | 4.11E-14 | 2.26E-15 | 1.64 | hsa_circRNA_102983 |
| ASCRP0053 | 7.92E-14 | 5.27E-15 | 1.63 | hsa_circRNA_400033 |
| ASCRP0008 | 1.15E-14 | 2.88E-16 | 1.62 | hsa_circRNA_100422 |
| ASCRP0022 | 6.95E-14 | 4.34E-15 | 1.61 | hsa_circRNA_101948 |
| ASCRP0025 | 1.60E-13 | 1.28E-14 | 1.6 | hsa_circRNA_102205 |
| ASCRP0045 | 2.74E-14 | 1.30E-15 | 1.58 | hsa_circRNA_104313 |
| ASCRP0001 | 1.80E-13 | 1.49E-14 | 1.58 | hsa_circRNA_000711 |
| ASCRP0000 | 6.02E-15 | 1.12E-16 | 1.57 | hsa_circRNA_000046 |
| ASCRP0001 | 1.36E-14 | 4.17E-16 | 1.57 | hsa_circRNA_000911 |
| ASCRP0017 | 2.70E-14 | 1.27E-15 | 1.57 | hsa_circRNA_101381 |
| ASCRP0030 | 1.60E-14 | 5.21E-16 | 1.56 | hsa_circRNA_102690 |
| ASCRP0018 | 1.60E-14 | 5.42E-16 | 1.56 | hsa_circRNA_101491 |
| ASCRP0008 | 3.55E-14 | 1.82E-15 | 1.56 | hsa_circRNA_100508 |
| ASCRP0050 | 3.45E-14 | 1.75E-15 | 1.54 | hsa_circRNA_104752 |
| ASCRP0002 | 8.65E-14 | 5.96E-15 | 1.54 | hsa_circRNA_001405 |
| ASCRP0015 | 1.74E-14 | 6.13E-16 | 1.53 | hsa_circRNA_101164 |
| ASCRP0016 | 7.35E-14 | 4.75E-15 | 1.53 | hsa_circRNA_101278 |

| | | | | |
|---|---|---|---|---|
| ASCRP0016 | 9.69E-14 | 6.80E-15 | 1.53 | hsa_circRNA_101290 |
| ASCRP0000 | 1.92E-14 | 7.21E-16 | 1.5 | hsa_circRNA_000662 |
| ASCRP0014 | 1.92E-14 | 7.76E-16 | 1.5 | hsa_circRNA_101040 |
| ASCRP0007 | 1.92E-14 | 7.73E-16 | 1.49 | hsa_circRNA_100329 |
| ASCRP0017 | 6.44E-14 | 3.96E-15 | 1.48 | hsa_circRNA_101373 |
| ASCRP0009 | 7.24E-14 | 4.65E-15 | 1.48 | hsa_circRNA_100525 |
| ASCRP0001 | 3.55E-14 | 1.83E-15 | 1.47 | hsa_circRNA_001046 |
| ASCRP0051 | 1.93E-14 | 7.87E-16 | 1.46 | hsa_circRNA_104950 |
| ASCRP0000 | 2.78E-14 | 1.35E-15 | 1.46 | hsa_circRNA_000684 |
| ASCRP0006 | 6.86E-15 | 1.40E-16 | 1.45 | hsa_circRNA_100236 |
| ASCRP0053 | 1.60E-14 | 5.25E-16 | 1.45 | hsa_circRNA_400040 |
| ASCRP0038 | 1.97E-14 | 8.18E-16 | 1.44 | hsa_circRNA_103563 |
| ASCRP0000 | 1.18E-13 | 8.80E-15 | 1.43 | hsa_circRNA_000638 |
| ASCRP0000 | 9.36E-14 | 6.50E-15 | 1.4 | hsa_circRNA_000598 |
| ASCRP0002 | 1.21E-13 | 9.14E-15 | 1.4 | hsa_circRNA_001153 |
| ASCRP0007 | 5.84E-14 | 3.45E-15 | 1.39 | hsa_circRNA_100411 |
| ASCRP0050 | 3.86E-14 | 2.02E-15 | 1.38 | hsa_circRNA_104759 |
| ASCRP0024 | 8.65E-14 | 5.95E-15 | 1.38 | hsa_circRNA_102122 |
| ASCRP0020 | 1.54E-13 | 1.21E-14 | 1.38 | hsa_circRNA_101722 |
| ASCRP0012 | 2.41E-14 | 1.07E-15 | 1.37 | hsa_circRNA_100845 |
| ASCRP0003 | 1.30E-13 | 9.96E-15 | 1.37 | hsa_circRNA_001950 |
| ASCRP0034 | 1.57E-13 | 1.23E-14 | 1.36 | hsa_circRNA_103137 |
| ASCRP0035 | 1.82E-14 | 6.62E-16 | 1.35 | hsa_circRNA_103285 |
| ASCRP0000 | 2.32E-14 | 9.87E-16 | 1.35 | hsa_circRNA_000671 |
| ASCRP0032 | 1.13E-13 | 8.34E-15 | 1.31 | hsa_circRNA_102929 |
| ASCRP0001 | 9.23E-15 | 2.17E-16 | 1.3 | hsa_circRNA_000791 |
| ASCRP0037 | 4.33E-14 | 2.41E-15 | 1.29 | hsa_circRNA_103456 |
| ASCRP0006 | 7.11E-14 | 4.52E-15 | 1.29 | hsa_circRNA_100223 |
| ASCRP0002 | 8.07E-14 | 5.41E-15 | 1.29 | hsa_circRNA_001100 |
| ASCRP0004 | 5.28E-14 | 3.08E-15 | 1.28 | hsa_circRNA_100045 |
| ASCRP0046 | 6.21E-14 | 3.78E-15 | 1.28 | hsa_circRNA_104339 |
| ASCRP0000 | 7.55E-14 | 4.94E-15 | 1.28 | hsa_circRNA_000629 |
| ASCRP0004 | 1.92E-14 | 7.56E-16 | 1.25 | hsa_circRNA_100090 |
| ASCRP0027 | 3.87E-14 | 2.09E-15 | 1.25 | hsa_circRNA_102431 |
| ASCRP0016 | 5.84E-14 | 3.47E-15 | 1.23 | hsa_circRNA_101322 |
| ASCRP0030 | 1.77E-13 | 1.46E-14 | 1.21 | hsa_circRNA_102746 |
| ASCRP0027 | 1.91E-13 | 1.60E-14 | 1.2 | hsa_circRNA_102465 |
| ASCRP0047 | 2.24E-13 | 1.94E-14 | 1.15 | hsa_circRNA_104499 |
| ASCRP0023 | 9.88E-14 | 7.00E-15 | 1.13 | hsa_circRNA_101975 |
| ASCRP0030 | 8.65E-14 | 5.97E-15 | 1.12 | hsa_circRNA_102689 |
| ASCRP0028 | 7.03E-14 | 4.43E-15 | 1.11 | hsa_circRNA_102579 |
| ASCRP0015 | 1.60E-13 | 1.27E-14 | 1.06 | hsa_circRNA_101142 |
| ASCRP0032 | 5.84E-14 | 3.49E-15 | 1.03 | hsa_circRNA_102984 |
| ASCRP0009 | 2.11E-13 | 1.79E-14 | -1 | hsa_circRNA_100620 |
| ASCRP0014 | 1.02E-13 | 7.34E-15 | -1.02 | hsa_circRNA_101094 |
| ASCRP0003 | 6.36E-14 | 3.89E-15 | -1.06 | hsa_circRNA_001583 |
| ASCRP0022 | 4.47E-14 | 2.50E-15 | -1.08 | hsa_circRNA_101891 |
| ASCRP0028 | 1.75E-13 | 1.43E-14 | -1.09 | hsa_circRNA_102570 |

| | | | | |
|---|---|---|---|---|
| ASCRP0029 | 1.92E-13 | 1.62E-14 | -1.09 | hsa_circRNA_102644 |
| ASCRP0051 | 4.11E-14 | 2.26E-15 | -1.15 | hsa_circRNA_104929 |
| ASCRP0000 | 8.65E-14 | 5.98E-15 | -1.21 | hsa_circRNA_000676 |
| ASCRP0019 | 6.67E-14 | 4.13E-15 | -1.25 | hsa_circRNA_101555 |
| ASCRP0007 | 7.55E-14 | 4.96E-15 | -1.26 | hsa_circRNA_100332 |
| ASCRP0023 | 5.70E-14 | 3.35E-15 | -1.28 | hsa_circRNA_102039 |
| ASCRP0029 | 4.68E-14 | 2.65E-15 | -1.29 | hsa_circRNA_102660 |
| ASCRP0035 | 1.17E-13 | 8.69E-15 | -1.29 | hsa_circRNA_103225 |
| ASCRP0018 | 1.83E-14 | 6.70E-16 | -1.31 | hsa_circRNA_101511 |
| ASCRP0004 | 1.25E-13 | 9.47E-15 | -1.31 | hsa_circRNA_100051 |
| ASCRP0033 | 9.88E-14 | 7.00E-15 | -1.34 | hsa_circRNA_103039 |
| ASCRP0039 | 1.13E-13 | 8.35E-15 | -1.34 | hsa_circRNA_103645 |
| ASCRP0015 | 4.99E-14 | 2.88E-15 | -1.35 | hsa_circRNA_101192 |
| ASCRP0009 | 1.08E-13 | 7.89E-15 | -1.36 | hsa_circRNA_100616 |
| ASCRP0018 | 3.17E-14 | 1.58E-15 | -1.37 | hsa_circRNA_101531 |
| ASCRP0036 | 3.95E-14 | 2.14E-15 | -1.37 | hsa_circRNA_103311 |
| ASCRP0027 | 3.36E-14 | 1.69E-15 | -1.38 | hsa_circRNA_102422 |
| ASCRP0012 | 1.35E-13 | 1.05E-14 | -1.38 | hsa_circRNA_100836 |
| ASCRP0038 | 1.82E-13 | 1.52E-14 | -1.39 | hsa_circRNA_103547 |
| ASCRP0017 | 1.60E-14 | 5.53E-16 | -1.41 | hsa_circRNA_101407 |
| ASCRP0006 | 2.19E-13 | 1.88E-14 | -1.41 | hsa_circRNA_100270 |
| ASCRP0039 | 1.18E-14 | 3.19E-16 | -1.42 | hsa_circRNA_103611 |
| ASCRP0004 | 1.26E-13 | 9.54E-15 | -1.43 | hsa_circRNA_100035 |
| ASCRP0010 | 6.12E-14 | 3.70E-15 | -1.44 | hsa_circRNA_100640 |
| ASCRP0027 | 6.86E-15 | 1.49E-16 | -1.45 | hsa_circRNA_102456 |
| ASCRP0001 | 1.36E-14 | 4.13E-16 | -1.45 | hsa_circRNA_001059 |
| ASCRP0003 | 2.55E-14 | 1.18E-15 | -1.45 | hsa_circRNA_001754 |
| ASCRP0028 | 2.36E-14 | 1.01E-15 | -1.46 | hsa_circRNA_102483 |
| ASCRP0011 | 9.93E-14 | 7.07E-15 | -1.47 | hsa_circRNA_100790 |
| ASCRP0018 | 7.96E-15 | 1.82E-16 | -1.48 | hsa_circRNA_101470 |
| ASCRP0032 | 2.41E-14 | 1.06E-15 | -1.48 | hsa_circRNA_102990 |
| ASCRP0004 | 4.71E-14 | 2.69E-15 | -1.49 | hsa_circRNA_100034 |
| ASCRP0016 | 3.67E-14 | 1.90E-15 | -1.51 | hsa_circRNA_101258 |
| ASCRP0008 | 4.11E-14 | 2.27E-15 | -1.51 | hsa_circRNA_100420 |
| ASCRP0023 | 1.73E-13 | 1.40E-14 | -1.51 | hsa_circRNA_102018 |
| ASCRP0036 | 6.85E-14 | 4.26E-15 | -1.53 | hsa_circRNA_103384 |
| ASCRP0032 | 1.77E-13 | 1.46E-14 | -1.53 | hsa_circRNA_102899 |
| ASCRP0041 | 5.85E-15 | 1.04E-16 | -1.56 | hsa_circRNA_103812 |
| ASCRP0009 | 7.39E-14 | 4.80E-15 | -1.58 | hsa_circRNA_100579 |
| ASCRP0023 | 4.63E-14 | 2.61E-15 | -1.59 | hsa_circRNA_102020 |
| ASCRP0006 | 1.18E-14 | 3.14E-16 | -1.6 | hsa_circRNA_100272 |
| ASCRP0006 | 1.92E-14 | 7.60E-16 | -1.6 | hsa_circRNA_100213 |
| ASCRP0002 | 1.92E-14 | 7.43E-16 | -1.62 | hsa_circRNA_001401 |
| ASCRP0022 | 2.41E-14 | 1.08E-15 | -1.63 | hsa_circRNA_101889 |
| ASCRP0031 | 3.87E-14 | 2.07E-15 | -1.63 | hsa_circRNA_102817 |
| ASCRP0035 | 1.46E-14 | 4.64E-16 | -1.65 | hsa_circRNA_103220 |
| ASCRP0017 | 2.50E-14 | 1.14E-15 | -1.65 | hsa_circRNA_101429 |
| ASCRP0012 | 1.07E-13 | 7.78E-15 | -1.67 | hsa_circRNA_100919 |

| | | | | |
|---|---|---|---|---|
| ASCRP0023 | 4.73E-15 | 7.52E-17 | -1.68 | hsa_circRNA_102034 |
| ASCRP0024 | 3.92E-15 | 5.42E-17 | -1.71 | hsa_circRNA_102119 |
| ASCRP0015 | 1.60E-14 | 5.38E-16 | -1.74 | hsa_circRNA_101175 |
| ASCRP0042 | 1.16E-14 | 2.96E-16 | -1.76 | hsa_circRNA_103998 |
| ASCRP0045 | 4.68E-15 | 6.92E-17 | -1.77 | hsa_circRNA_104256 |
| ASCRP0053 | 9.56E-15 | 2.28E-16 | -1.78 | hsa_circRNA_400010 |
| ASCRP0022 | 1.01E-14 | 2.44E-16 | -1.8 | hsa_circRNA_101873 |
| ASCRP0000 | 1.25E-14 | 3.75E-16 | -1.8 | hsa_circRNA_000094 |
| ASCRP0010 | 6.02E-15 | 1.10E-16 | -1.81 | hsa_circRNA_100723 |
| ASCRP0029 | 7.17E-15 | 1.59E-16 | -1.83 | hsa_circRNA_102687 |
| ASCRP0022 | 1.18E-14 | 3.30E-16 | -1.84 | hsa_circRNA_101915 |
| ASCRP0018 | 4.17E-15 | 5.91E-17 | -1.86 | hsa_circRNA_101471 |
| ASCRP0025 | 1.72E-15 | 8.91E-18 | -1.89 | hsa_circRNA_102195 |
| ASCRP0030 | 2.19E-13 | 1.89E-14 | -1.93 | hsa_circRNA_102771 |
| ASCRP0046 | 5.04E-14 | 2.93E-15 | -1.94 | hsa_circRNA_104437 |
| ASCRP0016 | 2.91E-15 | 2.85E-17 | -1.96 | hsa_circRNA_101259 |
| ASCRP0042 | 2.91E-15 | 2.92E-17 | -1.96 | hsa_circRNA_103948 |
| ASCRP0006 | 1.10E-14 | 2.70E-16 | -1.96 | hsa_circRNA_100273 |
| ASCRP0033 | 1.26E-13 | 9.66E-15 | -1.96 | hsa_circRNA_103029 |
| ASCRP0013 | 1.18E-13 | 8.75E-15 | -2.01 | hsa_circRNA_100977 |
| ASCRP0006 | 2.47E-14 | 1.12E-15 | -2.02 | hsa_circRNA_100290 |
| ASCRP0021 | 4.84E-14 | 2.78E-15 | -2.02 | hsa_circRNA_101850 |
| ASCRP0017 | 2.02E-15 | 1.12E-17 | -2.05 | hsa_circRNA_101404 |
| ASCRP0037 | 2.40E-14 | 1.04E-15 | -2.05 | hsa_circRNA_103491 |
| ASCRP0010 | 3.39E-15 | 4.13E-17 | -2.12 | hsa_circRNA_100637 |
| ASCRP0003 | 5.72E-15 | 9.89E-17 | -2.12 | hsa_circRNA_002086 |
| ASCRP0008 | 1.90E-14 | 7.04E-16 | -2.12 | hsa_circRNA_100476 |
| ASCRP0022 | 5.25E-15 | 8.71E-17 | -2.15 | hsa_circRNA_101886 |
| ASCRP0001 | 1.53E-14 | 4.92E-16 | -2.2 | hsa_circRNA_000941 |
| ASCRP0007 | 3.31E-15 | 3.77E-17 | -2.22 | hsa_circRNA_100319 |
| ASCRP0013 | 1.35E-13 | 1.05E-14 | -2.29 | hsa_circRNA_100956 |
| ASCRP0010 | 2.34E-15 | 1.46E-17 | -2.3 | hsa_circRNA_100684 |
| ASCRP0012 | 8.50E-14 | 5.76E-15 | -2.3 | hsa_circRNA_100844 |
| ASCRP0010 | 1.00E-13 | 7.15E-15 | -2.3 | hsa_circRNA_100698 |
| ASCRP0031 | 1.60E-13 | 1.28E-14 | -2.3 | hsa_circRNA_102824 |
| ASCRP0001 | 1.18E-14 | 3.26E-16 | -2.31 | hsa_circRNA_000750 |
| ASCRP0051 | 6.52E-15 | 1.26E-16 | -2.41 | hsa_circRNA_104876 |
| ASCRP0038 | 3.92E-15 | 5.18E-17 | -2.46 | hsa_circRNA_103518 |
| ASCRP0026 | 1.19E-13 | 8.90E-15 | -2.5 | hsa_circRNA_102367 |
| ASCRP0051 | 2.73E-14 | 1.29E-15 | -2.56 | hsa_circRNA_104952 |
| ASCRP0024 | 1.77E-13 | 1.46E-14 | -2.64 | hsa_circRNA_102126 |
| ASCRP0039 | 2.82E-15 | 2.34E-17 | -2.65 | hsa_circRNA_103634 |
| ASCRP0028 | 1.00E-15 | 2.42E-18 | -2.68 | hsa_circRNA_102492 |
| ASCRP0011 | 3.39E-15 | 4.08E-17 | -2.69 | hsa_circRNA_100772 |
| ASCRP0034 | 2.84E-15 | 2.52E-17 | -2.7 | hsa_circRNA_103198 |
| ASCRP0034 | 1.60E-13 | 1.27E-14 | -2.7 | hsa_circRNA_103139 |
| ASCRP0029 | 1.92E-14 | 7.77E-16 | -2.72 | hsa_circRNA_102618 |
| ASCRP0008 | 2.50E-14 | 1.15E-15 | -2.76 | hsa_circRNA_100427 |

| | | | | |
|---|---|---|---|---|
| ASCRP0019 | 1.92E-14 | 7.77E-16 | -2.8 | hsa_circRNA_101558 |
| ASCRP0037 | 2.84E-15 | 2.55E-17 | -2.83 | hsa_circRNA_103486 |
| ASCRP0052 | 1.18E-14 | 3.24E-16 | -2.83 | hsa_circRNA_105031 |
| ASCRP0017 | 1.82E-14 | 6.52E-16 | -2.86 | hsa_circRNA_101370 |
| ASCRP0042 | 2.04E-13 | 1.72E-14 | -2.97 | hsa_circRNA_104019 |
| ASCRP0020 | 1.18E-14 | 3.40E-16 | -3.04 | hsa_circRNA_101709 |
| ASCRP0048 | 2.41E-14 | 1.07E-15 | -3.04 | hsa_circRNA_104630 |
| ASCRP0019 | 1.96E-14 | 8.06E-16 | -3.05 | hsa_circRNA_101557 |
| ASCRP0041 | 6.98E-16 | 1.21E-18 | -3.06 | hsa_circRNA_103838 |
| ASCRP0024 | 1.92E-14 | 7.37E-16 | -3.18 | hsa_circRNA_102116 |
| ASCRP0047 | 2.19E-13 | 1.87E-14 | -3.26 | hsa_circRNA_104508 |
| ASCRP0019 | 1.60E-14 | 5.49E-16 | -3.3 | hsa_circRNA_101645 |

**Supplementary Table 2. Proteins captured by negative control probe.**

| Gene Symbol | Protein_score | MW [kDa] | Coverage [%] |
|---|---|---|---|
| KRT1 | 23.17 | 66 | 19 |
| ACTB | 12.32 | 41.7 | 36 |
| MYH9 | 14.16 | 226.4 | 8 |
| MYH10 | 19.59 | 228.9 | 6 |
| RBM25 | 12.21 | 100.1 | 13 |
| KRT5 | 11.42 | 62.3 | 17 |
| | 9.68 | 134.1 | 9 |
| FLNA | 20.53 | 280.6 | 4 |
| SERBP1 | 13.56 | 49.1 | 13 |
| PLEC | 10.79 | 531.5 | 3 |
| DDX5 | 8.05 | 67.7 | 16 |
| KRT2 | 14.61 | 65.4 | 15 |
| KRT10 | 12.78 | 58.8 | 17 |
| ACTG2 | 3.76 | 41.9 | 23 |
| | 7.22 | 76.1 | 12 |
| CKAP4 | 13.2 | 58.1 | 12 |
| TUBB6 | 3.72 | 50.1 | 17 |
| TUBB4B | 15.86 | 49.8 | 17 |
| PCCA | 5.79 | 77.4 | 8 |
| DDX17 | 5.79 | 80.2 | 8 |
| PRPF8 | 12.45 | 273.4 | 3 |
| | 1.67 | 54.6 | 10 |
| HSP90AB1 | 5.87 | 83.2 | 10 |
| KRT9 | 9.95 | 62 | 8 |
| SF3B1 | 3.86 | 145.7 | 6 |
| | 2.37 | 68.8 | 11 |
| THRAP3 | 5.96 | 108.6 | 6 |
| KRT6A | 11.24 | 60 | 9 |
| DYNC1H1 | 4.03 | 532.1 | 1 |
| FIP1L1 | 5.34 | 66.5 | 8 |
| IRS1 | 3.74 | 131.5 | 6 |
| ERH | 10.19 | 12.3 | 34 |
| COL2A1 | 5.54 | 141.7 | 4 |
| HSP90B1 | 3.82 | 92.4 | 7 |
| KRT16 | 3.66 | 51.2 | 12 |
| ATP5B | 6.01 | 48.1 | 11 |
| VIM | 7.32 | 53.6 | 12 |
| TUBA1C | 10.08 | 57.7 | 10 |
| DDX41 | 4.63 | 69.8 | 6 |
| DHX9 | 4.7 | 140.9 | 3 |
| | 5.39 | 216.7 | 3 |
| C22orf28 | 5.62 | 55.2 | 10 |
| HNRNPK | 3.67 | 48.5 | 12 |
| | 3.83 | 60.7 | 8 |
| HSPA5 | 0 | 72.3 | 9 |
| MYL12B | 3.96 | 19.8 | 23 |

| | | | |
|---|---|---|---|
| AP2A1 | 1.73 | 107.5 | 5 |
| CLTC | 1.84 | 191.9 | 3 |
| | 3.81 | 77.5 | 8 |
| DHX36 | 4.07 | 100.1 | 5 |
| CNBP | 3.97 | 19.5 | 25 |
| EIF4A1 | 5.91 | 46.1 | 14 |
| NONO | 9.69 | 54.3 | 8 |
| DDX3X | 5.29 | 73.2 | 7 |
| PC | 5.48 | 129.6 | 4 |
| KRT14 | 3.66 | 51.5 | 8 |
| CAD | 3.88 | 242.8 | 2 |
| STK10 | 0 | 112.1 | 4 |
| PRPF6 | 6.25 | 106.9 | 4 |
| GLG1 | 3.33 | 134.5 | 4 |
| ATP1A1 | 0 | 112.4 | 5 |
| COL14A1 | 4.66 | 193.4 | 3 |
| | 5.55 | 17 | 25 |
| TLN1 | 0 | 269.6 | 2 |
| RPS3 | 1.67 | 26.7 | 21 |
| RAN | 2.98 | 26.2 | 15 |
| | 4.58 | 53 | 8 |
| EIF3A | 3.49 | 166.4 | 2 |
| | 0 | 52.2 | 8 |
| CAND1 | 1.9 | 136.3 | 4 |
| HIST1H1E | 2.06 | 21.9 | 19 |
| COPB2 | 3.63 | 102.4 | 4 |
| | 5.79 | 64 | 7 |
| HSP90AA1 | 1.71 | 84.6 | 6 |
| DDX46 | 3.78 | 100.3 | 3 |
| ALDOA | 2.91 | 39.4 | 13 |
| | 1.85 | 33.1 | 15 |
| LMNA | 2.29 | 65.1 | 8 |
| COPG1 | 6.23 | 97.7 | 4 |
| RBMS1 | 1.97 | 44.1 | 11 |
| FLNB | 3.33 | 280.3 | 2 |
| EFTUD2 | 1.88 | 108.1 | 4 |
| DDX42 | 3.85 | 102.9 | 4 |
| P4HA2 | 2.25 | 60.6 | 5 |
| | 5.64 | 121 | 3 |
| EIF2S3 | 4.21 | 51.1 | 7 |
| DCTN1 | 6.15 | 141.6 | 3 |
| P4HB | 4.35 | 55.3 | 5 |
| PFKM | 5.51 | 66.7 | 5 |
| CHERP | 1.71 | 103.6 | 4 |
| YBX1 | 2.99 | 35.9 | 15 |
| POLR2B | 5.41 | 125.1 | 3 |
| | 3.64 | 70.4 | 7 |
| PNN | 1.7 | 81.5 | 5 |

| | | | |
|---|---|---|---|
| YWHAB | 1.97 | 22.2 | 16 |
| | 0 | 81.7 | 5 |
| | 0 | 29.7 | 12 |
| TPM3 | 3.38 | 28.9 | 11 |
| HIST2H2AC | 4.36 | 14 | 20 |
| | 1.7 | 19.2 | 17 |
| ARCN1 | 4.29 | 57.2 | 6 |
| FUBP3 | 3.88 | 61.6 | 4 |
| PHB | 3.62 | 29.8 | 11 |
| SND1 | 1.86 | 99.6 | 4 |
| RPL6 | 1.62 | 32.7 | 10 |
| | 0 | 32.7 | 10 |
| PSMD3 | 1.66 | 58.5 | 6 |
| PCOLCE | 0 | 47.9 | 9 |
| RPS18 | 2 | 17.7 | 18 |
| RHOA | 1.94 | 21.8 | 16 |
| EIF4G2 | 3.45 | 110.2 | 3 |
| SRSF2 | 8.68 | 25.5 | 12 |
| PRPF40A | 1.86 | 92.8 | 4 |
| MYL6 | 5.96 | 26.7 | 10 |
| SAP18 | 0 | 17.6 | 21 |
| NUFIP2 | 3.79 | 76.1 | 5 |
| | 1.66 | 19.3 | 17 |
| | 1.69 | 28.8 | 12 |
| SRSF7 | 0 | 27.4 | 9 |
| CAPN2 | 3.99 | 32.9 | 9 |
| DHX15 | 1.86 | 89.5 | 3 |
| | 4.36 | 15 | 10 |
| | 3.3 | 27.9 | 10 |
| RPS2 | 2.42 | 31.4 | 10 |
| BUB3 | 3.69 | 37.1 | 9 |
| | 1.84 | 24 | 13 |
| PSMD2 | 3.74 | 100.1 | 3 |
| HNRNPA2B1 | 1.85 | 31.1 | 11 |
| | 2.32 | 32.8 | 8 |
| GARS | 0 | 77.5 | 4 |
| HNRNPC | 2.21 | 32 | 9 |
| CPSF7 | 0 | 52 | 6 |
| LEPRE1 | 1.98 | 68.9 | 4 |
| | 4.09 | 16.1 | 17 |
| RPL11 | 0 | 20.2 | 20 |
| TCP1 | 4.24 | 60.3 | 4 |
| DDX1 | 1.63 | 82.4 | 4 |
| HSPA6 | 0 | 71 | 3 |
| | 6.27 | 40.2 | 5 |
| SEC31A | 0 | 129 | 2 |
| FLNC | 3.4 | 290.8 | 1 |
| P4HA1 | 3.69 | 60.9 | 5 |

| | | | |
|---|---|---|---|
| IQGAP1 | 3.79 | 124.5 | 2 |
| KRT17 | 1.86 | 48.1 | 6 |
| IPO9 | 2.76 | 115.9 | 1 |
| KHDRBS2 | 3.55 | 35.3 | 6 |
| CDC5L | 1.92 | 92.2 | 3 |
| ACACA | 0 | 257.1 | 2 |
| SLK | 1.91 | 142.6 | 2 |
| SMC3 | 0 | 141.4 | 2 |
| | 1.61 | 32.4 | 8 |
| FUBP1 | 3.5 | 67.5 | 4 |
| RUVBL2 | 1.79 | 51.1 | 4 |
| CCT3 | 1.98 | 63.5 | 4 |
| SRSF1 | 1.91 | 25.6 | 12 |
| DPYSL2 | 1.76 | 62.2 | 3 |
| | 0 | 34.9 | 9 |
| MCCC2 | 1.77 | 61.3 | 5 |
| DDOST | 2.1 | 50.8 | 4 |
| | 0 | 49.8 | 6 |
| | 2.01 | 99.1 | 2 |
| YWHAZ | 1.97 | 27.7 | 9 |
| HNRNPA3 | 2.49 | 39.6 | 5 |
| EEF2 | 2 | 62.8 | 4 |
| USP16 | 0 | 93.5 | 3 |
| | 2.18 | 50.4 | 4 |
| AIFM1 | 3.86 | 66.9 | 3 |
| MCM4 | 2 | 96.5 | 2 |
| CDK11A | 2.44 | 91.3 | 2 |
| EEF1A1 | 2.11 | 50.2 | 8 |
| HNRNPF | 0 | 45.6 | 6 |
| UPF1 | 2.08 | 123 | 2 |
| ABCF1 | 1.74 | 91.6 | 3 |
| | 5.64 | 94.8 | 3 |
| KIF13B | 1.89 | 174.7 | 1 |
| WDR83 | 1.87 | 34.3 | 8 |
| SYNCRIP | 0 | 69.6 | 3 |
| ALDH9A1 | 0 | 53.8 | 4 |
| MSN | 3.82 | 67.8 | 3 |
| MFAP1 | 0 | 51.9 | 4 |
| SEC24D | 2.15 | 100.2 | 2 |
| ACIN1 | 1.99 | 151.8 | 1 |
| HSPD1 | 0 | 58.5 | 3 |
| PABPC4 | 0 | 72.3 | 3 |
| EIF3C | 2.17 | 103.6 | 2 |
| COPB1 | 3.86 | 107.1 | 2 |
| GFPT2 | 3.64 | 76.9 | 2 |
| POLR2A | 1.77 | 217 | 1 |
| CTNND1 | 0 | 104.8 | 2 |
| RPL34 | 1.61 | 13.3 | 15 |

| | | | |
|---|---|---|---|
| EIF3D | 3.45 | 63.9 | 4 |
| | 2.05 | 9.4 | 20 |
| COL12A1 | 0 | 332.9 | 1 |
| FAM120A | 0 | 121.8 | 2 |
| | 0 | 22.2 | 9 |
| ILF3 | 0 | 95.3 | 3 |
| RPS8 | 1.76 | 24.2 | 10 |
| | 0 | 84.6 | 2 |
| PSMC6 | 1.69 | 45.8 | 4 |
| IDH2 | 2.35 | 50.9 | 5 |
| RRBP1 | 0 | 84.3 | 3 |
| | 1.87 | 18.3 | 12 |
| GNB4 | 0 | 37.5 | 6 |
| KRT77 | 3.23 | 61.9 | 3 |
| | 1.9 | 25.3 | 8 |
| MYO1C | 1.72 | 121.6 | 2 |
| CAPRIN1 | 0 | 78.3 | 3 |
| ACIN1 | 1.99 | 84 | 2 |
| RPS25 | 1.78 | 13.7 | 16 |
| | 0 | 29.3 | 9 |
| FUS | 1.63 | 53.4 | 3 |
| NOP58 | 0 | 59.5 | 5 |
| SMARCA4 | 2.28 | 184.5 | 1 |
| ABCF2 | 2.05 | 72.4 | 3 |
| COPA | 3.81 | 138.3 | 1 |
| LASP1 | 1.77 | 29.7 | 6 |
| | 0 | 44.6 | 4 |
| | 1.92 | 99.9 | 2 |
| EIF4G1 | 1.75 | 158.5 | 1 |
| | 0 | 74.1 | 3 |
| TAF15 | 1.63 | 61.8 | 3 |
| CCT4 | 0 | 57.9 | 3 |
| RPL13 | 2.01 | 24.2 | 9 |
| ATAD3B | 1.78 | 72.5 | 3 |
| THBS2 | 0 | 129.9 | 2 |
| PRDX2 | 0 | 20.1 | 16 |
| | 1.87 | 81 | 2 |
| WWC3 | 0 | 65.5 | 3 |
| SNRNP70 | 3.83 | 51.5 | 4 |
| | 2.1 | 18.7 | 7 |
| FN1 | 0 | 259 | 1 |
| GATAD2B | 0 | 65.2 | 4 |
| PSMC2 | 0 | 48.6 | 6 |
| | 1.63 | 118.2 | 2 |
| RARS | 0 | 52.3 | 4 |
| SDHA | 1.68 | 72.6 | 3 |
| FMR1 | 0 | 63.9 | 3 |
| CSDA; YBX3 | 0 | 31.9 | 6 |

| | | | |
|---|---|---|---|
| HIST1H1B | 2.06 | 22.6 | 12 |
| POLR2C | 3.26 | 31.4 | 7 |
| LSM3 | 2.29 | 11.8 | 12 |
| KHSRP | 1.61 | 73.1 | 3 |
| EPRS | 0 | 97.5 | 2 |
| CAPZB | 1.75 | 30.6 | 6 |
| AP1M1 | 0 | 42.6 | 5 |
| RPS6 | 1.65 | 28.7 | 7 |
| SUPT5H | 2.6 | 120.9 | 1 |
| | 0 | 155.2 | 1 |
| UGGT2 | 1.94 | 174.6 | 1 |
| BUD13 | 0 | 70.5 | 3 |
| EIF3B | 0 | 85.1 | 2 |
| | 1.81 | 59.4 | 3 |
| | 0 | 37 | 6 |
| EIF5B | 1.95 | 138.7 | 1 |
| KLC1 | 0 | 68.7 | 3 |
| SLC25A6 | 0 | 32.8 | 6 |
| ALB | 0 | 69.3 | 4 |
| KPNB1 | 0 | 97.1 | 2 |
| SAP30BP | 1.95 | 33.9 | 5 |
| NUDT21 | 1.66 | 26.2 | 7 |
| LRP1 | 2.56 | 504.3 | 0 |
| RPS20 | 0 | 13.4 | 13 |
| | 2.04 | 7.1 | 25 |
| | 0 | 197 | 1 |
| PPIB | 2.42 | 22.7 | 6 |
| SON | 1.78 | 263.7 | 1 |
| DSP | 1.61 | 265 | 1 |
| AHNAK | 2.14 | 57.7 | 2 |
| RPL12 | 2.26 | 17.8 | 5 |
| GRB10 | 0 | 67.2 | 2 |
| SNRNP200 | 0 | 244.4 | 1 |
| | 2.18 | 20.2 | 5 |
| CCT2 | 0 | 57.5 | 2 |
| RUVBL1 | 0 | 34.8 | 4 |
| NXF1 | 2.07 | 15.8 | 7 |
| LUC7L3 | 1.9 | 51.4 | 4 |
| LTV1 | 2.27 | 54.8 | 2 |
| DSG1 | 0 | 113.7 | 1 |
| RBM39 | 2.11 | 56.4 | 2 |
| LLGL1 | 2 | 115.3 | 1 |
| RIOK1 | 1.67 | 65.5 | 2 |
| THBS1 | 2.51 | 129.3 | 1 |
| MAGED2 | 2.21 | 63.1 | 2 |
| RBBP7 | 0 | 46.9 | 3 |
| LMAN1 | 0 | 57.5 | 2 |
| TSR1 | 0 | 91.8 | 2 |

| | | | |
|---|---|---|---|
| C9orf114 | 3.97 | 42 | 3 |
| | 4.11 | 30.7 | 4 |
| GCFC1 | 1.73 | 104.7 | 1 |
| | 0 | 48.1 | 3 |
| RPN1 | 1.85 | 43.3 | 3 |
| | 0 | 102.5 | 1 |
| STRAP | 0 | 38.4 | 3 |
| RPL3 | 2.18 | 46.1 | 2 |
| ZNF326 | 0 | 65.6 | 2 |
| ZFC3H1 | 1.83 | 226.2 | 0 |
| PCNA | 2.29 | 28.7 | 3 |
| RPLP0 | 4.09 | 34.3 | 3 |
| SART1 | 1.65 | 27.5 | 4 |
| PACSIN2 | 0 | 50 | 2 |
| KIF2A | 0 | 75 | 2 |
| NAA15 | 1.67 | 101.2 | 1 |
| TOMM70A | 1.85 | 56.9 | 2 |
| SNRPD2 | 2.01 | 13.5 | 8 |
| MAPK1 | 2.35 | 41.4 | 3 |
| MCCC1 | 0 | 63.9 | 2 |
| MAGOH | 1.94 | 17.2 | 8 |
| | 0 | 18.7 | 7 |
| RPS7 | 1.93 | 22.1 | 4 |
| ALDH1L1 | 0 | 50.8 | 2 |
| STARD13 | 1.85 | 111.1 | 1 |
| BCAS2 | 1.73 | 26.1 | 5 |
| LARP1 | 0 | 123.4 | 1 |
| FAM98A | 0 | 20.7 | 5 |
| COL1A1 | 2.48 | 138.9 | 1 |
| | 1.7 | 15.3 | 7 |
| SEC63 | 0 | 37.6 | 3 |
| NKAPL | 1.81 | 46.3 | 2 |
| EIF2S1 | 1.76 | 36.1 | 3 |
| | 1.68 | 101.3 | 1 |
| LRRC47 | 0 | 63.4 | 2 |
| MAGT1 | 2.19 | 38 | 3 |
| RPS9 | 0 | 16.6 | 6 |
| RBM17 | 0 | 24.2 | 6 |
| | 0 | 34.2 | 3 |
| TAOK1 | 1.96 | 116 | 1 |
| DNTTIP2 | 0 | 84.4 | 1 |
| MCM5 | 1.63 | 82.2 | 1 |
| ATP5C1 | 0 | 32.2 | 3 |
| NUP93 | 0 | 93.3 | 1 |
| NUDC | 0 | 38.2 | 3 |
| | 0 | 36.4 | 3 |
| LUC7L | 0 | 43.7 | 3 |
| SEC22B | 1.65 | 24.6 | 5 |

| | | | |
|---|---|---|---|
| FRG1 | 1.69 | 29.2 | 3 |
| DHX8 | 1.68 | 133.9 | 1 |
| TFIP11 | 1.75 | 96.7 | 1 |
| RAE1 | 1.91 | 39.5 | 3 |
| | 0 | 17.5 | 7 |
| C11orf57 | 1.84 | 34.2 | 3 |
| YTHDF3 | 1.97 | 58.2 | 2 |
| COL11A2 | 0 | 171.7 | 1 |
| RPN2 | 3.71 | 31.3 | 3 |
| | 1.61 | 17.6 | 5 |
| HDLBP | 1.81 | 141.4 | 1 |
| | 1.73 | 20.4 | 7 |
| S100A4 | 2.08 | 11.7 | 9 |
| FNDC3B | 0 | 132.8 | 1 |
| | 1.65 | 14.3 | 8 |
| HELLS | 0 | 35.1 | 3 |
| NUP205 | 0 | 227.8 | 0 |
| PPIL1 | 1.93 | 18.2 | 5 |
| | 1.71 | 25.6 | 5 |
| | 0 | 11.3 | 9 |
| | 1.84 | 19 | 7 |
| CPSF1 | 1.63 | 160.8 | 1 |
| | 0 | 44.5 | 4 |
| | 0 | 13.8 | 6 |
| BGN | 0 | 33.5 | 3 |
| POR | 1.99 | 64.5 | 1 |
| SNRPF | 1.85 | 9.7 | 9 |
| SFSWAP | 0 | 104.8 | 1 |
| JPH2 | 0 | 45.1 | 2 |
| | 0 | 129.1 | 1 |
| | 2 | 30.1 | 4 |
| | 2.17 | 42.7 | 2 |
| EXO5 | 0 | 25.4 | 7 |
| ALDH18A1 | 0 | 87.2 | 1 |
| ERGIC1 | 0 | 32.6 | 3 |
| SRSF3 | 0 | 19.3 | 5 |
| SEC62 | 0 | 45.8 | 2 |
| NOTCH3 | 1.7 | 243.5 | 0 |
| NPM1 | 1.76 | 25 | 4 |
| PPIG | 0 | 27.5 | 4 |
| EIF3I | 0 | 36.5 | 3 |
| VPS35 | 1.61 | 91.6 | 1 |
| USP7 | 0 | 58.5 | 2 |
| NSUN2 | 0 | 86.4 | 1 |
| COL6A1 | 1.91 | 47.7 | 2 |
| ABCE1 | 0 | 67.3 | 2 |
| | 0 | 16.6 | 9 |
| UTRN | 1.8 | 394.2 | 0 |

| | | | |
|---|---|---|---|
| DAD1 | 1.64 | 12.4 | 10 |
| USP5 | 0 | 95.7 | 1 |
| | 1.62 | 54.7 | 2 |
| RPS6KA2 | 0 | 85.6 | 1 |
| | 0 | 35.5 | 3 |
| | 0 | 81.1 | 2 |
| PICALM | 1.62 | 70.7 | 1 |
| TMED9 | 1.98 | 25.1 | 4 |
| | 0 | 87.8 | 1 |
| GCN1L1 | 1.78 | 266.7 | 0 |
| SERPINH1 | 1.72 | 44.2 | 2 |
| GALK1 | 0 | 28.5 | 5 |
| HIST1H4A | 0 | 11.4 | 13 |
| PTRF | 0 | 39 | 3 |
| RPS11 | 0 | 18.4 | 5 |
| SAFB | 0 | 91 | 1 |
| | 1.98 | 30.8 | 3 |
| | 0 | 85.6 | 3 |
| | 1.73 | 21.4 | 4 |
| LRRC59 | 0 | 34.9 | 3 |
| U2AF2 | 0 | 53.1 | 2 |
| UCKL1 | 0 | 61.1 | 1 |
| ARPC4 | 0 | 19.7 | 7 |
| PAPSS2 | 0 | 70 | 2 |
| SARS | 1.62 | 61.3 | 1 |
| RPL37A | 0 | 10.3 | 9 |
| SNX18 | 0 | 68.9 | 2 |
| SNX6 | 0 | 24.6 | 5 |
| S100A6 | 1.81 | 10.2 | 9 |
| SMTN | 0 | 33.5 | 4 |
| | 1.67 | 8.2 | 11 |
| PHF6 | 0 | 41.3 | 2 |
| SPTBN1 | 0 | 274.2 | 0 |
| | 0 | 78 | 1 |
| NCAN | 0 | 143 | 1 |
| RBM14 | 0 | 69.4 | 2 |
| CKB | 0 | 44.9 | 4 |
| ASF1A | 1.98 | 23 | 5 |
| SRPR | 0 | 69.9 | 1 |
| | 0 | 143.1 | 1 |
| TM9SF4 | 0 | 69.8 | 1 |
| PSMC4 | 0 | 47.3 | 3 |
| MTHFD1 | 0 | 101.5 | 1 |
| ARGLU1 | 0 | 26.6 | 4 |
| KPNA4 | 2.06 | 57.9 | 1 |
| RPL19 | 0 | 20.8 | 5 |
| HIP1R | 0 | 119.3 | 1 |
| SEC24C | 0 | 106.5 | 1 |

| | | | |
|---|---|---|---|
| TJP2 | 1.82 | 117.7 | 1 |
| TARS | 0 | 70.3 | 2 |
| EHMT2 | 0 | 114.9 | 1 |
| EFHD2 | 2.05 | 26.7 | 4 |
| SMARCC1 | 0 | 122.8 | 1 |
| CTGF | 0 | 38 | 3 |
| ANXA7 | 0 | 50.3 | 2 |
| PPP2CB | 0 | 35.6 | 7 |
| RPL23 | 0 | 14.9 | 6 |
| CUL7 | 0 | 191 | 1 |
| HIST2H2BE | 0 | 13.9 | 7 |
| ILF2 | 1.66 | 43 | 2 |
| WBP1L | 0 | 16.5 | 15 |
| PA2G4 | 0 | 40.9 | 2 |
| ASL | 0 | 39.8 | 2 |
| | 0 | 14.7 | 11 |
| LAS1L | 0 | 21.9 | 4 |
| RPL4 | 0 | 47.7 | 2 |
| RRAS2 | 0 | 23.4 | 5 |
| RAC2 | 0 | 18.5 | 7 |
| GALE | 1.95 | 26.2 | 3 |
| | 0 | 48.2 | 2 |
| PDIA3 | 0 | 54.1 | 3 |
| | 0 | 54.6 | 2 |
| | 0 | 19.7 | 5 |
| CRTAP | 0 | 29.8 | 3 |
| CCT6A | 0 | 57.9 | 2 |
| | 0 | 82.6 | 2 |
| PAPSS1 | 0 | 70.8 | 2 |
| TRAP1 | 0 | 80 | 2 |
| SPATS2 | 0 | 59.5 | 2 |
| MYOF | 0 | 234.6 | 1 |
| | 0 | 49.3 | 2 |
| IGF2R | 2.47 | 274.2 | 0 |
| RAP2B | 0 | 20.3 | 6 |
| | 0 | 61.9 | 1 |
| SNRPA | 0 | 31.3 | 3 |
| | 0 | 25.1 | 4 |
| WDR5 | 0 | 36.6 | 2 |
| BMS1 | 0 | 145.7 | 1 |
| KARS | 1.62 | 48.4 | 2 |
| FASN | 0 | 273.3 | 0 |
| CCDC9 | 0 | 58 | 2 |
| FAM120C | 0 | 99.3 | 1 |
| USP10 | 1.77 | 87.1 | 1 |
| ARL1 | 0 | 15.4 | 7 |
| | 1.78 | 51.1 | 2 |
| CORO1C | 0 | 41.6 | 3 |

| | | | |
|---|---|---|---|
| AIMP1 | 0 | 34.3 | 3 |
| | 0 | 46.5 | 3 |
| NAA11 | 0 | 26 | 3 |
| RBM26 | 0 | 113.5 | 1 |
| | 0 | 16.9 | 7 |
| MST4 | 0 | 43.8 | 2 |
| SCFD1 | 0 | 51.5 | 2 |
| | 1.6 | 19.2 | 5 |
| EMC8 | 0 | 23.8 | 4 |
| CHST11 | 1.61 | 41.5 | 2 |
| | 0 | 31.1 | 3 |
| PSMA5 | 0 | 26.4 | 4 |
| | 0 | 21.1 | 8 |
| RPS12 | 0 | 14.5 | 8 |
| | 0 | 13.4 | 10 |
| SUPT6H | 0 | 198.9 | 0 |
| | 0 | 28.2 | 4 |
| HK1 | 1.76 | 105.7 | 1 |
| RPL7A | 0 | 15.6 | 7 |
| PPIL4 | 0 | 57.2 | 2 |
| SRP72 | 1.66 | 74.6 | 1 |
| NCOA5 | 1.63 | 13.1 | 10 |
| G3BP1 | 0 | 52.1 | 3 |
| CTBP2 | 0 | 48.9 | 2 |
| EIF2B3 | 0 | 50.2 | 2 |
| SRPRB | 0 | 29.7 | 7 |
| KANK4 | 0 | 107.3 | 1 |
| | 0 | 68.3 | 3 |
| HGS | 0 | 74.8 | 1 |
| CAV1 | 0 | 25.1 | 4 |
| FXR1 | 1.68 | 68.3 | 1 |
| LSG1 | 0 | 75.2 | 1 |
| | 0 | 23 | 5 |
| KIF3B | 1.73 | 85.1 | 1 |
| SNRNP40 | 0 | 39.3 | 3 |
| TAOK2 | 0 | 93.9 | 1 |
| SLC25A3 | 0 | 39.9 | 2 |
| PRKRA | 0 | 22.1 | 5 |
| ZDHHC5 | 0 | 77.5 | 1 |
| SEC23A | 0 | 82.9 | 1 |
| NSF | 0 | 72.2 | 2 |
| AZGP1 | 0 | 34.2 | 3 |
| IKBKAP | 0 | 150.1 | 1 |
| ZFR | 0 | 116.9 | 1 |
| PSMD1 | 0 | 105.8 | 1 |
| RSRC2 | 1.96 | 22.5 | 3 |
| TCEB2 | 0 | 13.1 | 8 |
| PPP2R1A | 0 | 65.3 | 1 |

| | | | |
|---|---|---|---|
| MATN1 | 0 | 53.6 | 2 |
| BCCIP | 0 | 29.8 | 3 |
| | 2.05 | 14.8 | 6 |
| PARD3 | 0 | 144.4 | 1 |
| | 2.32 | 25.6 | 3 |
| PATL1 | 0 | 37.7 | 3 |
| IARS | 0 | 87.7 | 1 |
| | 0 | 40 | 3 |
| CHPF2 | 1.77 | 85.9 | 1 |
| GAA | 0 | 105.3 | 1 |
| | 0 | 8 | 13 |
| DMPK | 0 | 69 | 1 |
| LDHA | 1.66 | 33.6 | 3 |
| SF3A2 | 0 | 51.4 | 3 |
| SLU7 | 0 | 68.3 | 2 |
| SEC16A | 0 | 115.2 | 1 |
| PPIAL4C | 0 | 18.1 | 4 |
| TPI1 | 0 | 22.9 | 6 |
| | 1.77 | 7.2 | 11 |
| | 0 | 108.5 | 1 |
| SF3B4 | 0 | 44.5 | 3 |
| DRG1 | 0 | 40.5 | 4 |
| PSMD4 | 1.94 | 21.8 | 4 |
| HADHB | 0 | 49.6 | 2 |
| ARHGEF7 | 0 | 81.4 | 1 |
| SRP68 | 1.62 | 41.3 | 2 |
| | 1.79 | 19.4 | 4 |
| DDX39A | 0 | 53.7 | 2 |
| EIF5AL1 | 0 | 16.8 | 5 |
| PDS5B | 0 | 164.6 | 1 |
| EDC4 | 0 | 151.6 | 1 |
| DST | 0 | 847.4 | 0 |
| | 0 | 44 | 2 |
| ATP5A1 | 0 | 59.7 | 2 |

**Supplementary Table 3. Identifications of proteins pulled down by the circKIF18A probe.**

| Gene Symbol | Protein_score | MW [kDa] | Coverage [%] |
|---|---|---|---|
| MYH9 | 594.39 | 226.4 | 66 |
| MYH10 | 229.57 | 228.9 | 47 |
| PLEC | 191.39 | 531.5 | 26 |
| MCM7 | 150.66 | 81.3 | 60 |
| ACTB | 146.45 | 41.7 | 57 |
| DYNC1H1 | 94.17 | 532.1 | 15 |
| FLNA | 92.59 | 280.6 | 27 |
| PRKDC | 76.98 | 468.8 | 16 |
| ACTA2 | 67.61 | 37.3 | 37 |
| | 67.41 | 14.5 | 88 |
| ACTN4 | 66.95 | 104.8 | 43 |
| TPM1 | 66.65 | 32.7 | 61 |
| | 58.63 | 16.3 | 67 |
| MYO5A | 56.55 | 215.3 | 24 |
| TPM1 | 56.22 | 28.7 | 60 |
| HNRNPM | 54.44 | 77.5 | 40 |
| MYO1C | 54.4 | 121.6 | 27 |
| RBM25 | 52.58 | 100.1 | 30 |
| FN1 | 52.11 | 259 | 17 |
| ACTBL2 | 51.6 | 42 | 21 |
| MYO18A | 51.42 | 233 | 21 |
| TPM1 | 50.74 | 32.7 | 52 |
| COL6A3 | 50.36 | 343.5 | 12 |
| FASN | 49.79 | 273.3 | 18 |
| ACACA | 48.91 | 257.1 | 18 |
| CLTC | 48.15 | 191.9 | 22 |
| HSPA8 | 47.24 | 70.9 | 33 |
| IQGAP1 | 47.02 | 189.2 | 19 |
| TPM4 | 46.43 | 28.5 | 72 |
| PCCA | 45.64 | 80 | 39 |
| EEF2 | 44.79 | 95.3 | 29 |
| ACTG1 | 44.49 | 25 | 46 |
| | 44.41 | 28.7 | 74 |
| KRT1 | 43.52 | 66 | 32 |
| ATP1A1 | 43.28 | 112.8 | 26 |
| TPM3 | 43.14 | 33.2 | 62 |
| PCCB | 42.79 | 58.2 | 34 |
| HSP90AB1 | 42.3 | 83.2 | 32 |
| FLNB | 42.03 | 278 | 18 |
| ACTN1 | 41.02 | 103 | 31 |
| STAT1 | 39.59 | 87.3 | 31 |
| SVIL | 38.36 | 247.6 | 14 |
| TPM3 | 38.32 | 29 | 69 |
| TUBB | 37.9 | 49.6 | 26 |
| MYO1C | 37.13 | 92.3 | 23 |
| CAPZB | 36.93 | 30.6 | 35 |

| | | | |
|---|---|---|---|
| LIMA1 | 36.87 | 85.2 | 36 |
| COPA | 36.56 | 138.3 | 20 |
| FLII | 36.41 | 144.7 | 16 |
| | 36.06 | 74.1 | 33 |
| ATP2A2 | 35.69 | 114.7 | 19 |
| | 35.26 | 40 | 40 |
| TPM2 | 35.22 | 33 | 41 |
| VIM | 35.18 | 53.6 | 48 |
| TUBB6 | 35.14 | 49.8 | 22 |
| TUBB3 | 34.92 | 50.4 | 26 |
| PRPF8 | 34.66 | 273.4 | 13 |
| MYO1B | 33.27 | 128.4 | 22 |
| DDX41 | 33.05 | 69.8 | 36 |
| MYH14 | 32.72 | 227.7 | 8 |
| TPM2 | 32.68 | 32.8 | 40 |
| MYO6 | 32.56 | 146.9 | 26 |
| KRT2 | 32.2 | 65.4 | 33 |
| DDX21 | 31.82 | 87.3 | 27 |
| KRT9 | 31.82 | 62 | 31 |
| ANXA2 | 31.32 | 38.6 | 37 |
| | 30.87 | 19.8 | 40 |
| DHX9 | 30.84 | 140.9 | 17 |
| MYL12B | 30.72 | 19.8 | 41 |
| HSPA5 | 30.59 | 72.3 | 30 |
| GSN | 30.18 | 78.8 | 32 |
| | 30 | 67 | 33 |
| TLN1 | 29.8 | 269.6 | 10 |
| POTEF | 29.43 | 121.4 | 7 |
| CKAP4 | 28.36 | 66 | 38 |
| SNRNP200 | 28.12 | 216 | 14 |
| PC | 27.65 | 129.6 | 18 |
| NCL | 26.93 | 76.6 | 24 |
| SMARCA5 | 26.74 | 121.8 | 21 |
| HNRNPUL1 | 26.56 | 95.7 | 16 |
| SMC3 | 26.42 | 141.4 | 18 |
| MCCC2 | 26.3 | 61.3 | 32 |
| MYO5B | 26.14 | 213.5 | 11 |
| RPS8 | 25.7 | 24.2 | 41 |
| EIF4A1 | 25.69 | 46.1 | 31 |
| | 25.61 | 99.9 | 25 |
| | 25.34 | 76.3 | 25 |
| | 25.33 | 216.7 | 15 |
| RPL4 | 24.92 | 47.7 | 33 |
| TCP1 | 24.82 | 60.3 | 21 |
| HNRNPU | 24.76 | 90.5 | 19 |
| PABPC1 | 24.76 | 47.3 | 35 |
| KIF5B | 24.64 | 109.6 | 24 |
| DDX3X | 24.53 | 73.2 | 22 |

| | | | |
|---|---|---|---|
| DDX1 | 24.23 | 82.4 | 23 |
| SERPINH1 | 24.15 | 44.2 | 25 |
| ABCF1 | 24.09 | 95.9 | 17 |
| LCP1 | 23.98 | 70.2 | 35 |
| MTHFD1 | 23.82 | 101.5 | 21 |
| SRRM2 | 23.78 | 299.4 | 9 |
| EPRS | 23.56 | 170.5 | 18 |
| ERLIN2 | 23.31 | 37.8 | 28 |
| | 23.18 | 85.6 | 18 |
| SMCHD1 | 23.07 | 226.2 | 9 |
| HNRNPK | 22.94 | 48.5 | 25 |
| HSPA9 | 22.49 | 72.4 | 27 |
| HSP90AA1 | 22.45 | 84.6 | 23 |
| KRT10 | 22.44 | 58.8 | 18 |
| NOP56 | 22.36 | 66 | 39 |
| | 21.92 | 84.6 | 15 |
| SND1 | 21.81 | 99.6 | 17 |
| TUBA4A | 21.79 | 49.9 | 24 |
| GART | 21.74 | 107.7 | 10 |
| MCCC1 | 21.68 | 80.4 | 18 |
| RPL15 | 21.66 | 24.1 | 40 |
| RARS | 21.61 | 75.3 | 17 |
| VARS | 21.6 | 140.4 | 15 |
| ILF3 | 21.16 | 76.4 | 27 |
| DDX17 | 21.15 | 80.2 | 16 |
| MYO1D | 20.94 | 111.2 | 13 |
| RPN1 | 20.93 | 68.5 | 18 |
| MYO1E | 20.89 | 127 | 19 |
| MSH6 | 20.54 | 121 | 9 |
| PABPC4 | 20.47 | 72.3 | 16 |
| IMMT | 20.46 | 73.3 | 15 |
| ATAD3A | 20.36 | 71.3 | 20 |
| SF3B1 | 20.35 | 145.7 | 13 |
| LMNB1 | 20.28 | 66.4 | 19 |
| TMOD3 | 20.13 | 39.5 | 45 |
| TOP2B | 20.11 | 183.2 | 9 |
| DCTN1 | 20.05 | 141.6 | 12 |
| MYBBP1A | 20.03 | 148.8 | 10 |
| SPTBN1 | 20.01 | 274.4 | 11 |
| | 19.99 | 52.5 | 22 |
| P4HA2 | 19.97 | 60.6 | 22 |
| CSDA | 19.96 | 31.9 | 45 |
| | 19.93 | 54.6 | 32 |
| GCN1L1 | 19.92 | 292.6 | 9 |
| ATAD3B | 19.89 | 65.1 | 19 |
| RSL1D1 | 19.79 | 54.9 | 35 |
| ATP5B | 19.77 | 48.1 | 29 |
| | 19.77 | 64 | 16 |

| | | | |
|---|---|---|---|
| | 19.77 | 59.2 | 19 |
| PSMC2 | 19.73 | 48.6 | 34 |
| MYL6B | 19.71 | 24.5 | 50 |
| MYOF | 19.7 | 234.6 | 6 |
| HSPD1 | 19.57 | 60.6 | 19 |
| XRCC6 | 19.56 | 64.2 | 20 |
| SUPT16H | 19.55 | 119.8 | 15 |
| LRRFIP2 | 19.36 | 84.1 | 14 |
| RPS6 | 19.15 | 28.7 | 29 |
| | 19.09 | 25.1 | 30 |
| KRT5 | 19.01 | 62.3 | 18 |
| EIF5B | 18.92 | 138.7 | 10 |
| DHX15 | 18.81 | 90.9 | 23 |
| EIF4A3 | 18.81 | 46.8 | 33 |
| SAMHD1 | 18.67 | 72.2 | 21 |
| SFPQ | 18.64 | 68.6 | 11 |
| | 18.54 | 143.1 | 20 |
| UPF1 | 18.5 | 123 | 17 |
| | 18.47 | 152.4 | 13 |
| KRT16 | 18.43 | 51.2 | 19 |
| DDX5 | 18.4 | 69.1 | 21 |
| RPS3A | 18.27 | 29.9 | 47 |
| RPL3 | 18.21 | 46.1 | 35 |
| MCM6 | 17.87 | 88.9 | 14 |
| TMOD1 | 17.86 | 40.5 | 18 |
| CDC42BPB | 17.8 | 194.2 | 10 |
| P4HA1 | 17.77 | 61 | 23 |
| SART3 | 17.67 | 109.9 | 18 |
| TOP2A | 17.66 | 174.3 | 10 |
| AP3D1 | 17.64 | 130.1 | 13 |
| ABCD3 | 17.58 | 78.4 | 13 |
| | 17.33 | 52.2 | 22 |
| IDH2 | 17.3 | 50.9 | 20 |
| CCT5 | 17.21 | 59.4 | 21 |
| GNB2L1 | 17.09 | 35.1 | 25 |
| PFKL | 17.04 | 85 | 14 |
| CAPN1 | 16.93 | 81.8 | 15 |
| GARS | 16.92 | 77.5 | 15 |
| | 16.9 | 25.3 | 30 |
| RPL13 | 16.89 | 24.2 | 29 |
| MAP4K4 | 16.83 | 145.8 | 12 |
| | 16.78 | 49.8 | 30 |
| PALLD | 16.68 | 122 | 10 |
| NUDC | 16.56 | 38.2 | 26 |
| CORO1C | 16.47 | 53.2 | 22 |
| CAP1 | 16.45 | 47.4 | 28 |
| RAD50 | 16.39 | 153.8 | 11 |
| | 16.34 | 142.2 | 13 |

| | | | |
|---|---|---|---|
| ABCE1 | 16.33 | 67.3 | 22 |
| | 16.31 | 48.1 | 22 |
| RPL3 | 16.29 | 40.2 | 34 |
| PARP1 | 16.19 | 113 | 16 |
| RUVBL2 | 16.14 | 51.1 | 21 |
| RPS27A | 16.12 | 18 | 55 |
| EFHD2 | 16.07 | 26.7 | 37 |
| RAN | 15.87 | 26.2 | 37 |
| HNRNPR | 15.85 | 70.9 | 22 |
| | 15.65 | 49.9 | 24 |
| KRT14 | 15.62 | 51.5 | 24 |
| NOP58 | 15.51 | 59.5 | 14 |
| PRPF6 | 15.5 | 106.9 | 10 |
| DARS | 15.49 | 57.1 | 22 |
| HNRNPC | 15.43 | 32 | 36 |
| ESYT1 | 15.42 | 122.8 | 10 |
| | 15.41 | 50.6 | 21 |
| | 15.39 | 21.5 | 49 |
| RPL19 | 15.39 | 23.5 | 31 |
| ATP5A1 | 15.33 | 59.7 | 23 |
| AFG3L2 | 15.33 | 88.5 | 16 |
| CCT8 | 15.32 | 59.6 | 23 |
| | 15.21 | 57.2 | 15 |
| NAT10 | 15.13 | 115.7 | 12 |
| PFKP | 15 | 85.5 | 20 |
| ATP5C1 | 14.93 | 32.2 | 26 |
| ACIN1 | 14.92 | 151.8 | 9 |
| KRT75 | 14.92 | 59.5 | 17 |
| PHGDH | 14.92 | 56.6 | 17 |
| DST | 14.86 | 847.4 | 2 |
| YBX1 | 14.81 | 29.4 | 53 |
| PSMD3 | 14.77 | 58.5 | 23 |
| EIF2S1 | 14.73 | 36.1 | 26 |
| NDUFS1 | 14.73 | 79.4 | 13 |
| EIF2S3 | 14.59 | 51.1 | 25 |
| LRPPRC | 14.56 | 157.8 | 9 |
| CYFIP1 | 14.55 | 145.1 | 11 |
| HNRNPA0 | 14.51 | 30.8 | 23 |
| VCL | 14.5 | 116.6 | 9 |
| | 14.49 | 100.7 | 16 |
| NUP93 | 14.45 | 93.3 | 16 |
| RPL13AP25 | 14.45 | 23.6 | 31 |
| HNRNPA2B1 | 14.44 | 37.4 | 32 |
| NONO | 14.41 | 54.3 | 20 |
| CCT3 | 14.37 | 63.5 | 16 |
| CEBPZ | 14.34 | 120.9 | 11 |
| CEBPZ | 14.3 | 114.1 | 11 |
| SAP30BP | 14.29 | 33.9 | 19 |

| | | | |
|---|---|---|---|
| PHB | 14.23 | 29.8 | 37 |
| | 14.21 | 129.1 | 11 |
| SEC31A | 14.19 | 129 | 10 |
| EIF3E | 14.07 | 52.2 | 15 |
| RPL23A | 14.06 | 17.7 | 41 |
| MCM2 | 14 | 106.8 | 11 |
| EIF3A | 13.98 | 166.4 | 9 |
| UBR4 | 13.93 | 573.5 | 3 |
| DTX3L | 13.89 | 83.5 | 22 |
| ADAR | 13.77 | 137.7 | 6 |
| SLC25A13 | 13.76 | 74.1 | 13 |
| CACYBP | 13.75 | 26.2 | 40 |
| EIF3C | 13.69 | 103.6 | 11 |
| HADHA | 13.68 | 78.3 | 12 |
| | 13.63 | 99.7 | 10 |
| BUB3 | 13.63 | 37.1 | 31 |
| HNRNPH1 | 13.61 | 47.1 | 31 |
| ENO1 | 13.61 | 47.1 | 19 |
| CAND1 | 13.58 | 136.3 | 8 |
| GTF2I | 13.57 | 110.2 | 12 |
| SNRNP70 | 13.46 | 51.5 | 23 |
| | 13.4 | 49.8 | 18 |
| NSF | 13.4 | 77.8 | 12 |
| GAPDH | 13.39 | 36 | 38 |
| ATP13A1 | 13.32 | 121 | 9 |
| DDX50 | 13.21 | 82.5 | 14 |
| PNN | 13.19 | 81.5 | 12 |
| | 13.17 | 111.9 | 14 |
| PKM | 13.15 | 58 | 20 |
| PDCD11 | 13.14 | 208.6 | 10 |
| EEF1A1 | 13.1 | 50.2 | 16 |
| FHL2 | 13 | 32.2 | 35 |
| HDLBP | 12.69 | 141.4 | 9 |
| HSP90B1 | 12.55 | 92.4 | 16 |
| VDAC1 | 12.54 | 30.8 | 24 |
| RPL7A | 12.53 | 30 | 35 |
| RPS3 | 12.52 | 26.7 | 53 |
| COPB1 | 12.48 | 107.1 | 14 |
| PML | 12.45 | 97.5 | 10 |
| RPL10A | 12.39 | 24.8 | 39 |
| TRIM28 | 12.33 | 88.5 | 9 |
| AIFM1 | 12.33 | 66.9 | 15 |
| | 12.31 | 81.1 | 11 |
| RNF213 | 12.24 | 596.1 | 3 |
| TAP2 | 12.16 | 75.7 | 6 |
| CDC5L | 12.11 | 92.2 | 14 |
| FIP1L1 | 12.09 | 66.5 | 8 |
| RPS18 | 12.08 | 17.7 | 39 |

| | | | |
|---|---|---|---|
| SERBP1 | 12.06 | 44.9 | 26 |
| LMNB2 | 12.03 | 69.9 | 18 |
| TARS | 12.02 | 82.1 | 13 |
| HP1BP3 | 12 | 61.2 | 15 |
| CAPZA2 | 11.95 | 32.9 | 26 |
| DDX23 | 11.93 | 95.5 | 16 |
| DNAJA1 | 11.88 | 44.8 | 18 |
| SGPL1 | 11.84 | 63.5 | 18 |
| MRPS5 | 11.8 | 48 | 20 |
| CSDE1 | 11.78 | 88.8 | 14 |
| CAPN2 | 11.76 | 60.5 | 16 |
| MARS | 11.71 | 101.1 | 9 |
| ASPH | 11.7 | 85.8 | 16 |
| STT3A | 11.69 | 80.5 | 12 |
| CCT2 | 11.61 | 57.5 | 16 |
| PSMC6 | 11.57 | 45.8 | 18 |
| COL5A1 | 11.47 | 183.5 | 4 |
| | 11.41 | 14.7 | 49 |
| SLC25A3 | 11.33 | 39.9 | 18 |
| AP2A1 | 11.31 | 107.5 | 10 |
| PLS3 | 11.24 | 70.7 | 18 |
| | 11.24 | 116 | 10 |
| DDX27 | 11.22 | 89.8 | 14 |
| H1F0 | 11.22 | 20.9 | 18 |
| RPS2 | 11.15 | 31.3 | 19 |
| AARS | 11.12 | 106.7 | 8 |
| MSH2 | 11.12 | 104.7 | 7 |
| LUC7L2 | 11.09 | 46.5 | 30 |
| XRCC5 | 11.01 | 82.7 | 16 |
| KRT6B | 10.98 | 60 | 16 |
| DHX30 | 10.83 | 130.5 | 10 |
| TOP1 | 10.81 | 90.6 | 17 |
| | 10.81 | 29.3 | 20 |
| COL1A1 | 10.78 | 138.9 | 6 |
| | 10.75 | 18.6 | 31 |
| DNAJC13 | 10.73 | 254.3 | 5 |
| DPYSL3 | 10.67 | 73.9 | 22 |
| EHD4 | 10.67 | 61.1 | 16 |
| AP3B1 | 10.64 | 121.2 | 14 |
| RANBP2 | 10.61 | 358 | 4 |
| KARS | 10.6 | 68 | 19 |
| GFPT1 | 10.57 | 78.8 | 18 |
| FLNC | 10.55 | 290.8 | 4 |
| UGGT1 | 10.55 | 174.9 | 4 |
| SRSF1 | 10.54 | 25.6 | 22 |
| COL1A1 | 10.51 | 84.7 | 10 |
| PTRF | 10.43 | 43.5 | 20 |
| | 10.41 | 70.1 | 15 |

| | | | |
|---|---|---|---|
| PLAA | 10.3 | 87.1 | 13 |
| LLGL1 | 10.27 | 115.3 | 6 |
| | 10.23 | 282.1 | 4 |
| LGALS3BP | 10.18 | 54.1 | 9 |
| FAM120A | 10.16 | 121.8 | 10 |
| PRPF40A | 10.11 | 108.7 | 9 |
| ABCF2 | 10.1 | 72.4 | 18 |
| HIST1H1C | 10.09 | 21.4 | 28 |
| SYNCRIP | 10.08 | 69.6 | 15 |
| | 10.07 | 19 | 22 |
| IGF2BP2 | 10.04 | 66.1 | 17 |
| FUBP3 | 10.01 | 61.6 | 12 |
| STK10 | 9.98 | 112.1 | 6 |
| AIMP1 | 9.96 | 34.3 | 21 |
| | 9.96 | 29.7 | 23 |
| LRRFIP1 | 9.95 | 44.9 | 17 |
| YWHAQ | 9.95 | 23.8 | 28 |
| COPB2 | 9.89 | 102.4 | 20 |
| | 9.81 | 51 | 25 |
| PDHA1 | 9.78 | 43.2 | 23 |
| ACTR3 | 9.77 | 40.7 | 15 |
| WARS | 9.76 | 53.1 | 17 |
| | 9.73 | 102.5 | 7 |
| SON | 9.72 | 263.7 | 5 |
| IPO9 | 9.67 | 115.9 | 6 |
| MYL1 | 9.67 | 15.6 | 39 |
| SMARCA4 | 9.66 | 184.5 | 9 |
| ASCC3 | 9.62 | 251.3 | 6 |
| DKC1 | 9.62 | 57.6 | 18 |
| THRAP3 | 9.61 | 108.6 | 14 |
| PSMD4 | 9.6 | 40.7 | 16 |
| UBA1 | 9.52 | 117.8 | 7 |
| | 9.5 | 39.1 | 12 |
| IQGAP2 | 9.46 | 132.6 | 5 |
| DDX18 | 9.42 | 75.4 | 12 |
| COPG1 | 9.41 | 97.7 | 13 |
| PCNA | 9.38 | 28.7 | 24 |
| DPYSL2 | 9.36 | 68.1 | 17 |
| MSN | 9.33 | 67.8 | 20 |
| C22orf28 | 9.27 | 55.2 | 21 |
| RPN2 | 9.24 | 69.2 | 7 |
| AP2B1 | 9.23 | 105.6 | 9 |
| HK1 | 9.21 | 105.7 | 9 |
| LUC7L | 9.15 | 43.7 | 30 |
| PSMD6 | 9.15 | 45.5 | 12 |
| ACSL3 | 9.14 | 80.3 | 9 |
| | 9.14 | 16.6 | 23 |
| LMO7 | 9.13 | 192.6 | 5 |

| | | | |
|---|---|---|---|
| SMC4 | 9.11 | 147.1 | 11 |
| RPS12 | 9.11 | 14.5 | 47 |
| SPTLC1 | 9.1 | 52.7 | 15 |
| SMC2 | 9.06 | 135.5 | 12 |
| SLK | 9.05 | 142.6 | 5 |
| FXR1 | 9 | 67.2 | 23 |
| | 9 | 31.7 | 11 |
| RPL7 | 8.96 | 29.2 | 24 |
| PDLIM4 | 8.96 | 35.4 | 27 |
| | 8.95 | 98.1 | 9 |
| | 8.92 | 81 | 8 |
| LBR | 8.92 | 70.7 | 8 |
| SRSF2 | 8.89 | 25.5 | 19 |
| RPL11 | 8.88 | 20.2 | 22 |
| WDR11 | 8.88 | 136.7 | 3 |
| ALDOA | 8.86 | 39.4 | 13 |
| PPP1CB | 8.84 | 37.2 | 20 |
| | 8.84 | 33.1 | 21 |
| KHDRBS1 | 8.81 | 48.2 | 15 |
| SRP68 | 8.75 | 70.7 | 12 |
| | 8.75 | 59.2 | 12 |
| UQCRC1 | 8.75 | 40.3 | 9 |
| LRRC47 | 8.71 | 63.4 | 14 |
| RPL35 | 8.71 | 14.5 | 34 |
| ILF2 | 8.7 | 43 | 11 |
| USO1 | 8.66 | 107.8 | 8 |
| USP7 | 8.65 | 128.2 | 6 |
| VDAC2 | 8.63 | 34.5 | 21 |
| ARCN1 | 8.61 | 57.2 | 25 |
| RPL27A | 8.58 | 12 | 29 |
| HYOU1 | 8.57 | 109.6 | 9 |
| SLC25A5 | 8.55 | 32.8 | 31 |
| CDK11A | 8.54 | 91.3 | 8 |
| OAT | 8.54 | 48.5 | 11 |
| PRPF4B | 8.53 | 116.9 | 5 |
| DDX39A | 8.52 | 53.7 | 10 |
| OAS3 | 8.51 | 121.1 | 9 |
| MYLK | 8.5 | 210.6 | 6 |
| C9orf114 | 8.48 | 42 | 14 |
| | 8.45 | 187.7 | 6 |
| | 8.42 | 101.4 | 4 |
| HNRNPH2 | 8.4 | 49.2 | 23 |
| RBBP4 | 8.4 | 47.6 | 15 |
| RCC2 | 8.35 | 56 | 11 |
| KPNA6 | 8.35 | 60 | 12 |
| PYGB | 8.34 | 96.6 | 17 |
| EIF3B | 8.3 | 85.1 | 16 |
| KRT17 | 8.24 | 48.1 | 14 |

| | | | |
|---|---|---|---|
| ITPR3 | 8.22 | 303.9 | 2 |
| SRPK2 | 8.2 | 77.5 | 7 |
| NUP210 | 8.19 | 205 | 6 |
| UHRF1 | 8.19 | 89.8 | 8 |
| GTPBP4 | 8.18 | 73.9 | 13 |
| | 8.13 | 44.6 | 12 |
| CGGBP1 | 8.13 | 18.8 | 31 |
| EIF2S2 | 8.12 | 38.3 | 23 |
| | 8.11 | 22.4 | 19 |
| SFXN3 | 8.08 | 36 | 28 |
| NSUN2 | 8.08 | 86.4 | 10 |
| SAP18 | 8.02 | 17.6 | 35 |
| | 8 | 27.5 | 23 |
| HNRNPF | 7.96 | 45.6 | 13 |
| DDX46 | 7.95 | 117.4 | 8 |
| STAT2 | 7.95 | 97.9 | 9 |
| RPL36AL | 7.95 | 12.5 | 17 |
| BOP1 | 7.89 | 83.5 | 10 |
| AHSA1 | 7.89 | 38.3 | 22 |
| SNX6 | 7.85 | 46.6 | 20 |
| ERAP1 | 7.85 | 107.1 | 4 |
| QARS | 7.83 | 82.2 | 15 |
| CORO2B | 7.82 | 54.9 | 15 |
| HIST1H1E | 7.82 | 21.9 | 23 |
| PSMD1 | 7.76 | 105.8 | 14 |
| EFTUD2 | 7.73 | 108.1 | 11 |
| HSD17B4 | 7.72 | 79.6 | 13 |
| DIS3 | 7.72 | 108.9 | 7 |
| U2AF2 | 7.71 | 53.1 | 11 |
| SLC25A6 | 7.68 | 32.8 | 36 |
| EBNA1BP2 | 7.66 | 34.8 | 16 |
| CDK9 | 7.66 | 42.7 | 13 |
| MRPS15 | 7.66 | 29.8 | 18 |
| TECR | 7.65 | 36 | 15 |
| HNRNPDL | 7.64 | 30.2 | 12 |
| SUGP2 | 7.59 | 120.2 | 6 |
| NDUFA10 | 7.59 | 40.7 | 14 |
| | 7.57 | 856.3 | 2 |
| CALD1 | 7.57 | 57.4 | 17 |
| ALDH2 | 7.55 | 56.3 | 8 |
| PGAM5 | 7.55 | 32 | 8 |
| ACLY | 7.54 | 120.8 | 9 |
| KRT8 | 7.52 | 53.7 | 18 |
| RPLP0 | 7.52 | 34.3 | 14 |
| SCAF11 | 7.5 | 164.6 | 6 |
| NCLN | 7.45 | 61.7 | 8 |
| KIF2A | 7.44 | 75 | 18 |
| | 7.44 | 25.6 | 24 |

| | | | |
|---|---|---|---|
| PARP9 | 7.41 | 87.5 | 8 |
| | 7.39 | 21.2 | 15 |
| SPATS2L | 7.32 | 61.7 | 17 |
| ALDH3A2 | 7.29 | 54.8 | 8 |
| EIF3I | 7.29 | 36.5 | 15 |
| DCTN4 | 7.25 | 52.3 | 20 |
| RAB18 | 7.25 | 23 | 28 |
| RPL28 | 7.24 | 15.7 | 39 |
| KIFC1 | 7.23 | 73 | 8 |
| TBL1XR1 | 7.19 | 40.7 | 18 |
| EIF3L | 7.16 | 66.7 | 12 |
| RAB1A | 7.16 | 22.7 | 20 |
| HECTD1 | 7.15 | 289.2 | 4 |
| DHRS2 | 7.13 | 29.9 | 23 |
| MAP7D1 | 7.12 | 64.6 | 7 |
| CRTAP | 7.11 | 45 | 22 |
| IFI16 | 7.09 | 88.2 | 20 |
| UGDH | 7.09 | 55 | 13 |
| KHSRP | 7.08 | 73.1 | 9 |
| CDK1 | 7.07 | 34.1 | 19 |
| CAAP1 | 7.07 | 38.3 | 10 |
| | 7.04 | 74.6 | 12 |
| NOP2 | 7.01 | 89.2 | 10 |
| FMNL3 | 7.01 | 117.1 | 6 |
| CHD5 | 6.99 | 222.9 | 3 |
| RPS25 | 6.97 | 13.7 | 20 |
| CHERP | 6.96 | 103.6 | 8 |
| | 6.96 | 139.5 | 5 |
| TBL3 | 6.95 | 89 | 8 |
| RPS23 | 6.94 | 15.8 | 16 |
| SFXN1 | 6.91 | 35.6 | 12 |
| STAU1 | 6.89 | 63.1 | 23 |
| SSRP1 | 6.88 | 81 | 7 |
| | 6.88 | 8 | 42 |
| DLST | 6.85 | 48.7 | 9 |
| NDUFA13 | 6.84 | 16.7 | 22 |
| MBNL1 | 6.84 | 43 | 9 |
| HELZ2 | 6.83 | 294.5 | 3 |
| U2AF1 | 6.83 | 27.9 | 16 |
| | 6.82 | 73.3 | 18 |
| RPL26 | 6.81 | 17.2 | 18 |
| PSMC5 | 6.8 | 45.6 | 26 |
| BCLAF1 | 6.78 | 100.3 | 10 |
| RPL37A | 6.78 | 10.3 | 18 |
| | 6.77 | 129.2 | 6 |
| NUP133 | 6.76 | 122.2 | 7 |
| EMC1 | 6.75 | 111.7 | 9 |
| STRAP | 6.72 | 38.4 | 18 |

| | | | |
|---|---|---|---|
| DDX6 | 6.72 | 54.4 | 13 |
| RECQL | 6.69 | 73.4 | 16 |
| RPL6 | 6.68 | 32.7 | 20 |
| PLOD1 | 6.66 | 83.5 | 13 |
| RUVBL1 | 6.64 | 50.2 | 16 |
| EML4 | 6.62 | 108.8 | 18 |
| PCK2 | 6.6 | 70.7 | 7 |
| NLRP2 | 6.6 | 102.6 | 7 |
| NACA | 6.58 | 18.3 | 25 |
| LDHA | 6.55 | 33.6 | 12 |
| PPP2R2A | 6.55 | 51.6 | 6 |
| ERH | 6.54 | 12.3 | 46 |
| UFL1 | 6.52 | 89.5 | 10 |
| LASP1 | 6.52 | 29.7 | 15 |
| | 6.51 | 24 | 22 |
| CALM2 | 6.5 | 16.8 | 41 |
| SLC25A12 | 6.5 | 74.7 | 6 |
| HNRNPH3 | 6.5 | 36.9 | 16 |
| EIF6 | 6.5 | 11.8 | 39 |
| HNRNPA3 | 6.48 | 39.6 | 24 |
| ANKFY1 | 6.48 | 128.3 | 8 |
| PPP6R3 | 6.45 | 92.4 | 7 |
| | 6.44 | 197.7 | 4 |
| RPS4X | 6.43 | 27.2 | 23 |
| PTGES3 | 6.43 | 19.1 | 23 |
| IGF2R | 6.41 | 274.2 | 3 |
| EIF2AK2 | 6.41 | 61.5 | 8 |
| PFKM | 6.4 | 85.1 | 7 |
| UTP20 | 6.36 | 318.2 | 2 |
| DDX39B | 6.34 | 49 | 11 |
| XPO1 | 6.33 | 123.3 | 6 |
| SRSF5 | 6.33 | 31.2 | 18 |
| GALK1 | 6.32 | 42.2 | 15 |
| AHNAK | 6.29 | 628.7 | 2 |
| YWHAZ | 6.29 | 27.7 | 18 |
| HSPH1 | 6.27 | 92.1 | 6 |
| SNRPD3 | 6.27 | 13.9 | 25 |
| TRAP1 | 6.26 | 80 | 11 |
| ETFB | 6.25 | 27.8 | 22 |
| CFL1 | 6.24 | 18.5 | 28 |
| THBS1 | 6.22 | 129.3 | 9 |
| RPS16 | 6.2 | 14.4 | 43 |
| PPIG | 6.2 | 88.6 | 6 |
| RBM39 | 6.19 | 56.4 | 12 |
| SEC24C | 6.17 | 106.5 | 8 |
| EEF1G | 6.17 | 50.1 | 8 |
| DDX24 | 6.16 | 96.3 | 10 |
| BAZ1B | 6.15 | 170.8 | 4 |

| | | | |
|---|---|---|---|
| PPP3CA | 6.15 | 57.6 | 8 |
| HCFC1 | 6.15 | 208.6 | 2 |
| NUP88 | 6.12 | 83.5 | 9 |
| NUP98 | 6.11 | 197.5 | 7 |
| SNTB2 | 6.1 | 57.9 | 12 |
| SLC25A24 | 6.1 | 53.3 | 10 |
| | 6.08 | 81.5 | 6 |
| | 6.07 | 98.9 | 7 |
| CCT4 | 6.05 | 57.9 | 15 |
| SF3B3 | 6.05 | 135.5 | 5 |
| PFN1 | 6.03 | 15 | 42 |
| EIF4G1 | 6.02 | 158.5 | 8 |
| DHCR24 | 6.02 | 49.4 | 7 |
| AKAP17A | 6 | 80.7 | 10 |
| | 6 | 16.6 | 16 |
| PSMC3 | 5.98 | 49.2 | 21 |
| TSG101 | 5.98 | 29 | 10 |
| PHF6 | 5.97 | 41.3 | 27 |
| | 5.97 | 109.6 | 6 |
| GNL3 | 5.96 | 62 | 14 |
| LRRC40 | 5.96 | 68.2 | 6 |
| AP1M1 | 5.95 | 42.6 | 28 |
| | 5.95 | 21.4 | 16 |
| ACSL4 | 5.94 | 74.4 | 9 |
| SRSF6 | 5.93 | 39.5 | 15 |
| STRN3 | 5.93 | 87.2 | 5 |
| CNBP | 5.92 | 19.5 | 34 |
| VCP | 5.92 | 89.3 | 11 |
| GSPT1 | 5.92 | 55.7 | 13 |
| SKIV2L2 | 5.91 | 117.7 | 7 |
| RPLP2 | 5.91 | 11.7 | 24 |
| PDLIM7 | 5.9 | 49.8 | 20 |
| AP1G1 | 5.9 | 94.1 | 4 |
| MAP1B | 5.89 | 270.5 | 1 |
| YARS | 5.88 | 59.1 | 7 |
| PSMD2 | 5.87 | 100.1 | 17 |
| STT3B | 5.86 | 93.6 | 7 |
| ZCCHC8 | 5.85 | 78.5 | 6 |
| NARS | 5.85 | 34.9 | 12 |
| OPA1 | 5.83 | 113.4 | 9 |
| ATP2C1 | 5.82 | 100.5 | 3 |
| RPL36A | 5.82 | 16.4 | 13 |
| GNB1 | 5.79 | 37.3 | 9 |
| DSTN | 5.78 | 18.5 | 35 |
| CCT6A | 5.78 | 57.9 | 12 |
| DDX54 | 5.78 | 98.5 | 4 |
| PMPCB | 5.76 | 54.4 | 10 |
| TAOK2 | 5.75 | 93.9 | 7 |

| | | | |
|---|---|---|---|
| CCT7 | 5.74 | 59.3 | 11 |
| RAB3GAP2 | 5.74 | 155.9 | 4 |
| | 5.74 | 37.4 | 11 |
| CTPS1 | 5.73 | 66.6 | 11 |
| SRRT | 5.72 | 100.6 | 9 |
| NASP | 5.72 | 74.8 | 4 |
| | 5.71 | 13.5 | 29 |
| RPS9 | 5.71 | 22.6 | 17 |
| HLA-B | 5.7 | 40.4 | 11 |
| | 5.68 | 210 | 3 |
| CLPX | 5.68 | 69.2 | 9 |
| RXRB | 5.67 | 56.9 | 7 |
| AHCYL1 | 5.66 | 58.9 | 8 |
| DNM2 | 5.64 | 98 | 5 |
| RBMX | 5.63 | 42.3 | 17 |
| SLFN5 | 5.63 | 100.9 | 4 |
| SRPRB | 5.62 | 29.7 | 28 |
| MTA2 | 5.62 | 75 | 9 |
| POLR2A | 5.61 | 217 | 6 |
| ABCF3 | 5.61 | 79.7 | 8 |
| | 5.61 | 12.6 | 46 |
| NXF1 | 5.61 | 70.1 | 4 |
| TUBB4B | 5.58 | 21 | 29 |
| DNAJA2 | 5.58 | 45.7 | 11 |
| ARGLU1 | 5.58 | 33.2 | 14 |
| HLA-B | 5.57 | 21 | 13 |
| RPS27 | 5.55 | 9.5 | 40 |
| NCOA5 | 5.54 | 65.5 | 11 |
| HADHB | 5.54 | 51.3 | 8 |
| RSRC2 | 5.5 | 50.5 | 7 |
| SNRNP40 | 5.49 | 39.3 | 15 |
| AGPS | 5.49 | 72.9 | 7 |
| MRPS27 | 5.49 | 22 | 12 |
| ACOT7 | 5.47 | 41.8 | 11 |
| PFN2 | 5.46 | 15 | 26 |
| MCM5 | 5.41 | 82.2 | 7 |
| NUP160 | 5.39 | 162 | 4 |
| APOBEC3C | 5.39 | 22.8 | 10 |
| | 5.37 | 10.4 | 34 |
| MAT2A | 5.36 | 39.7 | 12 |
| MRPL46 | 5.31 | 31.7 | 13 |
| WDR18 | 5.31 | 47.4 | 7 |
| HBS1L | 5.3 | 75.4 | 11 |
| CTBP2 | 5.3 | 48.9 | 11 |
| FNDC3B | 5.29 | 132.8 | 8 |
| ZNF638 | 5.29 | 220.5 | 3 |
| ITGB1 | 5.28 | 88.4 | 5 |
| ACTR2 | 5.27 | 39 | 15 |

| | | | |
|---|---|---|---|
| HNRNPAB | 5.26 | 36.2 | 7 |
| HLA-DRA | 5.23 | 28.6 | 21 |
| RFC2 | 5.22 | 39.1 | 10 |
| TUBG1 | 5.21 | 51.1 | 10 |
| | 5.21 | 43.6 | 6 |
| MRPS22 | 5.2 | 41.3 | 12 |
| TUSC3 | 5.2 | 35.7 | 7 |
| FAF2 | 5.17 | 52.6 | 9 |
| | 5.16 | 25 | 14 |
| SREK1 | 5.16 | 58.2 | 5 |
| TAF15 | 5.15 | 61.8 | 10 |
| CPT1A | 5.13 | 88.3 | 14 |
| TFRC | 5.11 | 75.9 | 6 |
| DUSP23 | 5.1 | 16.6 | 22 |
| ACTG1 | 5.09 | 11.5 | 28 |
| | 5.06 | 20.5 | 22 |
| RPS19 | 5.02 | 16.1 | 21 |
| RBBP7 | 5.01 | 46.9 | 16 |
| | 5 | 31.4 | 23 |
| AQR | 4.99 | 171.2 | 2 |
| RING1 | 4.98 | 39.1 | 11 |
| | 4.97 | 40.4 | 12 |
| SRP72 | 4.94 | 74.6 | 12 |
| RNH1 | 4.93 | 49.9 | 16 |
| CAPZA1 | 4.93 | 32.9 | 31 |
| | 4.89 | 67 | 12 |
| LONP1 | 4.88 | 95 | 8 |
| CPSF7 | 4.88 | 52 | 8 |
| | 4.87 | 40.4 | 9 |
| PTPLAD1 | 4.87 | 43.1 | 6 |
| EHD1 | 4.86 | 61.9 | 10 |
| | 4.86 | 10.7 | 31 |
| AP3S1 | 4.86 | 21.7 | 10 |
| DNMT1 | 4.85 | 183.1 | 4 |
| USP10 | 4.84 | 87.1 | 6 |
| NUDT21 | 4.82 | 26.2 | 18 |
| EMC2 | 4.82 | 34.8 | 12 |
| RAB2A | 4.8 | 23.5 | 37 |
| | 4.8 | 17.1 | 20 |
| | 4.79 | 24.5 | 18 |
| PREB | 4.79 | 37.8 | 8 |
| | 4.77 | 32.8 | 19 |
| | 4.77 | 12.1 | 18 |
| ASF1A | 4.75 | 23 | 5 |
| RB1 | 4.73 | 109.7 | 6 |
| CCAR1 | 4.71 | 132.7 | 6 |
| SUCLG1 | 4.71 | 36.2 | 8 |
| | 4.69 | 12 | 16 |

| | | | |
|---|---|---|---|
| SRRM1 | 4.68 | 103.9 | 8 |
| UBAP2L | 4.64 | 113.5 | 8 |
| | 4.64 | 12.8 | 16 |
| RBM14 | 4.63 | 69.4 | 8 |
| HIST1H4A | 4.62 | 11.4 | 52 |
| PCBP1 | 4.62 | 37.5 | 12 |
| NDUFV3 | 4.61 | 49.2 | 6 |
| SEC23IP | 4.6 | 89.7 | 6 |
| ARPC4 | 4.6 | 19.7 | 21 |
| CANX | 4.58 | 67.5 | 9 |
| CMAS | 4.58 | 48.3 | 11 |
| | 4.58 | 16 | 26 |
| | 4.58 | 38 | 9 |
| NTPCR | 4.57 | 25.1 | 15 |
| ANKRD52 | 4.57 | 115 | 2 |
| RAB5C | 4.56 | 23.5 | 17 |
| CALU | 4.55 | 37.1 | 18 |
| MTCH2 | 4.55 | 33.3 | 18 |
| HLA-C | 4.54 | 41 | 17 |
| SSB | 4.53 | 46.8 | 16 |
| ATP6V1A | 4.53 | 68.3 | 6 |
| HLA-A | 4.52 | 38.2 | 27 |
| RPL36 | 4.51 | 12.2 | 30 |
| PSMD14 | 4.51 | 16.5 | 12 |
| SLC16A3 | 4.49 | 49.4 | 12 |
| BCAP31 | 4.47 | 27.9 | 17 |
| ILKAP | 4.47 | 42.9 | 7 |
| | 4.45 | 31.7 | 12 |
| | 4.44 | 274.2 | 9 |
| PSMB5 | 4.44 | 28.5 | 9 |
| CAD | 4.43 | 242.8 | 2 |
| SRP14 | 4.42 | 14.6 | 16 |
| TIMM44 | 4.41 | 51.3 | 13 |
| SUPT5H | 4.41 | 120.9 | 6 |
| SRSF9 | 4.41 | 25.5 | 17 |
| FUBP1 | 4.41 | 67.5 | 5 |
| SLC30A7 | 4.39 | 41.6 | 7 |
| CSE1L | 4.37 | 110.3 | 6 |
| UTP18 | 4.35 | 62 | 10 |
| | 4.35 | 33.3 | 8 |
| ACADM | 4.34 | 50.2 | 12 |
| ZFP91 | 4.34 | 63.3 | 6 |
| CKAP5 | 4.34 | 225.4 | 2 |
| FKBP5 | 4.34 | 51.2 | 6 |
| USP9X | 4.33 | 271.1 | 3 |
| CRNKL1 | 4.33 | 87.3 | 8 |
| IPO7 | 4.32 | 119.4 | 4 |
| VAPA | 4.31 | 27.9 | 25 |

| | | | |
|---|---|---|---|
| | 4.3 | 120.2 | 5 |
| | 4.3 | 208.3 | 2 |
| | 4.29 | 170.6 | 3 |
| BLOC1S5 | 4.28 | 40.3 | 8 |
| PWP1 | 4.27 | 55.8 | 3 |
| CHD3 | 4.26 | 226.5 | 2 |
| BMS1 | 4.24 | 145.7 | 5 |
| | 4.22 | 75.8 | 10 |
| TRMT112 | 4.21 | 14.2 | 17 |
| | 4.21 | 74.6 | 3 |
| FLOT2 | 4.2 | 41.7 | 6 |
| MAGEC2 | 4.2 | 41.1 | 4 |
| SAFB2 | 4.19 | 107.4 | 6 |
| NDUFV1 | 4.19 | 50 | 7 |
| | 4.19 | 32.7 | 7 |
| DDX19B | 4.17 | 53.9 | 10 |
| | 4.16 | 79.7 | 5 |
| ATP5F1 | 4.16 | 28.9 | 12 |
| PCID2 | 4.16 | 46 | 7 |
| SDHA | 4.15 | 72.6 | 8 |
| CNOT1 | 4.14 | 266.8 | 2 |
| SNRPD2 | 4.14 | 13.5 | 15 |
| EIF3H | 4.13 | 39.8 | 20 |
| | 4.13 | 129.3 | 4 |
| KDM5D | 4.13 | 102 | 2 |
| SEC16A | 4.12 | 210.9 | 3 |
| DLD | 4.12 | 48.9 | 6 |
| MFN2 | 4.12 | 71.4 | 3 |
| GNAI2 | 4.11 | 34.9 | 12 |
| TMEM165 | 4.11 | 34.9 | 10 |
| | 4.1 | 65.9 | 9 |
| HNRNPD | 4.1 | 32.8 | 17 |
| NCBP2 | 4.1 | 18 | 17 |
| MDH2 | 4.1 | 35.5 | 10 |
| | 4.1 | 6 | 49 |
| CSNK2B | 4.1 | 24.9 | 8 |
| STRN4 | 4.09 | 80.5 | 6 |
| SH3BGRL2 | 4.09 | 12.3 | 34 |
| DHX37 | 4.09 | 129.5 | 3 |
| MRE11A | 4.09 | 59.7 | 8 |
| | 4.09 | 48.1 | 4 |
| POLR2B | 4.07 | 133 | 9 |
| PYCR2 | 4.07 | 33.6 | 11 |
| | 4.07 | 29.3 | 8 |
| CCDC47 | 4.07 | 55.8 | 5 |
| TARDBP | 4.06 | 41.6 | 19 |
| MTIF2 | 4.06 | 56.1 | 6 |
| APOL2 | 4.05 | 37.1 | 15 |

| | | | |
|---|---|---|---|
| EXOC4 | 4.05 | 110.4 | 5 |
| DECR1 | 4.05 | 36 | 18 |
| | 4.05 | 47 | 4 |
| YWHAE | 4.05 | 29.2 | 14 |
| SHMT2 | 4.04 | 56 | 12 |
| DDX42 | 4.04 | 102.9 | 7 |
| EEF1D | 4.04 | 31.1 | 25 |
| PSMD13 | 4.04 | 42.9 | 12 |
| IFIT3 | 4.04 | 56 | 7 |
| KRT18 | 4.03 | 48 | 18 |
| | 4.03 | 22.7 | 14 |
| RPL14 | 4.03 | 23.4 | 15 |
| KIF2C | 4.02 | 81.3 | 9 |
| TM9SF3 | 4.02 | 60.1 | 6 |
| PDS5B | 4.02 | 164.6 | 2 |
| RPL18A | 4 | 18.1 | 34 |
| TAOK1 | 3.99 | 116 | 3 |
| | 3.98 | 85.3 | 4 |
| DNAJC16 | 3.98 | 90.5 | 4 |
| KCMF1 | 3.98 | 41.9 | 5 |
| SLC6A19 | 3.98 | 39.4 | 3 |
| RFC4 | 3.97 | 39.7 | 10 |
| THOC2 | 3.97 | 182.7 | 1 |
| ITM2B | 3.97 | 30.3 | 7 |
| AP2M1 | 3.97 | 52.3 | 5 |
| TBC1D5 | 3.97 | 88.9 | 2 |
| RANBP1 | 3.97 | 23.3 | 11 |
| MRPS23 | 3.96 | 21.8 | 27 |
| PAPOLA | 3.96 | 82.8 | 3 |
| LMAN1 | 3.95 | 57.5 | 11 |
| DDX56 | 3.95 | 61.5 | 8 |
| ECH1 | 3.95 | 35.8 | 6 |
| ALDH18A1 | 3.95 | 87.2 | 2 |
| NFKB2 | 3.94 | 96.7 | 2 |
| NPM1 | 3.93 | 30 | 20 |
| TWF1 | 3.93 | 40.3 | 11 |
| PTPN1 | 3.92 | 49.9 | 9 |
| ZMPSTE24 | 3.92 | 27.1 | 16 |
| ACTR1A | 3.92 | 38.3 | 15 |
| PRKRA | 3.91 | 34.4 | 13 |
| FKBP4 | 3.91 | 51.8 | 6 |
| TRAPPC11 | 3.91 | 128.8 | 2 |
| TAOK2 | 3.9 | 138.2 | 4 |
| ACAA2 | 3.9 | 41.9 | 11 |
| GALE | 3.9 | 38.3 | 10 |
| CAP2 | 3.9 | 39.9 | 7 |
| PWP2 | 3.9 | 102.4 | 2 |
| MAGEB2 | 3.9 | 35.2 | 7 |

| | | | |
|---|---|---|---|
| | 3.9 | 7.9 | 20 |
| TCOF1 | 3.89 | 152.2 | 4 |
| RPL34 | 3.89 | 13.3 | 8 |
| AP1B1 | 3.88 | 104.6 | 7 |
| ATP6V1H | 3.88 | 55.8 | 6 |
| GBP1 | 3.87 | 67.9 | 7 |
| WDR3 | 3.87 | 106 | 2 |
| GTF3C1 | 3.85 | 238 | 3 |
| SUCLA2 | 3.85 | 43.8 | 9 |
| | 3.84 | 66 | 6 |
| FXR2 | 3.84 | 74.2 | 6 |
| STK4 | 3.84 | 55.6 | 5 |
| FAM208A | 3.84 | 188.9 | 1 |
| NOL10 | 3.83 | 80.3 | 6 |
| SRP9 | 3.82 | 10.1 | 47 |
| MT1X | 3.8 | 6.1 | 34 |
| TRIP4 | 3.8 | 66.1 | 7 |
| ATP2B4 | 3.8 | 133.8 | 3 |
| UBE3C | 3.79 | 123.8 | 4 |
| PAFAH1B3 | 3.79 | 25.7 | 8 |
| CTNND1 | 3.78 | 104.8 | 6 |
| STAU2 | 3.78 | 62.6 | 7 |
| EXOSC10 | 3.78 | 100.8 | 4 |
| FHOD1 | 3.78 | 129.2 | 3 |
| ITPA | 3.78 | 21.4 | 14 |
| SNRPA | 3.78 | 31.3 | 13 |
| TCEB1 | 3.77 | 12.5 | 40 |
| | 3.76 | 77.1 | 9 |
| SSBP1 | 3.76 | 17.2 | 22 |
| SUPT4H1 | 3.76 | 13.2 | 14 |
| | 3.75 | 118.2 | 7 |
| TKT | 3.75 | 38.9 | 16 |
| FAM129B | 3.75 | 82.6 | 4 |
| SEC61A1 | 3.75 | 52.9 | 6 |
| MAGED1 | 3.75 | 86.1 | 3 |
| | 3.75 | 71.4 | 5 |
| DNAJB11 | 3.74 | 40.5 | 24 |
| RHOA | 3.74 | 21.8 | 26 |
| | 3.74 | 15.7 | 32 |
| COMMD2 | 3.74 | 22.7 | 4 |
| H2AFY | 3.73 | 39.6 | 14 |
| | 3.73 | 166.6 | 2 |
| FKBP10 | 3.72 | 64.2 | 11 |
| MFAP1 | 3.72 | 51.9 | 5 |
| ASCC1 | 3.72 | 41.2 | 4 |
| RRP9 | 3.71 | 51.8 | 13 |
| | 3.71 | 33.3 | 11 |
| MAP1S | 3.71 | 112.2 | 3 |

| | | | |
|---|---|---|---|
| PDIA5 | 3.7 | 59.6 | 6 |
| SAMD9L | 3.69 | 184.4 | 1 |
| OGT | 3.68 | 116.9 | 7 |
| CAPNS1 | 3.68 | 28.3 | 13 |
| AKAP8L | 3.68 | 71.6 | 5 |
| FAF1 | 3.67 | 73.9 | 3 |
| GNA13 | 3.66 | 40.9 | 7 |
| | 3.65 | 160.8 | 3 |
| DHRS4 | 3.64 | 29.5 | 17 |
| SPATA5L1 | 3.64 | 80.7 | 5 |
| RPS20 | 3.64 | 13.4 | 18 |
| IDH1 | 3.64 | 46.6 | 10 |
| | 3.64 | 15.6 | 29 |
| | 3.64 | 17.5 | 16 |
| NRP1 | 3.64 | 79 | 3 |
| TMOD2 | 3.63 | 39.6 | 7 |
| | 3.62 | 55.4 | 14 |
| YWHAB | 3.62 | 28.1 | 15 |
| | 3.62 | 27.4 | 10 |
| PPFIBP1 | 3.61 | 114 | 7 |
| CDK5 | 3.61 | 33.3 | 7 |
| RTN4 | 3.61 | 36.9 | 6 |
| TROVE2 | 3.6 | 60.6 | 7 |
| | 3.6 | 44.2 | 9 |
| VPS35 | 3.59 | 91.6 | 5 |
| | 3.59 | 97.7 | 2 |
| | 3.56 | 54.7 | 8 |
| NPLOC4 | 3.56 | 68.1 | 6 |
| VPS18 | 3.55 | 110.1 | 5 |
| WDR6 | 3.55 | 124.9 | 1 |
| FARSB | 3.54 | 66.1 | 10 |
| TEX11 | 3.54 | 107.8 | 6 |
| H1FX | 3.54 | 22.5 | 13 |
| | 3.54 | 36.6 | 10 |
| LUC7L3 | 3.53 | 51.4 | 6 |
| PRDX1 | 3.52 | 22.1 | 38 |
| BMP1 | 3.52 | 111.2 | 5 |
| | 3.51 | 29.3 | 8 |
| PBXIP1 | 3.5 | 57.6 | 9 |
| RALY | 3.5 | 30.4 | 26 |
| VWA8 | 3.49 | 214.7 | 2 |
| PSMC1 | 3.48 | 49.2 | 7 |
| TIMM21 | 3.48 | 28.2 | 14 |
| IDH3B | 3.47 | 42.2 | 10 |
| RPL23 | 3.46 | 14.9 | 29 |
| SF3A3 | 3.45 | 58.7 | 14 |
| PARP14 | 3.45 | 202.7 | 3 |
| GNB4 | 3.45 | 37.5 | 11 |

| | | | |
|---|---|---|---|
| | 3.45 | 18.6 | 14 |
| AKAP2 | 3.45 | 94.6 | 2 |
| RPS11 | 3.45 | 18.4 | 10 |
| DNAJC9 | 3.44 | 29.9 | 12 |
| EIF3F | 3.43 | 39.1 | 10 |
| | 3.43 | 92.6 | 5 |
| ZFR | 3.4 | 116.9 | 5 |
| | 3.4 | 23.9 | 13 |
| | 3.39 | 118.3 | 8 |
| GYS1 | 3.39 | 83.8 | 4 |
| NCAPG | 3.38 | 114.1 | 5 |
| MRPS2 | 3.38 | 35.5 | 8 |
| MBOAT7 | 3.36 | 52.7 | 8 |
| ASH2L | 3.35 | 68.7 | 6 |
| | 3.33 | 112.1 | 7 |
| NOL6 | 3.33 | 78 | 9 |
| TRRAP | 3.33 | 405.6 | 1 |
| PRDX2 | 3.32 | 20.1 | 10 |
| | 3.31 | 31.4 | 6 |
| PPIB | 3.3 | 22.7 | 27 |
| EIF2A | 3.3 | 64.9 | 8 |
| RRS1 | 3.3 | 41.2 | 7 |
| MVP | 3.28 | 87.5 | 6 |
| NUP205 | 3.26 | 227.8 | 5 |
| RHOT1 | 3.25 | 70.7 | 3 |
| RRP1B | 3.24 | 84.4 | 8 |
| ALB | 3.21 | 69.3 | 6 |
| DDX10 | 3.16 | 85.8 | 6 |
| | 3.11 | 16.9 | 16 |
| LEPREL4 | 3.11 | 49.1 | 3 |
| OSBPL3 | 3.04 | 101.2 | 1 |
| PELO | 3.01 | 43.4 | 9 |
| ARPC2 | 3 | 34.3 | 13 |
| PRPF38A | 2.95 | 37.5 | 4 |
| | 2.93 | 20.2 | 8 |
| RAB14 | 2.9 | 20.4 | 16 |
| MRPL48 | 2.89 | 23.9 | 10 |
| DAPK3 | 2.87 | 22.2 | 7 |
| | 2.87 | 26.3 | 4 |
| | 2.86 | 20.9 | 7 |
| EMD | 2.85 | 29 | 12 |
| SPG20 | 2.84 | 40.6 | 13 |
| PRRC2C | 2.84 | 316.7 | 1 |
| NMNAT1 | 2.83 | 31.9 | 8 |
| SMARCB1 | 2.82 | 45 | 5 |
| AKR1A1 | 2.81 | 36.6 | 4 |
| ACTR2 | 2.8 | 44.7 | 13 |
| HLA-C | 2.8 | 21.2 | 15 |

| | | | |
|---|---|---|---|
| FAM98A | 2.8 | 34.1 | 5 |
| | 2.78 | 112.9 | 3 |
| PRKCA | 2.78 | 76.7 | 3 |
| HLA-C | 2.78 | 21.2 | 10 |
| NOC2L | 2.76 | 84.9 | 5 |
| RNF114 | 2.75 | 25.7 | 9 |
| MRPL55 | 2.73 | 6.8 | 27 |
| CDC42BPA | 2.71 | 197.2 | 3 |
| PDIA4 | 2.71 | 72.9 | 2 |
| | 2.7 | 62.4 | 9 |
| | 2.69 | 19.8 | 28 |
| VPS39 | 2.68 | 101.7 | 1 |
| FAHD2A | 2.67 | 34.6 | 7 |
| | 2.66 | 8.5 | 14 |
| BLVRB | 2.65 | 22.1 | 12 |
| | 2.65 | 45.6 | 3 |
| ITGA11 | 2.64 | 133.4 | 1 |
| TRIM3 | 2.63 | 42.6 | 3 |
| KPNB1 | 2.6 | 97.1 | 5 |
| SNRPF | 2.6 | 9.7 | 24 |
| | 2.59 | 38.2 | 8 |
| | 2.59 | 35.1 | 4 |
| LYAR | 2.58 | 43.6 | 17 |
| TRIM25 | 2.58 | 72.2 | 7 |
| ARHGAP1 | 2.58 | 52.7 | 4 |
| | 2.57 | 17.3 | 7 |
| LEMD2 | 2.56 | 56.9 | 4 |
| | 2.55 | 18.2 | 6 |
| CALR | 2.54 | 46.9 | 13 |
| | 2.54 | 79.7 | 1 |
| ABCB7 | 2.53 | 78.2 | 4 |
| | 2.53 | 92.8 | 2 |
| WDR83 | 2.53 | 34.3 | 4 |
| ANXA11 | 2.51 | 65.6 | 2 |
| | 2.5 | 84.3 | 7 |
| SUN1 | 2.5 | 90.1 | 3 |
| KIF1B | 2.5 | 203.5 | 1 |
| NDUFS2 | 2.48 | 46.4 | 6 |
| | 2.48 | 40.6 | 4 |
| SSR4 | 2.48 | 19 | 8 |
| LTV1 | 2.47 | 54.8 | 8 |
| NUP107 | 2.47 | 106.3 | 3 |
| PSMD10 | 2.47 | 20.8 | 15 |
| PSMD12 | 2.47 | 52.9 | 4 |
| STARD9 | 2.47 | 516 | 0 |
| | 2.46 | 15.6 | 16 |
| SEC63 | 2.46 | 47.9 | 5 |
| | 2.46 | 21.7 | 7 |

| | | | |
|---|---|---|---|
| | 2.45 | 76.9 | 3 |
| TWISTNB | 2.45 | 37.4 | 4 |
| | 2.45 | 46.8 | 5 |
| F2 | 2.45 | 33.6 | 5 |
| NEMF | 2.44 | 122.9 | 2 |
| | 2.44 | 60.9 | 2 |
| AFAP1 | 2.44 | 80.7 | 2 |
| NR1H2 | 2.44 | 51.1 | 2 |
| | 2.43 | 307.6 | 2 |
| | 2.43 | 29.1 | 11 |
| | 2.43 | 17.9 | 8 |
| | 2.43 | 94.5 | 2 |
| | 2.43 | 24.8 | 4 |
| PDIA3 | 2.42 | 54.1 | 16 |
| HBA2 | 2.41 | 15.2 | 22 |
| PRKD2 | 2.41 | 96.7 | 2 |
| C2orf18 | 2.41 | 31.1 | 4 |
| SRPR | 2.4 | 69.9 | 8 |
| METAP1 | 2.4 | 37.8 | 6 |
| RAP2C | 2.4 | 20.7 | 5 |
| ACOT8 | 2.4 | 20.8 | 5 |
| PRPF19 | 2.39 | 55.1 | 11 |
| WDR43 | 2.39 | 74.8 | 8 |
| H2AFY2 | 2.39 | 40 | 7 |
| ACP1 | 2.39 | 18.7 | 7 |
| | 2.39 | 31.6 | 4 |
| KLC1 | 2.38 | 68.7 | 7 |
| MAGEA1 | 2.38 | 34.3 | 9 |
| | 2.38 | 20.9 | 17 |
| ETHE1 | 2.38 | 24.9 | 10 |
| GET4 | 2.37 | 36.5 | 4 |
| MLLT11 | 2.36 | 10.1 | 13 |
| RBM8A | 2.36 | 19.9 | 11 |
| TJP2 | 2.35 | 117.7 | 4 |
| RPS28 | 2.35 | 7.8 | 16 |
| RDX | 2.34 | 40.7 | 17 |
| TMCO1 | 2.34 | 19.9 | 19 |
| PYCRL | 2.34 | 28.6 | 7 |
| HSPA4 | 2.34 | 52.3 | 5 |
| UBTF | 2.34 | 58.3 | 5 |
| XPO7 | 2.34 | 99.4 | 1 |
| | 2.33 | 11.7 | 10 |
| | 2.33 | 37 | 5 |
| | 2.33 | 11.1 | 13 |
| MOV10 | 2.32 | 107.1 | 5 |
| | 2.32 | 15.2 | 8 |
| PIGK | 2.31 | 45.2 | 3 |
| NCOR2 | 2.31 | 267.9 | 0 |

| | | | |
|---|---|---|---|
| TRAFD1 | 2.3 | 64.8 | 7 |
| CPSF4 | 2.3 | 30.2 | 13 |
| TRA2A | 2.3 | 32.7 | 8 |
| | 2.3 | 84.5 | 2 |
| KRT86 | 2.29 | 53.5 | 7 |
| PHF14 | 2.29 | 106.9 | 4 |
| RRP12 | 2.29 | 143.6 | 3 |
| TMED10 | 2.29 | 25 | 10 |
| NPEPPS | 2.29 | 85.2 | 5 |
| PRPS2 | 2.29 | 34.7 | 11 |
| OGDH | 2.29 | 99 | 3 |
| RALYL | 2.29 | 32.3 | 7 |
| CLNS1A | 2.29 | 18.2 | 8 |
| RIOK1 | 2.29 | 38 | 4 |
| RPS15A | 2.29 | 14.8 | 10 |
| G3BP1 | 2.28 | 52.1 | 13 |
| | 2.28 | 94 | 4 |
| EDARADD | 2.28 | 42.3 | 5 |
| DYNLL2 | 2.28 | 10.3 | 25 |
| TM9SF2 | 2.28 | 65.4 | 5 |
| | 2.28 | 29.4 | 4 |
| NKRF | 2.27 | 77.6 | 5 |
| SH3BP4 | 2.27 | 107.4 | 2 |
| LOC100132015 | 2.27 | 78.8 | 2 |
| | 2.26 | 92.3 | 7 |
| PAF1 | 2.26 | 59.9 | 9 |
| EWSR1 | 2.26 | 64.9 | 2 |
| RANGAP1 | 2.26 | 63.5 | 6 |
| HDAC2 | 2.26 | 51.9 | 9 |
| STRN | 2.26 | 86.1 | 2 |
| NDUFB4 | 2.26 | 15.2 | 8 |
| | 2.25 | 112.9 | 3 |
| ZNF326 | 2.25 | 65.6 | 7 |
| PSMC4 | 2.25 | 47.3 | 7 |
| NUSAP1 | 2.25 | 49.4 | 5 |
| TFIP11 | 2.25 | 96.7 | 4 |
| FDXR | 2.25 | 58.2 | 4 |
| HAT1 | 2.24 | 49.5 | 7 |
| PPIA | 2.24 | 14.1 | 16 |
| | 2.24 | 90.6 | 2 |
| ZNF598 | 2.24 | 98.6 | 1 |
| | 2.24 | 136.8 | 1 |
| NEXN | 2.24 | 46.1 | 3 |
| | 2.23 | 52.7 | 5 |
| MAGT1 | 2.23 | 38 | 5 |
| NUP85 | 2.23 | 75 | 2 |
| NTMT1 | 2.23 | 25.4 | 7 |
| CPD | 2.23 | 152.8 | 1 |

| | | | |
|---|---|---|---|
| NUP188 | 2.22 | 195.9 | 1 |
| MRPL24 | 2.22 | 24.9 | 10 |
| | 2.22 | 7.8 | 13 |
| RAB3GAP1 | 2.21 | 99.1 | 6 |
| UBR5 | 2.21 | 309.2 | 1 |
| METTL3 | 2.21 | 33.1 | 7 |
| SETDB1 | 2.21 | 143.1 | 1 |
| NIPSNAP1 | 2.21 | 31.4 | 3 |
| | 2.2 | 20.9 | 22 |
| HLA-A | 2.2 | 31.5 | 16 |
| PATL1 | 2.2 | 86.8 | 4 |
| CAMK2D | 2.2 | 60 | 4 |
| RAB7L1 | 2.2 | 23.2 | 5 |
| HM13 | 2.19 | 38 | 11 |
| CLTA | 2.19 | 21.1 | 11 |
| NOL9 | 2.19 | 79.3 | 3 |
| ARAF | 2.19 | 68 | 3 |
| ZNF207 | 2.19 | 52.8 | 7 |
| | 2.19 | 56.2 | 3 |
| TRA2B | 2.18 | 29.2 | 15 |
| LPCAT1 | 2.18 | 59.1 | 6 |
| SUB1 | 2.18 | 15.1 | 15 |
| PTK2 | 2.18 | 84.4 | 1 |
| SPATA5 | 2.17 | 97.8 | 4 |
| | 2.17 | 89.3 | 3 |
| EIF2B4 | 2.17 | 57.5 | 2 |
| | 2.16 | 18.6 | 28 |
| KRAS | 2.16 | 21.6 | 20 |
| TXN | 2.16 | 11.7 | 12 |
| FAU | 2.16 | 6.6 | 17 |
| L3HYPDH | 2.16 | 38.1 | 3 |
| | 2.15 | 49.8 | 8 |
| ACTL6A | 2.15 | 47.4 | 8 |
| | 2.15 | 17.5 | 16 |
| RFC3 | 2.15 | 40.5 | 5 |
| SRM | 2.15 | 33.8 | 7 |
| LIN7C | 2.15 | 21.8 | 6 |
| DHX33 | 2.14 | 78.8 | 6 |
| | 2.14 | 21.8 | 10 |
| RAB27A | 2.14 | 24.9 | 6 |
| SNRPB2 | 2.14 | 25.5 | 8 |
| TMX2 | 2.14 | 34 | 5 |
| GEMIN2 | 2.14 | 31.6 | 4 |
| PSMA1 | 2.14 | 29.5 | 4 |
| DOCK10 | 2.13 | 249.4 | 2 |
| IK | 2.13 | 65.6 | 7 |
| HLA-DRB1 | 2.13 | 10.9 | 19 |
| ARHGDIA | 2.13 | 23.2 | 7 |

| | | | |
|---|---|---|---|
| | 2.13 | 7.6 | 20 |
| | 2.12 | 155.2 | 1 |
| KIAA0196 | 2.12 | 117 | 1 |
| KIF21A | 2.12 | 187.1 | 1 |
| PDLIM5 | 2.12 | 48.9 | 2 |
| GMPPB | 2.12 | 39.8 | 3 |
| ACSL1 | 2.11 | 74.2 | 8 |
| PDS5A | 2.11 | 150.7 | 3 |
| PDXDC1 | 2.11 | 86.7 | 4 |
| SLC16A1 | 2.11 | 51.8 | 7 |
| PDE5A | 2.11 | 99.9 | 3 |
| FAM120C | 2.11 | 99.3 | 4 |
| CTSA | 2.11 | 54.2 | 4 |
| MKI67 | 2.11 | 358.5 | 1 |
| MRPL43 | 2.11 | 17.7 | 7 |
| CLCN7 | 2.11 | 82.6 | 2 |
| MTCH1 | 2.11 | 39.7 | 3 |
| EXT2 | 2.11 | 38.3 | 3 |
| HSDL2 | 2.11 | 37.3 | 3 |
| SNW1 | 2.11 | 33.4 | 3 |
| SURF4 | 2.11 | 30.4 | 4 |
| HMHA1 | 2.11 | 2.7 | 38 |
| KRT77 | 2.1 | 61.9 | 7 |
| KRR1 | 2.1 | 43.6 | 6 |
| SERPINE2 | 2.1 | 37.1 | 4 |
| PNKP | 2.1 | 57 | 2 |
| BAG6 | 2.1 | 118.6 | 1 |
| MAN1A2 | 2.1 | 32.7 | 4 |
| ZAK | 2.1 | 40.2 | 3 |
| SKIV2L | 2.09 | 137.7 | 6 |
| SRPK1 | 2.09 | 74.3 | 9 |
| | 2.09 | 215.2 | 1 |
| TTLL12 | 2.09 | 74.4 | 4 |
| PURA | 2.09 | 34.9 | 6 |
| AGPAT6 | 2.09 | 52 | 2 |
| TGFB1I1 | 2.08 | 47.9 | 10 |
| FTH1 | 2.08 | 26.2 | 12 |
| GMDS | 2.08 | 41.9 | 8 |
| DNAJB6 | 2.08 | 23.8 | 9 |
| RPL24 | 2.08 | 17.8 | 11 |
| GNAI3 | 2.07 | 40.5 | 8 |
| | 2.07 | 243.2 | 2 |
| SAR1A | 2.07 | 22.3 | 10 |
| RHOG | 2.07 | 21.3 | 7 |
| WDR46 | 2.07 | 61.7 | 4 |
| GAPVD1 | 2.07 | 164.9 | 1 |
| | 2.06 | 42.9 | 11 |
| NDUFAF3 | 2.06 | 20.3 | 6 |

| | | | |
|---|---|---|---|
| NEDD1 | 2.06 | 71.9 | 3 |
| CCAR2 | 2.05 | 102.8 | 11 |
| | 2.05 | 43.6 | 8 |
| | 2.05 | 50 | 6 |
| ALKBH5 | 2.05 | 44.2 | 3 |
| ILVBL | 2.05 | 56.7 | 2 |
| NSA2 | 2.05 | 22.6 | 7 |
| | 2.04 | 28.1 | 13 |
| TRAF2 | 2.04 | 50.6 | 7 |
| XRN2 | 2.04 | 99.9 | 4 |
| SMU1 | 2.04 | 57.5 | 4 |
| CFI | 2.04 | 35.9 | 8 |
| UBR1 | 2.04 | 200.1 | 1 |
| NDUFS8 | 2.04 | 23.7 | 6 |
| PTPLB | 2.04 | 28.4 | 4 |
| PTCD3 | 2.04 | 78.5 | 1 |
| NUP214 | 2.04 | 213.5 | 0 |
| | 2.03 | 27 | 8 |
| FMR1 | 2.03 | 63.9 | 7 |
| HIST2H3PS2 | 2.03 | 15.4 | 20 |
| SPANXD | 2.03 | 11 | 24 |
| HIST3H3 | 2.03 | 15.5 | 20 |
| HLA-DPB1 | 2.03 | 10.5 | 30 |
| FTSJ3 | 2.02 | 82.5 | 10 |
| NGDN | 2.02 | 35.9 | 15 |
| | 2.02 | 20.2 | 14 |
| NUP54 | 2.02 | 55.3 | 4 |
| LEPREL1 | 2.02 | 80.9 | 2 |
| USMG5 | 2.02 | 6.5 | 28 |
| YY1 | 2.02 | 44.7 | 4 |
| | 2.02 | 177.7 | 1 |
| PYCR1 | 2.01 | 30.2 | 9 |
| GNE | 2.01 | 79.3 | 3 |
| | 2.01 | 52.5 | 3 |
| CDC37 | 2.01 | 44.4 | 4 |
| | 2.01 | 12.1 | 12 |
| FUS | 2 | 53.4 | 10 |
| EEA1 | 2 | 162.4 | 4 |
| | 2 | 186.3 | 2 |
| | 2 | 33.6 | 10 |
| NBAS | 2 | 176.2 | 2 |
| DRG1 | 2 | 40.5 | 5 |
| EXOSC9 | 2 | 46.9 | 3 |
| PTPN23 | 2 | 164.5 | 1 |
| POLR2E | 2 | 13.5 | 15 |
| EIF4H | 2 | 17.1 | 5 |
| | 1.99 | 206.8 | 3 |
| HTRA1 | 1.99 | 48.4 | 10 |

| | | | |
|---|---|---|---|
| NAV1 | 1.99 | 197.3 | 0 |
| | 1.99 | 20.1 | 7 |
| NAA15 | 1.98 | 101.2 | 10 |
| KANK2 | 1.98 | 91.1 | 3 |
| C3orf17 | 1.98 | 53.9 | 4 |
| PTBP1 | 1.98 | 56.5 | 3 |
| TTC37 | 1.98 | 175.4 | 1 |
| CHTOP | 1.98 | 23.6 | 10 |
| TOMM22 | 1.98 | 15.5 | 8 |
| NCBP1 | 1.98 | 91.8 | 1 |
| PPP1R12A | 1.98 | 115.2 | 1 |
| CDKAL1 | 1.98 | 65.1 | 2 |
| ARL2 | 1.98 | 20.9 | 7 |
| RPL29 | 1.98 | 17.5 | 10 |
| TRIP12 | 1.97 | 220.3 | 4 |
| HSD17B12 | 1.97 | 33.5 | 15 |
| UTP6 | 1.97 | 48 | 8 |
| NUP155 | 1.97 | 155.1 | 2 |
| SCFD1 | 1.97 | 51.5 | 5 |
| | 1.97 | 102.3 | 3 |
| TES | 1.97 | 48 | 7 |
| METTL7B | 1.97 | 27.8 | 9 |
| | 1.97 | 32.5 | 6 |
| | 1.97 | 47.8 | 3 |
| RPS26 | 1.97 | 13 | 19 |
| | 1.97 | 134.3 | 1 |
| | 1.97 | 7.6 | 14 |
| ACOT9 | 1.96 | 49.9 | 11 |
| CHD8 | 1.96 | 290.3 | 2 |
| | 1.96 | 21.2 | 11 |
| ANGPTL2 | 1.96 | 57 | 3 |
| | 1.96 | 128.3 | 1 |
| EPHX1 | 1.96 | 52.9 | 4 |
| BCAS2 | 1.96 | 26.1 | 5 |
| HOOK3 | 1.96 | 83.1 | 1 |
| WDR33 | 1.96 | 145.8 | 1 |
| S100A6 | 1.96 | 10.1 | 8 |
| LOXL2 | 1.95 | 86.7 | 4 |
| CUL7 | 1.95 | 191 | 2 |
| CPNE3 | 1.95 | 60.1 | 4 |
| RFC1 | 1.95 | 128.2 | 2 |
| EMILIN1 | 1.95 | 106.6 | 3 |
| SLC35B2 | 1.95 | 45 | 5 |
| DNAJC25 | 1.95 | 42.4 | 3 |
| CC2D1A | 1.94 | 104 | 3 |
| MIB1 | 1.94 | 110.1 | 2 |
| PSPC1 | 1.94 | 27.3 | 7 |
| LPGAT1 | 1.94 | 43.1 | 3 |

| | | | |
|---|---|---|---|
| RPA3 | 1.94 | 9.2 | 12 |
| SARS | 1.94 | 61.3 | 3 |
| KIF16B | 1.94 | 151.9 | 1 |
| RABL3 | 1.93 | 26.4 | 8 |
| | 1.93 | 21.1 | 20 |
| IFIT1 | 1.93 | 55.3 | 6 |
| PLCD3 | 1.93 | 89.2 | 5 |
| CYB5R1 | 1.93 | 34.1 | 4 |
| | 1.93 | 17.2 | 7 |
| | 1.93 | 76.8 | 2 |
| CYP51A1 | 1.92 | 57.2 | 7 |
| DNAJA3 | 1.92 | 52.5 | 6 |
| CIAO1 | 1.92 | 37.8 | 8 |
| DDX20 | 1.92 | 92.2 | 3 |
| | 1.92 | 87.1 | 2 |
| GLYR1 | 1.92 | 60.9 | 2 |
| AHCTF1 | 1.92 | 252.3 | 0 |
| RPS6KA2 | 1.92 | 85.6 | 1 |
| | 1.92 | 20 | 5 |
| CCZ1B | 1.92 | 39.5 | 3 |
| | 1.92 | 31.1 | 4 |
| | 1.91 | 54.6 | 9 |
| DDOST | 1.91 | 50.8 | 9 |
| SURF6 | 1.91 | 41.4 | 10 |
| RELA | 1.91 | 42.8 | 8 |
| TIAL1 | 1.91 | 41.6 | 5 |
| | 1.91 | 100 | 2 |
| | 1.91 | 20.1 | 10 |
| SULF2 | 1.91 | 100.4 | 1 |
| RPS7 | 1.91 | 22.1 | 14 |
| | 1.91 | 22.7 | 6 |
| PHRF1 | 1.91 | 178.3 | 1 |
| NUDCD3 | 1.91 | 24.7 | 5 |
| VAV2 | 1.91 | 101.2 | 1 |
| HTATSF1 | 1.91 | 25.2 | 3 |
| NCAPD2 | 1.9 | 157.1 | 3 |
| | 1.9 | 43.6 | 11 |
| SUCLG2 | 1.9 | 38.7 | 5 |
| PPP6C | 1.9 | 35.1 | 7 |
| SF3B4 | 1.9 | 44.5 | 2 |
| PRPF38B | 1.9 | 64.4 | 1 |
| KIAA0020 | 1.9 | 57.4 | 2 |
| SCO2 | 1.9 | 29.8 | 4 |
| | 1.9 | 166.7 | 0 |
| GIT2 | 1.89 | 67.1 | 5 |
| ATP5O | 1.89 | 23.3 | 15 |
| SLC25A11 | 1.89 | 34 | 8 |
| | 1.89 | 73.4 | 5 |

| | | | |
|---|---|---|---|
| CSTF1 | 1.89 | 46.8 | 5 |
| | 1.89 | 21 | 8 |
| | 1.89 | 114.5 | 1 |
| FSCN1 | 1.88 | 54.5 | 12 |
| OSBPL8 | 1.88 | 82.5 | 7 |
| NDUFA5 | 1.88 | 13.5 | 31 |
| | 1.88 | 45.5 | 6 |
| MLLT4 | 1.88 | 201.7 | 1 |
| CORO1B | 1.88 | 54.2 | 3 |
| | 1.88 | 133 | 2 |
| TCEB2 | 1.88 | 13.1 | 13 |
| PPP2CA | 1.88 | 35.5 | 7 |
| TMEM2 | 1.88 | 73.2 | 2 |
| EZH2 | 1.88 | 85.3 | 1 |
| ATP6V0D1 | 1.88 | 44.6 | 3 |
| LMAN2 | 1.88 | 40.2 | 3 |
| PGAM1 | 1.88 | 28.8 | 4 |
| TRIM56 | 1.87 | 81.4 | 8 |
| MMS19 | 1.87 | 113.2 | 3 |
| ZNF579 | 1.87 | 60.5 | 7 |
| AVEN | 1.87 | 38.5 | 3 |
| FNDC3A | 1.87 | 125.7 | 1 |
| PXN | 1.87 | 44.5 | 5 |
| | 1.87 | 15.9 | 7 |
| CLPTM1 | 1.87 | 76 | 1 |
| RHOT2 | 1.86 | 68.1 | 13 |
| | 1.86 | 16.7 | 13 |
| RARS2 | 1.86 | 65.5 | 4 |
| | 1.86 | 21.9 | 20 |
| SAFB | 1.86 | 91 | 2 |
| GEMIN5 | 1.86 | 168.3 | 2 |
| | 1.86 | 17.2 | 6 |
| RPL12 | 1.86 | 17.8 | 9 |
| CHAF1B | 1.86 | 61.5 | 2 |
| CBR1 | 1.86 | 30.4 | 3 |
| NOL11 | 1.86 | 81.1 | 1 |
| CUL2 | 1.85 | 86.9 | 4 |
| CD2BP2 | 1.85 | 37.6 | 7 |
| APMAP | 1.85 | 46.5 | 8 |
| KPNA2 | 1.85 | 57.8 | 5 |
| CENPB | 1.85 | 65.1 | 6 |
| SNRPA1 | 1.85 | 28.4 | 10 |
| SEC62 | 1.85 | 45.8 | 4 |
| | 1.85 | 161.1 | 1 |
| COX11 | 1.85 | 31.4 | 4 |
| DHCR7 | 1.85 | 54.4 | 5 |
| SNRPG | 1.85 | 7.1 | 19 |
| NHP2 | 1.85 | 17.2 | 7 |

| | | | |
|---|---|---|---|
| | 1.85 | 6.1 | 20 |
| PHAX | 1.85 | 44.4 | 2 |
| AMPD2 | 1.85 | 100.7 | 1 |
| | 1.85 | 55.2 | 3 |
| ESF1 | 1.85 | 63.4 | 2 |
| | 1.84 | 79.3 | 6 |
| DDX31 | 1.84 | 94 | 5 |
| TP53BP1 | 1.84 | 213.4 | 2 |
| VPS13C | 1.84 | 422.1 | 1 |
| SETD1A | 1.84 | 185.9 | 1 |
| NCSTN | 1.84 | 78.4 | 2 |
| | 1.84 | 9.2 | 27 |
| RBM17 | 1.84 | 24.2 | 6 |
| SNX7 | 1.84 | 45.3 | 2 |
| | 1.84 | 96 | 1 |
| DYNLRB1 | 1.84 | 13.4 | 8 |
| DOCK7 | 1.83 | 242.4 | 1 |
| | 1.83 | 154.9 | 2 |
| PRDX4 | 1.83 | 30.5 | 16 |
| | 1.83 | 46.3 | 5 |
| PMVK | 1.83 | 22 | 4 |
| DCAKD | 1.83 | 26.5 | 4 |
| | 1.83 | 29.3 | 3 |
| SNX3 | 1.83 | 18.8 | 4 |
| ANAPC7 | 1.82 | 66.8 | 10 |
| FAM133B | 1.82 | 13.1 | 22 |
| ARHGAP17 | 1.82 | 77.5 | 3 |
| SLC33A1 | 1.82 | 60.9 | 2 |
| ELOVL1 | 1.82 | 32.6 | 4 |
| PSMB9 | 1.82 | 20.9 | 5 |
| SF3A1 | 1.82 | 88.8 | 1 |
| CORO7 | 1.82 | 100.5 | 1 |
| MPDZ | 1.82 | 221.5 | 0 |
| STRBP | 1.81 | 72.1 | 7 |
| WDR12 | 1.81 | 47.7 | 13 |
| SRSF7 | 1.81 | 27.4 | 11 |
| SCAMP3 | 1.81 | 38.3 | 9 |
| EIF5AL1 | 1.81 | 16.8 | 8 |
| APBA2 | 1.81 | 27 | 4 |
| | 1.81 | 38.3 | 3 |
| CWC25 | 1.81 | 49.6 | 2 |
| | 1.8 | 30.4 | 13 |
| | 1.8 | 91.4 | 3 |
| RBM22 | 1.8 | 46.9 | 5 |
| ATRX | 1.8 | 282.4 | 1 |
| DHX57 | 1.8 | 155.5 | 2 |
| PPP2R5C | 1.8 | 44.8 | 3 |
| HLTF | 1.8 | 113.9 | 1 |

| | | | |
|---|---|---|---|
| | 1.8 | 25.5 | 4 |
| PHF10 | 1.8 | 56 | 2 |
| HLCS | 1.8 | 80.7 | 1 |
| ATP2B3 | 1.79 | 95.4 | 4 |
| PRPF4 | 1.79 | 58.4 | 8 |
| | 1.79 | 134.6 | 4 |
| PIK3C2A | 1.79 | 190.6 | 1 |
| ABCC1 | 1.79 | 134.8 | 2 |
| RCN2 | 1.79 | 36.9 | 5 |
| | 1.79 | 17.9 | 7 |
| POLR3A | 1.79 | 155.5 | 1 |
| PSMD8 | 1.79 | 39.6 | 2 |
| FBN1 | 1.78 | 147.3 | 6 |
| RPA1 | 1.78 | 68.1 | 13 |
| HS2ST1 | 1.78 | 41.8 | 7 |
| PCOLCE | 1.78 | 47.9 | 7 |
| AKAP8 | 1.78 | 76.1 | 4 |
| | 1.78 | 31.2 | 6 |
| BLVRA | 1.78 | 33.4 | 5 |
| NAA35 | 1.78 | 83.6 | 1 |
| GPSM1 | 1.78 | 74.5 | 1 |
| NEK9 | 1.78 | 57.3 | 2 |
| PI4KA | 1.78 | 96.9 | 2 |
| PRKAA1 | 1.78 | 64 | 2 |
| CCRN4L | 1.78 | 48.2 | 2 |
| LIG3 | 1.77 | 82.3 | 7 |
| ERGIC1 | 1.77 | 32.6 | 13 |
| ATP6V1B2 | 1.77 | 56.5 | 5 |
| AUP1 | 1.77 | 41.6 | 5 |
| RPL21P19 | 1.77 | 18.9 | 16 |
| WDHD1 | 1.77 | 89.5 | 2 |
| ADSL | 1.77 | 56.2 | 2 |
| ALDH9A1 | 1.77 | 53.8 | 3 |
| NDUFA2 | 1.77 | 10.9 | 10 |
| | 1.77 | 26.4 | 3 |
| POR | 1.76 | 64.5 | 9 |
| TFB2M | 1.76 | 27.1 | 11 |
| POLR1C | 1.76 | 17.3 | 13 |
| | 1.76 | 16.1 | 7 |
| AP3M1 | 1.76 | 41 | 2 |
| DDX47 | 1.75 | 50.6 | 8 |
| DRG2 | 1.75 | 38.1 | 15 |
| C1QBP | 1.75 | 31.4 | 5 |
| | 1.75 | 24.9 | 6 |
| HLA-E | 1.75 | 21.1 | 5 |
| PAK1IP1 | 1.75 | 43.9 | 2 |
| | 1.75 | 17.8 | 4 |
| GLG1 | 1.74 | 134.5 | 7 |

| | | | |
|---|---|---|---|
| HIST2H2BE | 1.74 | 13.9 | 29 |
| TARS2 | 1.74 | 81 | 4 |
| ELMO2 | 1.74 | 83.9 | 3 |
| | 1.74 | 31.5 | 7 |
| TBCC | 1.74 | 39.2 | 4 |
| STOML2 | 1.74 | 33.4 | 7 |
| HSPA14 | 1.74 | 54.8 | 2 |
| SACS | 1.74 | 520.8 | 0 |
| MED20 | 1.74 | 15.3 | 8 |
| UTP14A | 1.74 | 87.9 | 1 |
| | 1.74 | 52.3 | 2 |
| PBRM1 | 1.73 | 192.8 | 3 |
| TRMT10C | 1.73 | 47.3 | 7 |
| NOP14 | 1.73 | 97.6 | 4 |
| SNX9 | 1.73 | 39.5 | 8 |
| ARHGEF7 | 1.73 | 81.4 | 2 |
| C21orf33 | 1.73 | 28.1 | 7 |
| SEC24D | 1.73 | 100.2 | 3 |
| | 1.73 | 11 | 25 |
| SNRNP27 | 1.73 | 18.8 | 7 |
| SRC | 1.73 | 59.8 | 3 |
| GLTSCR2 | 1.73 | 54.2 | 4 |
| TRIM22 | 1.73 | 56.9 | 4 |
| | 1.73 | 70 | 2 |
| | 1.73 | 12.7 | 10 |
| NNMT | 1.73 | 29.6 | 4 |
| PHLDA1 | 1.73 | 45 | 2 |
| TOLLIP | 1.73 | 27 | 4 |
| COG1 | 1.73 | 104.7 | 1 |
| | 1.72 | 418.9 | 1 |
| HLA-C | 1.72 | 21.1 | 14 |
| DSP | 1.72 | 265 | 1 |
| | 1.72 | 19.2 | 14 |
| C14orf166 | 1.72 | 28.1 | 7 |
| TRMT1L | 1.72 | 81.7 | 3 |
| ARPC3 | 1.72 | 20.5 | 12 |
| MTOR | 1.72 | 102.9 | 2 |
| | 1.72 | 32.5 | 3 |
| PSMB1 | 1.72 | 26.5 | 4 |
| | 1.72 | 25.4 | 5 |
| SGPP1 | 1.72 | 49.1 | 2 |
| TRMT6 | 1.72 | 55.8 | 2 |
| ICMT | 1.72 | 31.9 | 3 |
| NCKAP1 | 1.71 | 128.7 | 4 |
| | 1.71 | 149.8 | 2 |
| NEU1 | 1.71 | 45.4 | 8 |
| TM9SF4 | 1.71 | 72.5 | 3 |
| TMEM11 | 1.71 | 21.5 | 4 |

| | | | |
|---|---|---|---|
| | 1.7 | 425.8 | 1 |
| MTHFD1L | 1.7 | 99.2 | 5 |
| WDR75 | 1.7 | 92.2 | 5 |
| PLS1 | 1.7 | 70.2 | 6 |
| XAB2 | 1.7 | 99.6 | 2 |
| | 1.7 | 38.2 | 7 |
| PIAS1 | 1.7 | 71.8 | 4 |
| | 1.7 | 108.8 | 2 |
| IMPDH1 | 1.7 | 55.4 | 4 |
| SAMM50 | 1.7 | 51.9 | 4 |
| | 1.7 | 12.9 | 9 |
| LRCH1 | 1.7 | 80.8 | 2 |
| DNAJB12 | 1.7 | 41.8 | 3 |
| CYB5R3 | 1.7 | 16.7 | 7 |
| SP1 | 1.7 | 80.6 | 1 |
| RAB10 | 1.69 | 22.5 | 15 |
| YME1L1 | 1.69 | 57.6 | 6 |
| WWP2 | 1.69 | 98.9 | 3 |
| RAB8A | 1.69 | 23.7 | 9 |
| PSMA3 | 1.69 | 28.4 | 6 |
| ATAD1 | 1.69 | 40.7 | 6 |
| | 1.69 | 10.4 | 15 |
| C12orf23 | 1.69 | 11.7 | 9 |
| | 1.69 | 11.8 | 9 |
| TRIM26 | 1.69 | 62.1 | 2 |
| HMGCS1 | 1.69 | 57.3 | 2 |
| | 1.69 | 35.8 | 3 |
| TANC1 | 1.69 | 152.1 | 1 |
| NLRX1 | 1.69 | 107.5 | 1 |
| GTF3C4 | 1.69 | 62.6 | 2 |
| PAK2 | 1.69 | 58 | 2 |
| | 1.68 | 99.3 | 4 |
| | 1.68 | 73 | 3 |
| EDC4 | 1.68 | 151.6 | 1 |
| TAPBP | 1.68 | 49.5 | 5 |
| EIF1AX | 1.68 | 16.5 | 13 |
| PTPN14 | 1.68 | 135.2 | 2 |
| RPS29 | 1.68 | 6.7 | 29 |
| MRPS6 | 1.68 | 14.2 | 10 |
| | 1.68 | 109.8 | 1 |
| PDP1 | 1.68 | 60.8 | 2 |
| PDPR | 1.68 | 99.3 | 1 |
| RPS13 | 1.67 | 17.2 | 30 |
| TMPO | 1.67 | 12.1 | 27 |
| | 1.67 | 58.6 | 7 |
| | 1.67 | 127.1 | 2 |
| EPB41L1 | 1.67 | 86.4 | 2 |
| | 1.67 | 63 | 4 |

| | | | |
|---|---|---|---|
| H2AFV | 1.67 | 13.5 | 19 |
| TBC1D15 | 1.67 | 79.4 | 3 |
| PEX6 | 1.67 | 104 | 2 |
| TCF25 | 1.67 | 76.6 | 1 |
| RBM10 | 1.67 | 94.3 | 1 |
| VPS29 | 1.67 | 24 | 5 |
| | 1.67 | 12.9 | 7 |
| TEX10 | 1.67 | 90.6 | 1 |
| IGBP1 | 1.67 | 39.2 | 3 |
| NT5DC2 | 1.67 | 60.7 | 2 |
| RBM15B | 1.67 | 97.1 | 1 |
| SLU7 | 1.67 | 68.3 | 1 |
| CDK5RAP3 | 1.66 | 56.9 | 8 |
| PRPF40B | 1.66 | 101.3 | 3 |
| AURKB | 1.66 | 39.3 | 5 |
| WRNIP1 | 1.66 | 66.6 | 2 |
| | 1.66 | 50.5 | 3 |
| CTTN | 1.66 | 61.5 | 4 |
| NAA40 | 1.66 | 27.2 | 4 |
| B3GALT6 | 1.66 | 37.1 | 3 |
| GPX8 | 1.66 | 18.1 | 4 |
| DNM1L | 1.65 | 81.8 | 7 |
| PTK7 | 1.65 | 118.3 | 3 |
| CNP | 1.65 | 45.1 | 8 |
| ZC3HAV1 | 1.65 | 101.4 | 4 |
| AIMP2 | 1.65 | 35.3 | 9 |
| GYS2 | 1.65 | 80.9 | 3 |
| | 1.65 | 150.4 | 2 |
| USP16 | 1.65 | 93.5 | 4 |
| SLC2A1 | 1.65 | 57 | 5 |
| ZNF277 | 1.65 | 33.9 | 5 |
| KIAA1033 | 1.65 | 136.3 | 2 |
| GTF2E2 | 1.65 | 33 | 4 |
| RBMS1 | 1.65 | 44.1 | 4 |
| TNS3 | 1.64 | 95.8 | 4 |
| | 1.64 | 43.5 | 11 |
| | 1.64 | 33.7 | 8 |
| TSFM | 1.64 | 31 | 11 |
| NKIRAS2 | 1.64 | 21.5 | 12 |
| DROSHA | 1.64 | 156.2 | 1 |
| | 1.64 | 9.6 | 13 |
| | 1.64 | 34.6 | 3 |
| | 1.64 | 146.4 | 1 |
| | 1.64 | 14.5 | 7 |
| ANXA6 | 1.64 | 75.8 | 1 |
| TMEM167A | 1.64 | 8.1 | 13 |
| LEPRE1 | 1.63 | 83.3 | 16 |
| LRP1 | 1.63 | 504.3 | 2 |

| | | | |
|---|---|---|---|
| PRDX6 | 1.63 | 25 | 19 |
| EIF2B3 | 1.63 | 50.2 | 8 |
| NQO1 | 1.63 | 22.8 | 15 |
| SNX4 | 1.63 | 20.7 | 12 |
| | 1.63 | 36.4 | 5 |
| | 1.63 | 9.5 | 25 |
| NUP35 | 1.63 | 34.8 | 4 |
| CNOT10 | 1.63 | 42.2 | 3 |
| ARL6IP6 | 1.63 | 24.7 | 5 |
| LSM6 | 1.63 | 9.1 | 9 |
| ABCB6 | 1.63 | 93.8 | 1 |
| POLR2C | 1.63 | 31.4 | 3 |
| TSGA13 | 1.63 | 31.8 | 3 |
| | 1.62 | 39.2 | 17 |
| FECH | 1.62 | 47.1 | 11 |
| PRKCI | 1.62 | 67.2 | 4 |
| TAGLN2 | 1.62 | 21.1 | 6 |
| SPCS3 | 1.62 | 20.3 | 7 |
| PIP | 1.62 | 16.6 | 10 |
| SYNJ2BP | 1.62 | 20.5 | 5 |
| | 1.62 | 10.8 | 8 |
| DYM | 1.62 | 75.9 | 1 |
| HEATR1 | 1.61 | 242.1 | 2 |
| ANLN | 1.61 | 110.8 | 2 |
| RAPGEF6 | 1.61 | 180.4 | 1 |
| FHL1 | 1.61 | 29.1 | 5 |
| | 1.61 | 18.4 | 7 |
| PTPRK | 1.61 | 162 | 1 |
| HLA-C | 1.61 | 26.4 | 5 |
| IKBIP | 1.61 | 39.3 | 3 |
| NUMB | 1.61 | 65.9 | 1 |
| SH3PXD2A | 1.61 | 125.2 | 1 |
| | 1.6 | 93.9 | 9 |
| DHX38 | 1.6 | 140.4 | 2 |
| TOE1 | 1.6 | 25.8 | 21 |
| SEC22B | 1.6 | 24.6 | 13 |
| IMP3 | 1.6 | 21.8 | 8 |
| MEX3D | 1.6 | 64.8 | 1 |
| DDB1 | 0 | 126.9 | 7 |
| | 0 | 101.6 | 8 |
| | 0 | 174 | 3 |
| HSPB1 | 0 | 22.8 | 24 |
| ATL3 | 0 | 60.5 | 10 |
| WDR74 | 0 | 42.4 | 18 |
| PLRG1 | 0 | 57.2 | 14 |
| CTR9 | 0 | 133.4 | 4 |
| HUWE1 | 0 | 374 | 2 |
| COPG2 | 0 | 97.6 | 5 |

| | | | |
|---|---|---|---|
| RAI14 | 0 | 74.5 | 7 |
| MAP2K2 | 0 | 44.4 | 8 |
| PPP2R1A | 0 | 65.3 | 9 |
| TCERG1 | 0 | 116.1 | 4 |
| HIST2H2BF | 0 | 13.9 | 29 |
| PLCB3 | 0 | 138.7 | 6 |
| | 0 | 51.9 | 13 |
| SIPA1L2 | 0 | 165.6 | 3 |
| SIN3A | 0 | 145.1 | 3 |
| RBM4B | 0 | 40.1 | 11 |
| ZCCHC6 | 0 | 171.1 | 3 |
| SPTLC2 | 0 | 62.9 | 7 |
| | 0 | 28.9 | 12 |
| | 0 | 77.9 | 5 |
| EPB41L2 | 0 | 104.3 | 4 |
| IARS2 | 0 | 105.9 | 4 |
| GNL2 | 0 | 83.6 | 5 |
| SNX18 | 0 | 68.9 | 7 |
| DNTTIP2 | 0 | 84.4 | 6 |
| YWHAG | 0 | 23.5 | 15 |
| BRIX1 | 0 | 15.3 | 25 |
| | 0 | 25.3 | 17 |
| RPL27 | 0 | 15.8 | 32 |
| AAAS | 0 | 59.5 | 10 |
| TBC1D4 | 0 | 146.5 | 4 |
| PKN2 | 0 | 112 | 3 |
| GNAO1 | 0 | 40.2 | 8 |
| GPATCH8 | 0 | 164.1 | 2 |
| AHCY | 0 | 33.8 | 14 |
| MDN1 | 0 | 632.4 | 1 |
| ARL8B | 0 | 27.2 | 12 |
| KIAA0368 | 0 | 223.6 | 1 |
| ASMTL | 0 | 68.8 | 5 |
| GLS | 0 | 37.4 | 13 |
| SRBD1 | 0 | 111.7 | 3 |
| DVL2 | 0 | 78.9 | 5 |
| RPP30 | 0 | 29.3 | 15 |
| | 0 | 73 | 6 |
| SEC23A | 0 | 82.9 | 7 |
| POGZ | 0 | 86 | 5 |
| CTSB | 0 | 37.8 | 13 |
| LRRC59 | 0 | 34.9 | 10 |
| | 0 | 51.2 | 6 |
| | 0 | 80.7 | 5 |
| UMPS | 0 | 52.2 | 6 |
| CHMP4B | 0 | 24.9 | 14 |
| ATP5H | 0 | 18.5 | 12 |
| POP1 | 0 | 114.6 | 3 |

| | | | |
|---|---|---|---|
| DCAF13 | 0 | 67.5 | 3 |
| PGK1 | 0 | 44.6 | 6 |
| | 0 | 18.7 | 26 |
| BAZ1A | 0 | 178.6 | 2 |
| UBE4B | 0 | 121.3 | 3 |
| | 0 | 34.9 | 10 |
| IVNS1ABP | 0 | 71.7 | 5 |
| | 0 | 15.2 | 26 |
| IQSEC1 | 0 | 124.3 | 3 |
| | 0 | 47.1 | 6 |
| ITPR2 | 0 | 307.9 | 1 |
| RPS10 | 0 | 18.9 | 10 |
| | 0 | 24.9 | 10 |
| DTYMK | 0 | 23.7 | 14 |
| AGPAT4 | 0 | 44 | 7 |
| EEF1B2 | 0 | 24.7 | 14 |
| RTF1 | 0 | 80.3 | 5 |
| SMARCC2 | 0 | 132.8 | 2 |
| HSD17B10 | 0 | 26.9 | 12 |
| WDR77 | 0 | 36.7 | 6 |
| POLD1 | 0 | 111.8 | 2 |
| YTHDC1 | 0 | 85.5 | 3 |
| WDR26 | 0 | 72.1 | 3 |
| BAZ2A | 0 | 207.9 | 1 |
| | 0 | 16.9 | 17 |
| MPHOSPH10 | 0 | 76.3 | 4 |
| | 0 | 26.7 | 8 |
| TRIP10 | 0 | 68.3 | 6 |
| SCYL1 | 0 | 89.6 | 3 |
| EHD2 | 0 | 60 | 6 |
| ARPC5L | 0 | 16.9 | 18 |
| ATXN10 | 0 | 53.5 | 5 |
| | 0 | 43.8 | 5 |
| | 0 | 509.8 | 1 |
| MMP14 | 0 | 65.9 | 4 |
| FCF1 | 0 | 23.4 | 10 |
| VDAC3 | 0 | 30.6 | 12 |
| LARP4 | 0 | 80.5 | 2 |
| RNMT | 0 | 57.7 | 4 |
| CPSF3 | 0 | 77.4 | 4 |
| TRIP13 | 0 | 48.5 | 6 |
| KPNA4 | 0 | 57.9 | 5 |
| MYH10 | 0 | 5.4 | 54 |
| MAD1L1 | 0 | 83 | 4 |
| LSG1 | 0 | 75.2 | 5 |
| G3BP2 | 0 | 50.8 | 11 |
| ALYREF | 0 | 27.5 | 4 |
| TMX3 | 0 | 48.5 | 6 |

| | | | |
|---|---|---|---|
| XPOT | 0 | 109.8 | 2 |
| PRMT1 | 0 | 37.5 | 11 |
| | 0 | 262.5 | 1 |
| TAOK3 | 0 | 105.3 | 3 |
| PTPN12 | 0 | 88.1 | 3 |
| MID1 | 0 | 69.4 | 7 |
| STK38 | 0 | 54.2 | 7 |
| MICAL1 | 0 | 116.8 | 2 |
| ZYX | 0 | 55.4 | 5 |
| USP5 | 0 | 95.7 | 3 |
| PSMA5 | 0 | 26.4 | 13 |
| | 0 | 84.7 | 3 |
| MAPRE1 | 0 | 26.6 | 7 |
| DYNC1LI2 | 0 | 54.1 | 4 |
| | 0 | 40.7 | 11 |
| EXOC2 | 0 | 104.1 | 2 |
| | 0 | 55.1 | 5 |
| UAP1L1 | 0 | 57 | 3 |
| | 0 | 36.4 | 8 |
| PTRH2 | 0 | 19.3 | 8 |
| KDM2A | 0 | 132.7 | 2 |
| NNT | 0 | 113.8 | 2 |
| | 0 | 15.8 | 13 |
| | 0 | 61.2 | 4 |
| IFIT5 | 0 | 55.8 | 5 |
| VPS36 | 0 | 43.8 | 6 |
| SF1 | 0 | 67.3 | 3 |
| COPE | 0 | 34.4 | 13 |
| RDH11 | 0 | 35.4 | 5 |
| PSIP1 | 0 | 60.1 | 4 |
| | 0 | 17.6 | 18 |
| CWC22 | 0 | 105.4 | 2 |
| MAD2L1 | 0 | 23.5 | 8 |
| METTL13 | 0 | 78.6 | 3 |
| | 0 | 16.1 | 16 |
| NAA10 | 0 | 26.4 | 9 |
| CDC27 | 0 | 84.8 | 3 |
| CTNNBL1 | 0 | 65.1 | 3 |
| | 0 | 77 | 4 |
| RCN1 | 0 | 38.9 | 6 |
| | 0 | 19.2 | 15 |
| USP40 | 0 | 140 | 2 |
| PRKCSH | 0 | 45.9 | 5 |
| HDAC6 | 0 | 130.3 | 2 |
| FAM111B | 0 | 84.6 | 3 |
| PRKAR2A | 0 | 45.5 | 4 |
| TBC1D9B | 0 | 140.4 | 2 |
| ATXN2L | 0 | 103.5 | 3 |

| | | | |
|---|---|---|---|
| YIPF5 | 0 | 28 | 8 |
| UBE2O | 0 | 141.2 | 2 |
| CHAMP1 | 0 | 89 | 2 |
| BBX | 0 | 105.1 | 2 |
| | 0 | 47.1 | 4 |
| PSMD11 | 0 | 35.1 | 6 |
| PDCD6IP | 0 | 96 | 2 |
| NUFIP2 | 0 | 76.1 | 3 |
| SRSF10 | 0 | 31.3 | 7 |
| ARAP1 | 0 | 162.1 | 2 |
| FAM115A | 0 | 102.1 | 2 |
| | 0 | 31.9 | 9 |
| ALDH6A1 | 0 | 57.8 | 3 |
| WNK1 | 0 | 222.6 | 1 |
| PLOD3 | 0 | 84.7 | 2 |
| NOM1 | 0 | 96.2 | 3 |
| YTHDF2 | 0 | 61.3 | 6 |
| PSMA6 | 0 | 6.2 | 19 |
| UBE3A | 0 | 100.6 | 3 |
| LAMB1 | 0 | 197.9 | 1 |
| | 0 | 14.3 | 23 |
| LARP1 | 0 | 123.4 | 2 |
| RICTOR | 0 | 192.1 | 1 |
| | 0 | 52.4 | 5 |
| CHPF | 0 | 85.4 | 2 |
| PTPN13 | 0 | 276.7 | 1 |
| SHCBP1 | 0 | 75.6 | 3 |
| RAC2 | 0 | 21.4 | 10 |
| | 0 | 57.4 | 4 |
| MAP3K4 | 0 | 181.1 | 1 |
| ITGA3 | 0 | 116.5 | 2 |
| ALG1 | 0 | 52.5 | 4 |
| MARCKS | 0 | 14.9 | 24 |
| CCDC12 | 0 | 19.2 | 12 |
| | 0 | 47.2 | 4 |
| PGRMC1 | 0 | 21.7 | 9 |
| NDUFS3 | 0 | 30.2 | 5 |
| PRKD3 | 0 | 100.4 | 2 |
| RANBP6 | 0 | 124.6 | 2 |
| | 0 | 197 | 1 |
| FAM91A1 | 0 | 88.9 | 2 |
| NFIC | 0 | 54.6 | 5 |
| LAMB3 | 0 | 129.5 | 2 |
| LMF2 | 0 | 79.6 | 2 |
| | 0 | 15.9 | 16 |
| EHMT2 | 0 | 114.9 | 2 |
| PPIL4 | 0 | 57.2 | 4 |
| PACSIN2 | 0 | 50 | 5 |

| | | | |
|---|---|---|---|
| MPP6 | 0 | 61.1 | 4 |
| | 0 | 73.7 | 4 |
| DCAF7 | 0 | 38.9 | 5 |
| | 0 | 17.2 | 12 |
| KDM3B | 0 | 191.5 | 1 |
| PLXNB2 | 0 | 205 | 1 |
| ACTR10 | 0 | 46.3 | 4 |
| ABI1 | 0 | 55 | 2 |
| RRP7B | 0 | 12.6 | 15 |
| ENY2 | 0 | 11.5 | 16 |
| POLDIP2 | 0 | 42 | 8 |
| HDAC1 | 0 | 27.3 | 8 |
| PARK7 | 0 | 17.9 | 13 |
| LEPREL2 | 0 | 81.8 | 2 |
| SLC25A1 | 0 | 35 | 7 |
| PQBP1 | 0 | 32.2 | 10 |
| ARF5 | 0 | 25.4 | 9 |
| DNAJC11 | 0 | 58.8 | 3 |
| PPM1G | 0 | 57.4 | 5 |
| NDC1 | 0 | 76.3 | 2 |
| TXNL1 | 0 | 32.2 | 10 |
| STK38L | 0 | 54 | 4 |
| CMTR1 | 0 | 95.3 | 3 |
| GNG12 | 0 | 8 | 15 |
| NUB1 | 0 | 70.5 | 2 |
| KHDRBS3 | 0 | 38.8 | 5 |
| PKP4 | 0 | 131.8 | 1 |
| TMEM97 | 0 | 20.8 | 10 |
| PDLIM1 | 0 | 36 | 7 |
| MAGEA2B | 0 | 35 | 7 |
| WDR37 | 0 | 54.6 | 3 |
| | 0 | 123.8 | 2 |
| MRPL18 | 0 | 20.4 | 12 |
| INTS4 | 0 | 108.1 | 2 |
| HRNR | 0 | 282.2 | 3 |
| MOGS | 0 | 62.2 | 4 |
| FKBP8 | 0 | 38.5 | 5 |
| SEC24A | 0 | 119.7 | 1 |
| GGCX | 0 | 87.5 | 2 |
| TBCD | 0 | 136.5 | 1 |
| NFXL1 | 0 | 101.2 | 1 |
| PARP10 | 0 | 109.9 | 1 |
| DPYSL4 | 0 | 61.8 | 2 |
| GAK | 0 | 143.1 | 1 |
| MLEC | 0 | 32.2 | 4 |
| KIF4A | 0 | 60.9 | 2 |
| EMC8 | 0 | 23.8 | 6 |
| DVL3 | 0 | 78 | 3 |

| | | | |
|---|---|---|---|
| PLK1 | 0 | 41.5 | 5 |
| ATP1B1 | 0 | 35 | 9 |
| | 0 | 164.8 | 1 |
| IFITM3 | 0 | 14.6 | 12 |
| DGKA | 0 | 82.6 | 3 |
| NAMPT | 0 | 26.8 | 7 |
| CHPF2 | 0 | 85.9 | 2 |
| TAX1BP3 | 0 | 13.7 | 14 |
| | 0 | 11.1 | 16 |
| ORC3 | 0 | 75 | 3 |
| | 0 | 17.4 | 12 |
| LRRC42 | 0 | 48.5 | 2 |
| NOC3L | 0 | 92.5 | 2 |
| | 0 | 29.6 | 3 |
| | 0 | 21.4 | 18 |
| GBF1 | 0 | 206.3 | 1 |
| XIAP | 0 | 56.6 | 2 |
| ARPC5 | 0 | 16.3 | 10 |
| | 0 | 17 | 15 |
| LRCH3 | 0 | 89.1 | 2 |
| | 0 | 17.3 | 7 |
| EMC3 | 0 | 29.9 | 5 |
| MRPL21 | 0 | 22.8 | 9 |
| NFX1 | 0 | 124.3 | 2 |
| | 0 | 21.3 | 5 |
| STIP1 | 0 | 62.6 | 3 |
| RRAGC | 0 | 44.2 | 4 |
| GATAD2B | 0 | 65.2 | 2 |
| | 0 | 36 | 5 |
| TIMP1 | 0 | 23.2 | 5 |
| NES | 0 | 177.3 | 1 |
| EIF2D | 0 | 64.7 | 3 |
| ARFGAP2 | 0 | 42.1 | 3 |
| MKRN2 | 0 | 46.9 | 2 |
| NRBP1 | 0 | 59.8 | 3 |
| ERCC4 | 0 | 52.8 | 3 |
| | 0 | 43.5 | 3 |
| ADH5 | 0 | 39.7 | 4 |
| | 0 | 16.2 | 9 |
| RBCK1 | 0 | 57.5 | 6 |
| | 0 | 217.2 | 1 |
| SNRPD1 | 0 | 13.3 | 11 |
| UGP2 | 0 | 57.8 | 3 |
| EXOC1 | 0 | 100.2 | 1 |
| MGEA5 | 0 | 54.2 | 3 |
| LAS1L | 0 | 83 | 2 |
| | 0 | 92.9 | 1 |
| RCOR3 | 0 | 55.5 | 2 |

| | | | |
|---|---|---|---|
| RRAS2 | 0 | 23.4 | 6 |
| PAPSS1 | 0 | 70.8 | 2 |
| UBAC2 | 0 | 38.9 | 4 |
| | 0 | 76.3 | 2 |
| NDUFB1 | 0 | 10 | 12 |
| | 0 | 16.9 | 7 |
| RALGAPA2 | 0 | 210.6 | 1 |
| | 0 | 13.6 | 9 |
| GLIPR1 | 0 | 30.3 | 4 |
| UPF2 | 0 | 34 | 3 |
| RETSAT | 0 | 66.8 | 2 |
| FBXO30 | 0 | 82.3 | 2 |
| | 0 | 59.6 | 2 |
| | 0 | 83.2 | 3 |
| EIF3K | 0 | 25 | 5 |
| MRPL54 | 0 | 15.8 | 7 |
| | 0 | 21 | 4 |
| CTPS2 | 0 | 65.6 | 2 |
| FASTKD2 | 0 | 81.4 | 1 |
| RER1 | 0 | 13.4 | 17 |
| | 0 | 48.5 | 2 |
| TMED1 | 0 | 25.2 | 7 |
| RNF40 | 0 | 113.6 | 1 |
| BANF1 | 0 | 10.1 | 16 |
| | 0 | 67.1 | 2 |
| | 0 | 61.9 | 2 |
| METTL14 | 0 | 52.1 | 2 |
| C16orf88 | 0 | 51.6 | 3 |
| GOLT1B | 0 | 15.4 | 7 |
| SLC27A1 | 0 | 71.1 | 1 |
| DENND4C | 0 | 123.1 | 1 |
| GTPBP2 | 0 | 65.7 | 2 |
| CDC23 | 0 | 68.8 | 3 |
| | 0 | 21.1 | 8 |
| LPP | 0 | 65.7 | 1 |
| | 0 | 13.1 | 12 |
| STRIP1 | 0 | 51.6 | 2 |
| LYPLA2 | 0 | 17.6 | 5 |
| PRDX3 | 0 | 27.7 | 5 |
| SELO | 0 | 57.2 | 2 |
| LCLAT1 | 0 | 38.7 | 3 |
| CPSF3L | 0 | 67.6 | 2 |
| CISD1 | 0 | 12.2 | 12 |
| | 0 | 25.8 | 3 |
| PRMT3 | 0 | 61.9 | 2 |
| PDPK1 | 0 | 63.1 | 1 |
| ARF3 | 0 | 20.6 | 14 |
| ARHGEF6 | 0 | 34.8 | 3 |

| | | | |
|---|---|---|---|
| POLR3D | 0 | 44.4 | 2 |
| MAPKAPK5 | 0 | 54.2 | 2 |
| NSUN6 | 0 | 51.7 | 2 |
| EML3 | 0 | 95.1 | 2 |
| GOPC | 0 | 50.5 | 2 |
| SETX | 0 | 93.3 | 1 |
| UBE4A | 0 | 119.7 | 1 |
| VPS16 | 0 | 94.6 | 2 |
| PPID | 0 | 40.7 | 2 |
| TRIM27 | 0 | 58.5 | 2 |
| DPY19L1 | 0 | 84.5 | 2 |
| | 0 | 11.6 | 13 |
| FIZ1 | 0 | 52 | 2 |
| PA2G4 | 0 | 40.9 | 2 |
| APEX1 | 0 | 35.5 | 3 |
| TMED5 | 0 | 26 | 4 |
| RIOK2 | 0 | 63.2 | 2 |
| QTRTD1 | 0 | 46.7 | 2 |
| TMED7 | 0 | 21.2 | 5 |
| DLG5 | 0 | 96.6 | 2 |
| PARP4 | 0 | 192.5 | 1 |
| | 0 | 13.4 | 9 |
| | 0 | 38.7 | 3 |
| | 0 | 42.5 | 2 |
| KIF20A | 0 | 100.2 | 3 |
| | 0 | 45.6 | 2 |
| MPG | 0 | 27.3 | 4 |
| API5 | 0 | 59 | 2 |
| | 0 | 25.7 | 3 |
| POLR2L | 0 | 7.6 | 13 |
| GJA1 | 0 | 43 | 2 |
| KIAA1217 | 0 | 214 | 1 |
| ADAM10 | 0 | 57.9 | 2 |
| DSG1 | 0 | 113.7 | 1 |
| IDI1 | 0 | 26.3 | 5 |
| MRPL11 | 0 | 20.6 | 5 |
| AP3S2 | 0 | 23.4 | 5 |
| EIF4E | 0 | 25.1 | 4 |
| SLX4 | 0 | 199.9 | 1 |
| | 0 | 4.9 | 24 |
| EXOSC3 | 0 | 29.6 | 4 |
| | 0 | 21.6 | 4 |
| MRPL40 | 0 | 24.5 | 4 |
| CDC40 | 0 | 56.7 | 4 |
| | 0 | 37.3 | 4 |
| MX2 | 0 | 82 | 1 |
| GEMIN4 | 0 | 120 | 1 |
| ANKRD26 | 0 | 198.1 | 1 |

| | | | |
|---|---|---|---|
| NUBP1 | 0 | 34.5 | 3 |
| ZNF512B | 0 | 97.2 | 1 |
| XPNPEP1 | 0 | 69.9 | 2 |
| | 0 | 14.4 | 8 |
| PICALM | 0 | 70.7 | 1 |
| CSRP2 | 0 | 26.7 | 5 |
| ECI2 | 0 | 40.2 | 2 |
| SFMBT2 | 0 | 100.5 | 1 |
| OGFR | 0 | 73.3 | 3 |
| IGHMBP2 | 0 | 109.1 | 1 |
| TMEM33 | 0 | 27.9 | 3 |
| SULT1A4 | 0 | 34.2 | 2 |
| CLK3 | 0 | 22.4 | 4 |
| ECD | 0 | 72.7 | 1 |
| PDCD2 | 0 | 38.6 | 3 |
| CYR61 | 0 | 42 | 2 |
| USP47 | 0 | 157.2 | 1 |
| GMPS | 0 | 76.7 | 1 |
| ZNF24 | 0 | 42.1 | 4 |
| | 0 | 31.8 | 7 |
| TLE3 | 0 | 46 | 3 |
| TRIP6 | 0 | 27.6 | 5 |
| NDUFA11 | 0 | 14.8 | 11 |
| BAG2 | 0 | 23.8 | 4 |
| RPL35A | 0 | 12.5 | 11 |
| NDUFA8 | 0 | 20.1 | 5 |
| SMAD5 | 0 | 52.2 | 2 |
| | 0 | 15.6 | 6 |
| CLK2 | 0 | 32.3 | 3 |
| LEO1 | 0 | 75.4 | 2 |
| ITM2C | 0 | 23.9 | 4 |
| NFS1 | 0 | 28.2 | 5 |
| ACBD3 | 0 | 60.6 | 3 |
| PTDSS1 | 0 | 55.4 | 2 |
| PGM2L1 | 0 | 70.4 | 2 |
| MAEA | 0 | 45.3 | 2 |
| CERS6 | 0 | 44.9 | 2 |
| MLST8 | 0 | 35.9 | 6 |
| IRF2BP1 | 0 | 61.6 | 2 |
| AP1S2 | 0 | 15.5 | 7 |
| REXO4 | 0 | 26.5 | 3 |
| | 0 | 9 | 20 |
| GSTK1 | 0 | 21.7 | 7 |
| QPCTL | 0 | 42.9 | 3 |
| INTS1 | 0 | 244.1 | 0 |
| PNPT1 | 0 | 85.9 | 2 |
| | 0 | 28.1 | 5 |
| | 0 | 14.6 | 8 |

| | | | |
|---|---|---|---|
| FRMD6 | 0 | 72 | 2 |
| | 0 | 38.4 | 3 |
| ABCB10 | 0 | 79.1 | 2 |
| ARL6IP5 | 0 | 21.5 | 4 |
| EMG1 | 0 | 26.7 | 5 |
| | 0 | 24.5 | 5 |
| SLC38A10 | 0 | 119.7 | 1 |
| TCEB3 | 0 | 87.2 | 2 |
| | 0 | 114.1 | 1 |
| FAS | 0 | 12.4 | 11 |
| TDRD3 | 0 | 73.1 | 1 |
| | 0 | 51.8 | 1 |
| RSU1 | 0 | 25.7 | 4 |
| DNAJC1 | 0 | 25 | 4 |
| COPS7A | 0 | 27.3 | 4 |
| | 0 | 36.3 | 6 |
| DSCR3 | 0 | 24.2 | 4 |
| PAFAH1B1 | 0 | 27.1 | 6 |
| STXBP3 | 0 | 67.7 | 2 |
| | 0 | 87.4 | 1 |
| MTG1 | 0 | 32.6 | 4 |
| | 0 | 19.7 | 5 |
| | 0 | 23.3 | 4 |
| TMED9 | 0 | 13.7 | 6 |
| TMEM106B | 0 | 35.1 | 3 |
| WDR1 | 0 | 66.1 | 1 |
| | 0 | 139.9 | 1 |
| PDHX | 0 | 54.1 | 2 |
| ANKRD11 | 0 | 25.3 | 3 |
| | 0 | 4.8 | 27 |
| ATP5J2 | 0 | 10.9 | 15 |
| MRTO4 | 0 | 27.5 | 5 |
| FARP1 | 0 | 122 | 1 |
| TNPO3 | 0 | 96.6 | 1 |
| TRMT2A | 0 | 68.7 | 1 |
| PANK4 | 0 | 86.9 | 2 |
| CREB1 | 0 | 35.1 | 3 |
| SCD5 | 0 | 37.6 | 2 |
| PIK3C3 | 0 | 94.3 | 2 |
| | 0 | 27.7 | 3 |
| TMEM214 | 0 | 75.4 | 1 |
| RBFOX3 | 0 | 33.9 | 3 |
| | 0 | 42.8 | 3 |
| COPS2 | 0 | 51.6 | 2 |
| EEFSEC | 0 | 65.3 | 2 |
| COLGALT1 | 0 | 26 | 3 |
| ANKZF1 | 0 | 57.5 | 3 |
| SMARCE1 | 0 | 17.6 | 7 |

| | | | |
|---|---|---|---|
| RPL22L1 | 0 | 14.5 | 10 |
| TFG | 0 | 43.4 | 3 |
| | 0 | 21.2 | 8 |
| | 0 | 31.4 | 3 |
| | 0 | 15.7 | 6 |
| | 0 | 18.2 | 6 |
| CNOT2 | 0 | 59.7 | 2 |
| PRKAR2B | 0 | 44.8 | 3 |
| NDUFA4 | 0 | 9.4 | 12 |
| PM20D2 | 0 | 47.7 | 3 |
| ARHGAP5 | 0 | 172.4 | 1 |
| | 0 | 210.9 | 1 |
| ANAPC1 | 0 | 216.4 | 0 |
| ETF1 | 0 | 47.4 | 3 |
| CDC16 | 0 | 71.6 | 1 |
| | 0 | 26.5 | 4 |
| SLC25A20 | 0 | 27.7 | 5 |
| SSU72 | 0 | 22.6 | 6 |
| UTRN | 0 | 394.2 | 0 |
| ANXA7 | 0 | 37.8 | 5 |
| C15orf52 | 0 | 57.3 | 2 |
| INTS7 | 0 | 106.8 | 1 |
| CSTF3 | 0 | 82.9 | 2 |
| SUN2 | 0 | 25.4 | 4 |
| MRPL1 | 0 | 36.9 | 4 |
| RAB34 | 0 | 30.6 | 5 |
| | 0 | 82.1 | 2 |
| | 0 | 4.1 | 23 |
| GALNT2 | 0 | 64.7 | 2 |
| ZC3H13 | 0 | 196.5 | 1 |
| MEF2C | 0 | 51.2 | 2 |
| ITGAV | 0 | 116 | 1 |
| | 0 | 62.2 | 2 |
| ZBTB11 | 0 | 119.3 | 1 |
| | 0 | 10.3 | 12 |
| | 0 | 7.7 | 11 |
| COG4 | 0 | 89.5 | 2 |
| ITGA1 | 0 | 130.8 | 1 |
| | 0 | 23.1 | 6 |
| TMPO | 0 | 75.4 | 1 |
| ITGA2 | 0 | 129.2 | 1 |
| GDAP1 | 0 | 28.8 | 3 |
| CHURC1 | 0 | 16.1 | 9 |
| SRSF11 | 0 | 42.3 | 4 |
| | 0 | 13.3 | 13 |
| MOB4 | 0 | 26 | 4 |
| | 0 | 5.1 | 15 |
| CARM1 | 0 | 65.8 | 1 |

| | | | |
|---|---|---|---|
| LIMCH1 | 0 | 94.1 | 1 |
| | 0 | 82.9 | 2 |
| | 0 | 31.2 | 2 |
| SPCS1 | 0 | 18.3 | 10 |
| | 0 | 30.2 | 3 |
| PEX1 | 0 | 136.5 | 1 |
| MOCS1 | 0 | 43.1 | 2 |
| UPF3B | 0 | 57.7 | 2 |
| PSD3 | 0 | 108.9 | 1 |
| MRPL38 | 0 | 44.6 | 3 |
| TBC1D2 | 0 | 105.3 | 1 |
| | 0 | 15.2 | 8 |
| SBNO1 | 0 | 154.2 | 1 |
| MAGED2 | 0 | 63.1 | 1 |
| | 0 | 8.7 | 8 |
| | 0 | 126.2 | 1 |
| AGTPBP1 | 0 | 138.4 | 1 |
| | 0 | 236 | 0 |
| TPP1 | 0 | 53.8 | 3 |
| PCYT2 | 0 | 41.4 | 4 |
| GTF3C2 | 0 | 100.6 | 1 |
| SNX1 | 0 | 59 | 2 |
| ACAD9 | 0 | 55 | 2 |
| | 0 | 13.8 | 11 |
| MAN1B1 | 0 | 41.7 | 6 |
| | 0 | 3.5 | 31 |
| PPAT | 0 | 24.5 | 5 |
| PIK3R4 | 0 | 153.1 | 1 |
| UBE2Z | 0 | 38.2 | 2 |
| CADPS | 0 | 25.6 | 3 |
| KPRP | 0 | 64.1 | 1 |
| NDUFS4 | 0 | 20.1 | 9 |
| | 0 | 37.6 | 3 |
| MRC2 | 0 | 166.6 | 1 |
| VCPIP1 | 0 | 134.2 | 1 |
| | 0 | 11.6 | 7 |
| UTP15 | 0 | 58.4 | 2 |
| MAP7D3 | 0 | 98.4 | 1 |
| CLPTM1L | 0 | 43.3 | 4 |
| CENPE | 0 | 316.2 | 0 |
| RFC5 | 0 | 38.4 | 2 |
| ATP5D | 0 | 17.5 | 8 |
| ATG16L1 | 0 | 64.9 | 2 |
| VAMP5 | 0 | 12.8 | 6 |
| DDX52 | 0 | 46.1 | 2 |
| | 0 | 37.3 | 3 |
| GRSF1 | 0 | 36.6 | 3 |
| | 0 | 17.6 | 7 |

| | | | |
|---|---|---|---|
| | 0 | 48.5 | 3 |
| RAB6B | 0 | 23.4 | 5 |
| XRCC1 | 0 | 69.5 | 2 |
| APOBEC3G | 0 | 46.5 | 2 |
| RNF138 | 0 | 28.2 | 4 |
| | 0 | 317.6 | 0 |
| CASK | 0 | 105.1 | 1 |
| CCNT1 | 0 | 80.5 | 2 |
| ELP3 | 0 | 52.1 | 2 |
| | 0 | 48.1 | 2 |
| STAT3 | 0 | 88 | 2 |
| XIRP1 | 0 | 198.4 | 1 |
| TRAPPC9 | 0 | 128.4 | 1 |
| APEH | 0 | 81.2 | 2 |
| ZNF460 | 0 | 63.7 | 1 |
| RANBP9 | 0 | 77.8 | 2 |
| | 0 | 16.5 | 5 |
| | 0 | 13.1 | 13 |
| SCCPDH | 0 | 47.1 | 3 |
| C7orf50 | 0 | 22.1 | 7 |
| NHLRC2 | 0 | 27 | 4 |
| ZNF471 | 0 | 73 | 4 |
| | 0 | 68.4 | 1 |
| | 0 | 13.2 | 8 |
| MAT1A | 0 | 43.6 | 2 |
| | 0 | 80.8 | 2 |
| TOP3A | 0 | 112.3 | 1 |
| AATF | 0 | 63.1 | 1 |
| | 0 | 26.2 | 4 |
| INTS2 | 0 | 133.3 | 1 |
| DIP2B | 0 | 171.4 | 1 |
| | 0 | 17.9 | 5 |
| WNT4 | 0 | 6 | 25 |
| RPTOR | 0 | 148.9 | 1 |
| ACSL5 | 0 | 75.9 | 2 |
| FAM129A | 0 | 103.1 | 2 |
| | 0 | 12.9 | 13 |
| S100A10 | 0 | 22.3 | 8 |
| | 0 | 17.3 | 6 |
| FOXK1 | 0 | 75.4 | 1 |
| NAA30 | 0 | 27.1 | 5 |
| CDC20 | 0 | 54.7 | 2 |
| RBM12 | 0 | 76.4 | 1 |
| | 0 | 38 | 3 |
| | 0 | 25.1 | 4 |
| CYC1 | 0 | 34.5 | 3 |
| SMN1 | 0 | 24.4 | 4 |
| | 0 | 69 | 1 |

| | | | |
|---|---|---|---|
| PRRC1 | 0 | 46.7 | 2 |
| | 0 | 38.1 | 4 |
| ACOT1 | 0 | 46.2 | 3 |
| | 0 | 121.2 | 1 |
| ATL1 | 0 | 54.1 | 2 |
| | 0 | 35.7 | 2 |
| WDR61 | 0 | 33.6 | 2 |
| BCR | 0 | 105.5 | 1 |
| | 0 | 15.9 | 7 |
| | 0 | 26.8 | 3 |
| TUBGCP3 | 0 | 103.5 | 1 |
| ITIH1 | 0 | 69.5 | 1 |
| MFN1 | 0 | 84.1 | 1 |
| GFM1 | 0 | 16.7 | 4 |
| RABGAP1 | 0 | 121.7 | 1 |
| | 0 | 108.5 | 1 |
| EIF3D | 0 | 63.9 | 1 |
| PSME2 | 0 | 27.3 | 5 |
| | 0 | 13 | 13 |
| QKI | 0 | 37.6 | 5 |
| | 0 | 18 | 6 |
| | 0 | 13.6 | 10 |
| CARS | 0 | 84.2 | 1 |
| | 0 | 25.5 | 3 |
| ADD3 | 0 | 74.4 | 2 |
| TMEM160 | 0 | 19.6 | 7 |
| DOCK4 | 0 | 229.9 | 1 |
| FOXRED1 | 0 | 38.9 | 2 |
| | 0 | 55.6 | 2 |
| | 0 | 31.3 | 3 |
| MRPL50 | 0 | 18.3 | 7 |
| SPR | 0 | 28 | 4 |
| ABCB1 | 0 | 7.7 | 13 |
| SEC23B | 0 | 86.4 | 2 |
| B3GNT1 | 0 | 47.1 | 2 |
| PDK3 | 0 | 46.9 | 2 |
| LETM1 | 0 | 83.3 | 2 |
| NUBPL | 0 | 26.7 | 3 |
| CUL5 | 0 | 90.9 | 1 |
| LRP1B | 0 | 515.2 | 0 |
| YIPF4 | 0 | 27.1 | 4 |
| TSPO | 0 | 18.5 | 5 |
| NSUN4 | 0 | 37.3 | 3 |
| POLR1B | 0 | 38.5 | 2 |
| | 0 | 103.1 | 1 |
| VBP1 | 0 | 22.6 | 4 |
| KTN1 | 0 | 156.2 | 1 |
| CNOT8 | 0 | 14.6 | 9 |

| | | | |
|---|---|---|---|
| HERC3 | 0 | 56.5 | 1 |
| FAM105B | 0 | 40.2 | 2 |
| CCR6 | 0 | 42.5 | 2 |

**Supplementary Table 4. Proteins only captured by circKIF18A probe.**

| Gene symbol | Protein_score |
|---|---|
| MCM7 | 150.66 |
| PRKDC | 76.98 |
| ACTA2 | 67.61 |
| ACTN4 | 66.95 |
| TPM1 | 66.65 |
| MYO5A | 56.55 |
| HNRNPM | 54.44 |
| ACTBL2 | 51.6 |
| MYO18A | 51.42 |
| COL6A3 | 50.36 |
| HSPA8 | 47.24 |
| TPM4 | 46.43 |
| ACTG1 | 44.49 |
| PCCB | 42.79 |
| ACTN1 | 41.02 |
| STAT1 | 39.59 |
| SVIL | 38.36 |
| TUBB | 37.9 |
| LIMA1 | 36.87 |
| FLII | 36.41 |
| ATP2A2 | 35.69 |
| TPM2 | 35.22 |
| TUBB3 | 34.92 |
| MYO1B | 33.27 |
| MYH14 | 32.72 |
| MYO6 | 32.56 |
| DDX21 | 31.82 |
| ANXA2 | 31.32 |
| GSN | 30.18 |
| POTEF | 29.43 |
| NCL | 26.93 |
| SMARCA5 | 26.74 |
| HNRNPUL1 | 26.56 |
| MYO5B | 26.14 |
| PABPC1 | 24.76 |
| HNRNPU | 24.76 |
| KIF5B | 24.64 |
| LCP1 | 23.98 |
| SRRM2 | 23.78 |
| ERLIN2 | 23.31 |
| SMCHD1 | 23.07 |
| HSPA9 | 22.49 |
| NOP56 | 22.36 |
| TUBA4A | 21.79 |
| GART | 21.74 |
| RPL15 | 21.66 |

| | |
|---|---|
| VARS | 21.6 |
| MYO1D | 20.94 |
| MYO1E | 20.89 |
| MSH6 | 20.54 |
| IMMT | 20.46 |
| ATAD3A | 20.36 |
| LMNB1 | 20.28 |
| TMOD3 | 20.13 |
| TOP2B | 20.11 |
| MYBBP1A | 20.03 |
| RSL1D1 | 19.79 |
| MYL6B | 19.71 |
| XRCC6 | 19.56 |
| SUPT16H | 19.55 |
| LRRFIP2 | 19.36 |
| EIF4A3 | 18.81 |
| SAMHD1 | 18.67 |
| SFPQ | 18.64 |
| RPS3A | 18.27 |
| MCM6 | 17.87 |
| TMOD1 | 17.86 |
| CDC42BPB | 17.8 |
| SART3 | 17.67 |
| TOP2A | 17.66 |
| AP3D1 | 17.64 |
| ABCD3 | 17.58 |
| CCT5 | 17.21 |
| GNB2L1 | 17.09 |
| PFKL | 17.04 |
| CAPN1 | 16.93 |
| MAP4K4 | 16.83 |
| PALLD | 16.68 |
| CAP1 | 16.45 |
| RAD50 | 16.39 |
| PARP1 | 16.19 |
| RPS27A | 16.12 |
| HNRNPR | 15.85 |
| DARS | 15.49 |
| ESYT1 | 15.42 |
| AFG3L2 | 15.33 |
| CCT8 | 15.32 |
| NAT10 | 15.13 |
| PFKP | 15 |
| PHGDH | 14.92 |
| KRT75 | 14.92 |
| NDUFS1 | 14.73 |
| LRPPRC | 14.56 |
| CYFIP1 | 14.55 |

| | |
|---|---|
| HNRNPA0 | 14.51 |
| VCL | 14.5 |
| RPL13AP25 | 14.45 |
| CEBPZ | 14.34 |
| EIF3E | 14.07 |
| RPL23A | 14.06 |
| MCM2 | 14 |
| UBR4 | 13.93 |
| DTX3L | 13.89 |
| ADAR | 13.77 |
| SLC25A13 | 13.76 |
| CACYBP | 13.75 |
| HADHA | 13.68 |
| HNRNPH1 | 13.61 |
| ENO1 | 13.61 |
| GTF2I | 13.57 |
| GAPDH | 13.39 |
| ATP13A1 | 13.32 |
| DDX50 | 13.21 |
| PKM | 13.15 |
| PDCD11 | 13.14 |
| FHL2 | 13 |
| VDAC1 | 12.54 |
| PML | 12.45 |
| RPL10A | 12.39 |
| TRIM28 | 12.33 |
| RNF213 | 12.24 |
| TAP2 | 12.16 |
| LMNB2 | 12.03 |
| HP1BP3 | 12 |
| CAPZA2 | 11.95 |
| DDX23 | 11.93 |
| DNAJA1 | 11.88 |
| SGPL1 | 11.84 |
| MRPS5 | 11.8 |
| CSDE1 | 11.78 |
| MARS | 11.71 |
| ASPH | 11.7 |
| STT3A | 11.69 |
| COL5A1 | 11.47 |
| PLS3 | 11.24 |
| H1F0 | 11.22 |
| DDX27 | 11.22 |
| MSH2 | 11.12 |
| AARS | 11.12 |
| LUC7L2 | 11.09 |
| XRCC5 | 11.01 |
| KRT6B | 10.98 |

| | |
|---|---|
| DHX30 | 10.83 |
| TOP1 | 10.81 |
| DNAJC13 | 10.73 |
| DPYSL3 | 10.67 |
| EHD4 | 10.67 |
| AP3B1 | 10.64 |
| RANBP2 | 10.61 |
| GFPT1 | 10.57 |
| UGGT1 | 10.55 |
| PLAA | 10.3 |
| LGALS3BP | 10.18 |
| HIST1H1C | 10.09 |
| IGF2BP2 | 10.04 |
| YWHAQ | 9.95 |
| LRRFIP1 | 9.95 |
| PDHA1 | 9.78 |
| ACTR3 | 9.77 |
| WARS | 9.76 |
| MYL1 | 9.67 |
| ASCC3 | 9.62 |
| DKC1 | 9.62 |
| UBA1 | 9.52 |
| IQGAP2 | 9.46 |
| DDX18 | 9.42 |
| AP2B1 | 9.23 |
| PSMD6 | 9.15 |
| ACSL3 | 9.14 |
| LMO7 | 9.13 |
| SMC4 | 9.11 |
| SPTLC1 | 9.1 |
| SMC2 | 9.06 |
| PDLIM4 | 8.96 |
| RPL7 | 8.96 |
| LBR | 8.92 |
| WDR11 | 8.88 |
| PPP1CB | 8.84 |
| KHDRBS1 | 8.81 |
| UQCRC1 | 8.75 |
| RPL35 | 8.71 |
| USO1 | 8.66 |
| VDAC2 | 8.63 |
| RPL27A | 8.58 |
| HYOU1 | 8.57 |
| SLC25A5 | 8.55 |
| OAT | 8.54 |
| PRPF4B | 8.53 |
| OAS3 | 8.51 |
| MYLK | 8.5 |

| | |
|---|---|
| HNRNPH2 | 8.4 |
| RBBP4 | 8.4 |
| KPNA6 | 8.35 |
| RCC2 | 8.35 |
| PYGB | 8.34 |
| ITPR3 | 8.22 |
| SRPK2 | 8.2 |
| UHRF1 | 8.19 |
| NUP210 | 8.19 |
| GTPBP4 | 8.18 |
| CGGBP1 | 8.13 |
| EIF2S2 | 8.12 |
| SFXN3 | 8.08 |
| RPL36AL | 7.95 |
| STAT2 | 7.95 |
| AHSA1 | 7.89 |
| BOP1 | 7.89 |
| ERAP1 | 7.85 |
| QARS | 7.83 |
| CORO2B | 7.82 |
| HSD17B4 | 7.72 |
| DIS3 | 7.72 |
| MRPS15 | 7.66 |
| EBNA1BP2 | 7.66 |
| CDK9 | 7.66 |
| TECR | 7.65 |
| HNRNPDL | 7.64 |
| SUGP2 | 7.59 |
| NDUFA10 | 7.59 |
| CALD1 | 7.57 |
| PGAM5 | 7.55 |
| ALDH2 | 7.55 |
| ACLY | 7.54 |
| KRT8 | 7.52 |
| SCAF11 | 7.5 |
| NCLN | 7.45 |
| PARP9 | 7.41 |
| SPATS2L | 7.32 |
| ALDH3A2 | 7.29 |
| RAB18 | 7.25 |
| DCTN4 | 7.25 |
| RPL28 | 7.24 |
| KIFC1 | 7.23 |
| TBL1XR1 | 7.19 |
| EIF3L | 7.16 |
| RAB1A | 7.16 |
| HECTD1 | 7.15 |
| DHRS2 | 7.13 |

| | |
|---|---|
| MAP7D1 | 7.12 |
| UGDH | 7.09 |
| IFI16 | 7.09 |
| CAAP1 | 7.07 |
| CDK1 | 7.07 |
| NOP2 | 7.01 |
| FMNL3 | 7.01 |
| CHD5 | 6.99 |
| TBL3 | 6.95 |
| RPS23 | 6.94 |
| SFXN1 | 6.91 |
| STAU1 | 6.89 |
| SSRP1 | 6.88 |
| DLST | 6.85 |
| MBNL1 | 6.84 |
| NDUFA13 | 6.84 |
| HELZ2 | 6.83 |
| U2AF1 | 6.83 |
| RPL26 | 6.81 |
| PSMC5 | 6.8 |
| BCLAF1 | 6.78 |
| NUP133 | 6.76 |
| EMC1 | 6.75 |
| DDX6 | 6.72 |
| RECQL | 6.69 |
| PLOD1 | 6.66 |
| EML4 | 6.62 |
| PCK2 | 6.6 |
| NLRP2 | 6.6 |
| NACA | 6.58 |
| PPP2R2A | 6.55 |
| UFL1 | 6.52 |
| HNRNPH3 | 6.5 |
| EIF6 | 6.5 |
| SLC25A12 | 6.5 |
| CALM2 | 6.5 |
| ANKFY1 | 6.48 |
| PPP6R3 | 6.45 |
| PTGES3 | 6.43 |
| RPS4X | 6.43 |
| EIF2AK2 | 6.41 |
| UTP20 | 6.36 |
| DDX39B | 6.34 |
| SRSF5 | 6.33 |
| XPO1 | 6.33 |
| SNRPD3 | 6.27 |
| HSPH1 | 6.27 |
| ETFB | 6.25 |

| | |
|---|---|
| CFL1 | 6.24 |
| RPS16 | 6.2 |
| EEF1G | 6.17 |
| DDX24 | 6.16 |
| BAZ1B | 6.15 |
| PPP3CA | 6.15 |
| HCFC1 | 6.15 |
| NUP88 | 6.12 |
| NUP98 | 6.11 |
| SNTB2 | 6.1 |
| SLC25A24 | 6.1 |
| SF3B3 | 6.05 |
| PFN1 | 6.03 |
| DHCR24 | 6.02 |
| AKAP17A | 6 |
| TSG101 | 5.98 |
| PSMC3 | 5.98 |
| GNL3 | 5.96 |
| LRRC40 | 5.96 |
| ACSL4 | 5.94 |
| SRSF6 | 5.93 |
| STRN3 | 5.93 |
| GSPT1 | 5.92 |
| VCP | 5.92 |
| SKIV2L2 | 5.91 |
| RPLP2 | 5.91 |
| AP1G1 | 5.9 |
| PDLIM7 | 5.9 |
| MAP1B | 5.89 |
| YARS | 5.88 |
| STT3B | 5.86 |
| NARS | 5.85 |
| ZCCHC8 | 5.85 |
| OPA1 | 5.83 |
| ATP2C1 | 5.82 |
| RPL36A | 5.82 |
| GNB1 | 5.79 |
| DSTN | 5.78 |
| DDX54 | 5.78 |
| PMPCB | 5.76 |
| CCT7 | 5.74 |
| RAB3GAP2 | 5.74 |
| CTPS1 | 5.73 |
| NASP | 5.72 |
| SRRT | 5.72 |
| HLA-B | 5.7 |
| CLPX | 5.68 |
| RXRB | 5.67 |

| | |
|---|---|
| AHCYL1 | 5.66 |
| DNM2 | 5.64 |
| RBMX | 5.63 |
| SLFN5 | 5.63 |
| MTA2 | 5.62 |
| ABCF3 | 5.61 |
| DNAJA2 | 5.58 |
| RPS27 | 5.55 |
| AGPS | 5.49 |
| MRPS27 | 5.49 |
| ACOT7 | 5.47 |
| PFN2 | 5.46 |
| APOBEC3C | 5.39 |
| NUP160 | 5.39 |
| MAT2A | 5.36 |
| WDR18 | 5.31 |
| MRPL46 | 5.31 |
| HBS1L | 5.3 |
| ZNF638 | 5.29 |
| ITGB1 | 5.28 |
| ACTR2 | 5.27 |
| HNRNPAB | 5.26 |
| HLA-DRA | 5.23 |
| RFC2 | 5.22 |
| TUBG1 | 5.21 |
| MRPS22 | 5.2 |
| TUSC3 | 5.2 |
| FAF2 | 5.17 |
| SREK1 | 5.16 |
| CPT1A | 5.13 |
| TFRC | 5.11 |
| DUSP23 | 5.1 |
| RPS19 | 5.02 |
| AQR | 4.99 |
| RING1 | 4.98 |
| RNH1 | 4.93 |
| CAPZA1 | 4.93 |
| LONP1 | 4.88 |
| PTPLAD1 | 4.87 |
| EHD1 | 4.86 |
| AP3S1 | 4.86 |
| DNMT1 | 4.85 |
| EMC2 | 4.82 |
| RAB2A | 4.8 |
| PREB | 4.79 |
| RB1 | 4.73 |
| SUCLG1 | 4.71 |
| CCAR1 | 4.71 |

| | |
|---|---|
| SRRM1 | 4.68 |
| UBAP2L | 4.64 |
| PCBP1 | 4.62 |
| NDUFV3 | 4.61 |
| SEC23IP | 4.6 |
| CANX | 4.58 |
| CMAS | 4.58 |
| NTPCR | 4.57 |
| ANKRD52 | 4.57 |
| RAB5C | 4.56 |
| MTCH2 | 4.55 |
| CALU | 4.55 |
| HLA-C | 4.54 |
| SSB | 4.53 |
| ATP6V1A | 4.53 |
| HLA-A | 4.52 |
| PSMD14 | 4.51 |
| RPL36 | 4.51 |
| SLC16A3 | 4.49 |
| ILKAP | 4.47 |
| BCAP31 | 4.47 |
| PSMB5 | 4.44 |
| SRP14 | 4.42 |
| SRSF9 | 4.41 |
| TIMM44 | 4.41 |
| SLC30A7 | 4.39 |
| CSE1L | 4.37 |
| UTP18 | 4.35 |
| FKBP5 | 4.34 |
| ACADM | 4.34 |
| ZFP91 | 4.34 |
| CKAP5 | 4.34 |
| CRNKL1 | 4.33 |
| USP9X | 4.33 |
| IPO7 | 4.32 |
| VAPA | 4.31 |
| BLOC1S5 | 4.28 |
| PWP1 | 4.27 |
| CHD3 | 4.26 |
| TRMT112 | 4.21 |
| MAGEC2 | 4.2 |
| FLOT2 | 4.2 |
| NDUFV1 | 4.19 |
| SAFB2 | 4.19 |
| DDX19B | 4.17 |
| PCID2 | 4.16 |
| ATP5F1 | 4.16 |
| CNOT1 | 4.14 |

| | |
|---|---|
| EIF3H | 4.13 |
| KDM5D | 4.13 |
| MFN2 | 4.12 |
| DLD | 4.12 |
| GNAI2 | 4.11 |
| TMEM165 | 4.11 |
| CSNK2B | 4.1 |
| HNRNPD | 4.1 |
| MDH2 | 4.1 |
| NCBP2 | 4.1 |
| MRE11A | 4.09 |
| SH3BGRL2 | 4.09 |
| DHX37 | 4.09 |
| STRN4 | 4.09 |
| CCDC47 | 4.07 |
| PYCR2 | 4.07 |
| TARDBP | 4.06 |
| MTIF2 | 4.06 |
| DECR1 | 4.05 |
| EXOC4 | 4.05 |
| APOL2 | 4.05 |
| YWHAE | 4.05 |
| SHMT2 | 4.04 |
| EEF1D | 4.04 |
| IFIT3 | 4.04 |
| PSMD13 | 4.04 |
| RPL14 | 4.03 |
| KRT18 | 4.03 |
| KIF2C | 4.02 |
| TM9SF3 | 4.02 |
| RPL18A | 4 |
| KCMF1 | 3.98 |
| SLC6A19 | 3.98 |
| DNAJC16 | 3.98 |
| THOC2 | 3.97 |
| RFC4 | 3.97 |
| AP2M1 | 3.97 |
| TBC1D5 | 3.97 |
| ITM2B | 3.97 |
| RANBP1 | 3.97 |
| MRPS23 | 3.96 |
| PAPOLA | 3.96 |
| ECH1 | 3.95 |
| DDX56 | 3.95 |
| NFKB2 | 3.94 |
| TWF1 | 3.93 |
| ACTR1A | 3.92 |
| PTPN1 | 3.92 |

| | |
|---|---|
| ZMPSTE24 | 3.92 |
| FKBP4 | 3.91 |
| TRAPPC11 | 3.91 |
| CAP2 | 3.9 |
| MAGEB2 | 3.9 |
| ACAA2 | 3.9 |
| PWP2 | 3.9 |
| TCOF1 | 3.89 |
| AP1B1 | 3.88 |
| ATP6V1H | 3.88 |
| WDR3 | 3.87 |
| GBP1 | 3.87 |
| SUCLA2 | 3.85 |
| GTF3C1 | 3.85 |
| FAM208A | 3.84 |
| FXR2 | 3.84 |
| STK4 | 3.84 |
| NOL10 | 3.83 |
| SRP9 | 3.82 |
| MT1X | 3.8 |
| ATP2B4 | 3.8 |
| TRIP4 | 3.8 |
| PAFAH1B3 | 3.79 |
| UBE3C | 3.79 |
| EXOSC10 | 3.78 |
| ITPA | 3.78 |
| STAU2 | 3.78 |
| FHOD1 | 3.78 |
| TCEB1 | 3.77 |
| SUPT4H1 | 3.76 |
| SSBP1 | 3.76 |
| TKT | 3.75 |
| FAM129B | 3.75 |
| MAGED1 | 3.75 |
| SEC61A1 | 3.75 |
| DNAJB11 | 3.74 |
| COMMD2 | 3.74 |
| H2AFY | 3.73 |
| ASCC1 | 3.72 |
| FKBP10 | 3.72 |
| RRP9 | 3.71 |
| MAP1S | 3.71 |
| PDIA5 | 3.7 |
| SAMD9L | 3.69 |
| CAPNS1 | 3.68 |
| OGT | 3.68 |
| AKAP8L | 3.68 |
| FAF1 | 3.67 |

| | |
|---|---|
| GNA13 | 3.66 |
| NRP1 | 3.64 |
| DHRS4 | 3.64 |
| IDH1 | 3.64 |
| SPATA5L1 | 3.64 |
| TMOD2 | 3.63 |
| PPFIBP1 | 3.61 |
| RTN4 | 3.61 |
| CDK5 | 3.61 |
| TROVE2 | 3.6 |
| NPLOC4 | 3.56 |
| VPS18 | 3.55 |
| WDR6 | 3.55 |
| TEX11 | 3.54 |
| FARSB | 3.54 |
| H1FX | 3.54 |
| BMP1 | 3.52 |
| PRDX1 | 3.52 |
| PBXIP1 | 3.5 |
| RALY | 3.5 |
| VWA8 | 3.49 |
| PSMC1 | 3.48 |
| TIMM21 | 3.48 |
| IDH3B | 3.47 |
| AKAP2 | 3.45 |
| SF3A3 | 3.45 |
| PARP14 | 3.45 |
| DNAJC9 | 3.44 |
| EIF3F | 3.43 |
| GYS1 | 3.39 |
| NCAPG | 3.38 |
| MRPS2 | 3.38 |
| MBOAT7 | 3.36 |
| ASH2L | 3.35 |
| NOL6 | 3.33 |
| TRRAP | 3.33 |
| EIF2A | 3.3 |
| RRS1 | 3.3 |
| MVP | 3.28 |
| RHOT1 | 3.25 |
| RRP1B | 3.24 |
| DDX10 | 3.16 |
| LEPREL4 | 3.11 |
| OSBPL3 | 3.04 |
| PELO | 3.01 |
| ARPC2 | 3 |
| PRPF38A | 2.95 |
| RAB14 | 2.9 |

| | |
|---|---|
| MRPL48 | 2.89 |
| DAPK3 | 2.87 |
| EMD | 2.85 |
| SPG20 | 2.84 |
| PRRC2C | 2.84 |
| NMNAT1 | 2.83 |
| SMARCB1 | 2.82 |
| AKR1A1 | 2.81 |
| PRKCA | 2.78 |
| NOC2L | 2.76 |
| RNF114 | 2.75 |
| MRPL55 | 2.73 |
| CDC42BPA | 2.71 |
| PDIA4 | 2.71 |
| VPS39 | 2.68 |
| FAHD2A | 2.67 |
| BLVRB | 2.65 |
| ITGA11 | 2.64 |
| TRIM3 | 2.63 |
| ARHGAP1 | 2.58 |
| TRIM25 | 2.58 |
| LYAR | 2.58 |
| LEMD2 | 2.56 |
| CALR | 2.54 |
| ABCB7 | 2.53 |
| ANXA11 | 2.51 |
| SUN1 | 2.5 |
| KIF1B | 2.5 |
| NDUFS2 | 2.48 |
| SSR4 | 2.48 |
| NUP107 | 2.47 |
| STARD9 | 2.47 |
| PSMD10 | 2.47 |
| PSMD12 | 2.47 |
| TWISTNB | 2.45 |
| F2 | 2.45 |
| NEMF | 2.44 |
| AFAP1 | 2.44 |
| NR1H2 | 2.44 |
| PRKD2 | 2.41 |
| HBA2; HBA1 | 2.41 |
| C2orf18 | 2.41 |
| RAP2C | 2.4 |
| ACOT8 | 2.4 |
| METAP1 | 2.4 |
| ACP1 | 2.39 |
| WDR43 | 2.39 |
| H2AFY2 | 2.39 |

| | |
|---|---|
| PRPF19 | 2.39 |
| MAGEA1 | 2.38 |
| ETHE1 | 2.38 |
| GET4 | 2.37 |
| MLLT11 | 2.36 |
| RBM8A | 2.36 |
| RPS28 | 2.35 |
| UBTF | 2.34 |
| PYCRL | 2.34 |
| XPO7 | 2.34 |
| TMCO1 | 2.34 |
| RDX | 2.34 |
| HSPA4 | 2.34 |
| MOV10 | 2.32 |
| NCOR2 | 2.31 |
| PIGK | 2.31 |
| TRA2A | 2.3 |
| TRAFD1 | 2.3 |
| CPSF4 | 2.3 |
| PRPS2 | 2.29 |
| RRP12 | 2.29 |
| RPS15A | 2.29 |
| NPEPPS | 2.29 |
| CLNS1A | 2.29 |
| TMED10 | 2.29 |
| PHF14 | 2.29 |
| OGDH | 2.29 |
| RALYL | 2.29 |
| KRT86 | 2.29 |
| EDARADD | 2.28 |
| DYNLL2 | 2.28 |
| TM9SF2 | 2.28 |
| NKRF | 2.27 |
| SH3BP4 | 2.27 |
| LOC100132015 | 2.27 |
| EWSR1 | 2.26 |
| STRN | 2.26 |
| HDAC2 | 2.26 |
| NDUFB4 | 2.26 |
| PAF1 | 2.26 |
| RANGAP1 | 2.26 |
| FDXR | 2.25 |
| NUSAP1 | 2.25 |
| NEXN | 2.24 |
| PPIA | 2.24 |
| HAT1 | 2.24 |
| ZNF598 | 2.24 |
| NUP85 | 2.23 |

| | |
|---|---|
| CPD | 2.23 |
| NTMT1 | 2.23 |
| NUP188 | 2.22 |
| MRPL24 | 2.22 |
| UBR5 | 2.21 |
| METTL3 | 2.21 |
| SETDB1 | 2.21 |
| NIPSNAP1 | 2.21 |
| RAB3GAP1 | 2.21 |
| RAB7L1 | 2.2 |
| CAMK2D | 2.2 |
| HM13 | 2.19 |
| NOL9 | 2.19 |
| ARAF | 2.19 |
| ZNF207 | 2.19 |
| CLTA | 2.19 |
| SUB1 | 2.18 |
| PTK2 | 2.18 |
| TRA2B | 2.18 |
| LPCAT1 | 2.18 |
| SPATA5 | 2.17 |
| EIF2B4 | 2.17 |
| KRAS | 2.16 |
| TXN | 2.16 |
| L3HYPDH | 2.16 |
| FAU | 2.16 |
| LIN7C | 2.15 |
| SRM | 2.15 |
| RFC3 | 2.15 |
| ACTL6A | 2.15 |
| RAB27A | 2.14 |
| PSMA1 | 2.14 |
| DHX33 | 2.14 |
| GEMIN2 | 2.14 |
| TMX2 | 2.14 |
| SNRPB2 | 2.14 |
| HLA-DRB1 | 2.13 |
| DOCK10 | 2.13 |
| ARHGDIA | 2.13 |
| IK | 2.13 |
| KIAA0196 | 2.12 |
| KIF21A | 2.12 |
| PDLIM5 | 2.12 |
| GMPPB | 2.12 |
| MTCH1 | 2.11 |
| EXT2 | 2.11 |
| HMHA1 | 2.11 |
| MKI67 | 2.11 |

| | |
|---|---|
| MRPL43 | 2.11 |
| SLC16A1 | 2.11 |
| PDXDC1 | 2.11 |
| CTSA | 2.11 |
| SURF4 | 2.11 |
| SNW1 | 2.11 |
| PDS5A | 2.11 |
| HSDL2 | 2.11 |
| CLCN7 | 2.11 |
| ACSL1 | 2.11 |
| PDE5A | 2.11 |
| MAN1A2 | 2.1 |
| KRR1 | 2.1 |
| PNKP | 2.1 |
| SERPINE2 | 2.1 |
| ZAK | 2.1 |
| BAG6 | 2.1 |
| AGPAT6 | 2.09 |
| PURA | 2.09 |
| SKIV2L | 2.09 |
| TTLL12 | 2.09 |
| SRPK1 | 2.09 |
| DNAJB6 | 2.08 |
| FTH1 | 2.08 |
| RPL24 | 2.08 |
| TGFB1I1 | 2.08 |
| GMDS | 2.08 |
| SAR1A | 2.07 |
| GNAI3 | 2.07 |
| WDR46 | 2.07 |
| GAPVD1 | 2.07 |
| RHOG | 2.07 |
| NDUFAF3 | 2.06 |
| NEDD1 | 2.06 |
| ALKBH5 | 2.05 |
| NSA2 | 2.05 |
| CCAR2 | 2.05 |
| ILVBL | 2.05 |
| NUP214 | 2.04 |
| CFI | 2.04 |
| UBR1 | 2.04 |
| SMU1 | 2.04 |
| NDUFS8 | 2.04 |
| PTPLB | 2.04 |
| TRAF2 | 2.04 |
| XRN2 | 2.04 |
| PTCD3 | 2.04 |
| HIST3H3 | 2.03 |

| | |
|---|---|
| HLA-DPB1 | 2.03 |
| HIST2H3PS2 | 2.03 |
| SPANXD | 2.03 |
| USMG5 | 2.02 |
| NGDN | 2.02 |
| YY1 | 2.02 |
| FTSJ3 | 2.02 |
| NUP54 | 2.02 |
| LEPREL1 | 2.02 |
| CDC37 | 2.01 |
| GNE | 2.01 |
| PYCR1 | 2.01 |
| EIF4H | 2 |
| NBAS | 2 |
| POLR2E | 2 |
| EEA1 | 2 |
| EXOSC9 | 2 |
| PTPN23 | 2 |
| HTRA1 | 1.99 |
| NAV1 | 1.99 |
| CDKAL1 | 1.98 |
| TTC37 | 1.98 |
| CHTOP | 1.98 |
| C3orf17 | 1.98 |
| KANK2 | 1.98 |
| ARL2 | 1.98 |
| PPP1R12A | 1.98 |
| NCBP1 | 1.98 |
| RPL29 | 1.98 |
| PTBP1 | 1.98 |
| TOMM22 | 1.98 |
| RPS26 | 1.97 |
| NUP155 | 1.97 |
| TES | 1.97 |
| UTP6 | 1.97 |
| HSD17B12 | 1.97 |
| METTL7B | 1.97 |
| TRIP12 | 1.97 |
| HOOK3 | 1.96 |
| WDR33 | 1.96 |
| ANGPTL2 | 1.96 |
| CHD8 | 1.96 |
| ACOT9 | 1.96 |
| EPHX1 | 1.96 |
| CPNE3 | 1.95 |
| LOXL2 | 1.95 |
| EMILIN1 | 1.95 |
| RFC1 | 1.95 |

| | |
|---|---|
| SLC35B2 | 1.95 |
| DNAJC25 | 1.95 |
| PSPC1 | 1.94 |
| LPGAT1 | 1.94 |
| MIB1 | 1.94 |
| RPA3 | 1.94 |
| CC2D1A | 1.94 |
| KIF16B | 1.94 |
| RABL3 | 1.93 |
| IFIT1 | 1.93 |
| CYB5R1 | 1.93 |
| PLCD3 | 1.93 |
| CIAO1 | 1.92 |
| CCZ1B | 1.92 |
| CYP51A1 | 1.92 |
| DNAJA3 | 1.92 |
| AHCTF1 | 1.92 |
| DDX20 | 1.92 |
| GLYR1 | 1.92 |
| NUDCD3 | 1.91 |
| RELA | 1.91 |
| TIAL1 | 1.91 |
| HTATSF1 | 1.91 |
| VAV2 | 1.91 |
| SURF6 | 1.91 |
| PHRF1 | 1.91 |
| SULF2 | 1.91 |
| SCO2 | 1.9 |
| PRPF38B | 1.9 |
| NCAPD2 | 1.9 |
| KIAA0020 | 1.9 |
| SUCLG2 | 1.9 |
| PPP6C | 1.9 |
| GIT2 | 1.89 |
| CSTF1 | 1.89 |
| SLC25A11 | 1.89 |
| ATP5O | 1.89 |
| CORO1B | 1.88 |
| OSBPL8 | 1.88 |
| ATP6V0D1 | 1.88 |
| MLLT4 | 1.88 |
| PPP2CA | 1.88 |
| TMEM2 | 1.88 |
| PGAM1 | 1.88 |
| EZH2 | 1.88 |
| FSCN1 | 1.88 |
| NDUFA5 | 1.88 |
| LMAN2 | 1.88 |

| | |
|---|---|
| TRIM56 | 1.87 |
| FNDC3A | 1.87 |
| MMS19 | 1.87 |
| PXN | 1.87 |
| AVEN | 1.87 |
| CLPTM1 | 1.87 |
| ZNF579 | 1.87 |
| RHOT2 | 1.86 |
| CHAF1B | 1.86 |
| NOL11 | 1.86 |
| RARS2 | 1.86 |
| GEMIN5 | 1.86 |
| CBR1 | 1.86 |
| CENPB | 1.85 |
| COX11 | 1.85 |
| PHAX | 1.85 |
| SNRPG | 1.85 |
| KPNA2 | 1.85 |
| CD2BP2 | 1.85 |
| APMAP | 1.85 |
| NHP2 | 1.85 |
| CUL2 | 1.85 |
| DHCR7 | 1.85 |
| SNRPA1 | 1.85 |
| ESF1 | 1.85 |
| AMPD2 | 1.85 |
| VPS13C | 1.84 |
| NCSTN | 1.84 |
| SETD1A | 1.84 |
| TP53BP1 | 1.84 |
| DYNLRB1 | 1.84 |
| SNX7 | 1.84 |
| DDX31 | 1.84 |
| DCAKD | 1.83 |
| PMVK | 1.83 |
| SNX3 | 1.83 |
| PRDX4 | 1.83 |
| DOCK7 | 1.83 |
| MPDZ | 1.82 |
| FAM133B | 1.82 |
| ELOVL1 | 1.82 |
| PSMB9 | 1.82 |
| ANAPC7 | 1.82 |
| SF3A1 | 1.82 |
| SLC33A1 | 1.82 |
| CORO7 | 1.82 |
| ARHGAP17 | 1.82 |
| WDR12 | 1.81 |

| | |
|---|---|
| APBA2 | 1.81 |
| CWC25 | 1.81 |
| SCAMP3 | 1.81 |
| STRBP | 1.81 |
| HLCS | 1.8 |
| ATRX | 1.8 |
| HLTF | 1.8 |
| PPP2R5C | 1.8 |
| DHX57 | 1.8 |
| PHF10 | 1.8 |
| RBM22 | 1.8 |
| PIK3C2A | 1.79 |
| POLR3A | 1.79 |
| PRPF4 | 1.79 |
| PSMD8 | 1.79 |
| RCN2 | 1.79 |
| ATP2B3 | 1.79 |
| ABCC1 | 1.79 |
| NEK9 | 1.78 |
| PRKAA1 | 1.78 |
| FBN1 | 1.78 |
| PI4KA | 1.78 |
| GPSM1 | 1.78 |
| HS2ST1 | 1.78 |
| CCRN4L | 1.78 |
| RPA1 | 1.78 |
| NAA35 | 1.78 |
| BLVRA | 1.78 |
| AKAP8 | 1.78 |
| RPL21P19 | 1.77 |
| ATP6V1B2 | 1.77 |
| AUP1 | 1.77 |
| ADSL | 1.77 |
| LIG3 | 1.77 |
| WDHD1 | 1.77 |
| NDUFA2 | 1.77 |
| TFB2M | 1.76 |
| POLR1C | 1.76 |
| AP3M1 | 1.76 |
| PAK1IP1 | 1.75 |
| DRG2 | 1.75 |
| HLA-E | 1.75 |
| C1QBP | 1.75 |
| DDX47 | 1.75 |
| MED20 | 1.74 |
| TARS2 | 1.74 |
| SACS | 1.74 |
| STOML2 | 1.74 |

| | |
|---|---|
| UTP14A | 1.74 |
| HSPA14 | 1.74 |
| ELMO2 | 1.74 |
| TBCC | 1.74 |
| NOP14 | 1.73 |
| SRC | 1.73 |
| TRIM22 | 1.73 |
| C21orf33 | 1.73 |
| PBRM1 | 1.73 |
| NNMT | 1.73 |
| TOLLIP | 1.73 |
| SNRNP27 | 1.73 |
| COG1 | 1.73 |
| TRMT10C | 1.73 |
| SNX9 | 1.73 |
| GLTSCR2 | 1.73 |
| PHLDA1 | 1.73 |
| ARPC3 | 1.72 |
| ICMT | 1.72 |
| MTOR | 1.72 |
| C14orf166 | 1.72 |
| PSMB1 | 1.72 |
| TRMT6 | 1.72 |
| TRMT1L | 1.72 |
| SGPP1 | 1.72 |
| NEU1 | 1.71 |
| NCKAP1 | 1.71 |
| TMEM11 | 1.71 |
| PIAS1 | 1.7 |
| LRCH1 | 1.7 |
| SP1 | 1.7 |
| DNAJB12 | 1.7 |
| WDR75 | 1.7 |
| PLS1 | 1.7 |
| MTHFD1L | 1.7 |
| CYB5R3 | 1.7 |
| SAMM50 | 1.7 |
| XAB2 | 1.7 |
| IMPDH1 | 1.7 |
| NLRX1 | 1.69 |
| ATAD1 | 1.69 |
| TRIM26 | 1.69 |
| HMGCS1 | 1.69 |
| RAB10 | 1.69 |
| RAB8A | 1.69 |
| PSMA3 | 1.69 |
| GTF3C4 | 1.69 |
| PAK2 | 1.69 |

| | |
|---|---|
| TANC1 | 1.69 |
| C12orf23 | 1.69 |
| YME1L1 | 1.69 |
| WWP2 | 1.69 |
| TAPBP | 1.68 |
| RPS29 | 1.68 |
| PDPR | 1.68 |
| PDP1 | 1.68 |
| MRPS6 | 1.68 |
| PTPN14 | 1.68 |
| EIF1AX | 1.68 |
| EPB41L1 | 1.67 |
| TEX10 | 1.67 |
| TBC1D15 | 1.67 |
| VPS29 | 1.67 |
| RPS13 | 1.67 |
| H2AFV | 1.67 |
| TMPO | 1.67 |
| RBM10 | 1.67 |
| PEX6 | 1.67 |
| TCF25 | 1.67 |
| RBM15B | 1.67 |
| NT5DC2 | 1.67 |
| IGBP1 | 1.67 |
| PRPF40B | 1.66 |
| CDK5RAP3 | 1.66 |
| B3GALT6 | 1.66 |
| CTTN | 1.66 |
| NAA40 | 1.66 |
| AURKB | 1.66 |
| GPX8 | 1.66 |
| WRNIP1 | 1.66 |
| ZC3HAV1 | 1.65 |
| AIMP2 | 1.65 |
| PTK7 | 1.65 |
| CNP | 1.65 |
| KIAA1033 | 1.65 |
| ZNF277 | 1.65 |
| DNM1L | 1.65 |
| GTF2E2 | 1.65 |
| GYS2 | 1.65 |
| SLC2A1 | 1.65 |
| TMEM167A | 1.64 |
| DROSHA | 1.64 |
| ANXA6 | 1.64 |
| TNS3 | 1.64 |
| NKIRAS2 | 1.64 |
| TSFM | 1.64 |

| | |
|---|---|
| CNOT10 | 1.63 |
| TSGA13 | 1.63 |
| ABCB6 | 1.63 |
| SNX4 | 1.63 |
| NUP35 | 1.63 |
| PRDX6 | 1.63 |
| LSM6 | 1.63 |
| ARL6IP6 | 1.63 |
| NQO1 | 1.63 |
| PRKCI | 1.62 |
| PIP | 1.62 |
| SPCS3 | 1.62 |
| TAGLN2 | 1.62 |
| DYM | 1.62 |
| FECH | 1.62 |
| SYNJ2BP | 1.62 |
| RAPGEF6 | 1.61 |
| SH3PXD2A | 1.61 |
| IKBIP | 1.61 |
| FHL1 | 1.61 |
| ANLN | 1.61 |
| PTPRK | 1.61 |
| HEATR1 | 1.61 |
| NUMB | 1.61 |
| TOE1 | 1.6 |
| MEX3D | 1.6 |
| IMP3 | 1.6 |
| DHX38 | 1.6 |

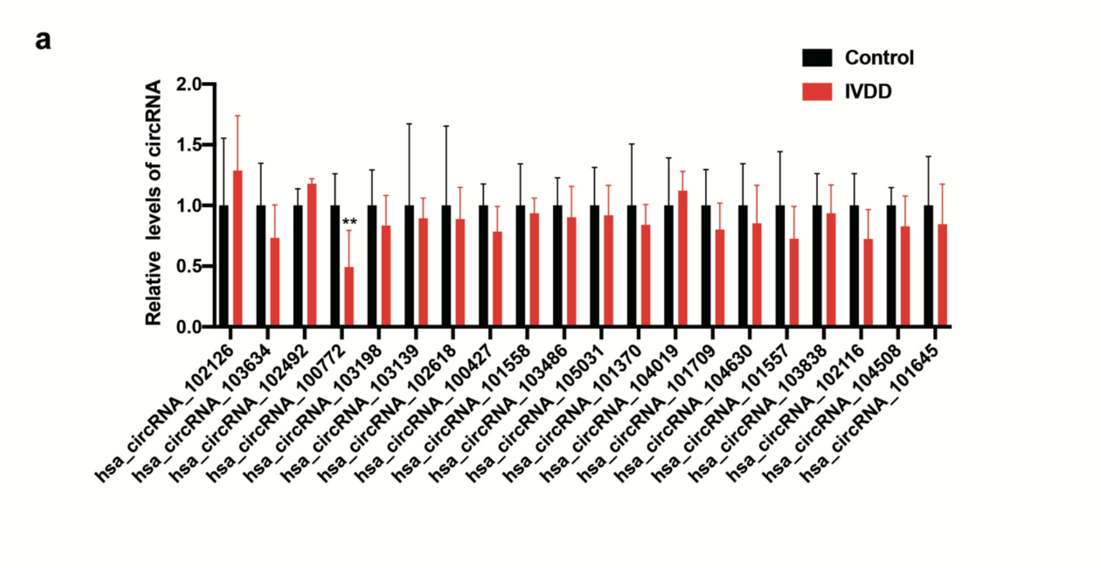


**Supplementary Fig 1. The expression of hsa_circRNA_100772 (termed as hsa_circ_0021535) shows the most remarkable reduction in IVDD.** (a) The qRT-PCR assay revealed the expression of top 20 downregulated circRNAs in IVDD (n=5, five different donors; *: versus the control group, * $P < 0.05$, ** $P < 0.01$). Data represent the mean ± SD.

a

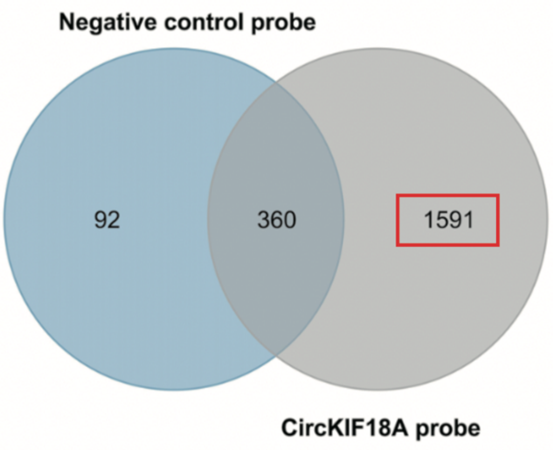


**Supplementary Fig 2. The strategy for searching the RBP binding to circKIF18A.**

(a) The Venn graph about proteins capture by the negative control probe and the circKIF18A probe. Red frame: proteins captured by the circKIF18A probe but not negative control probe.

a

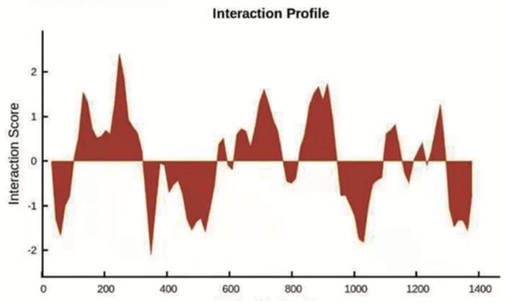


b

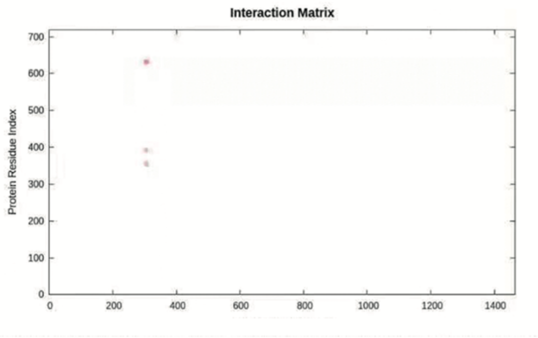


c

| | Protein region | RNA region | | Protein region | RNA region |
|---|---|---|---|---|---|
| 1 | 619-670 | 291-350 | 11 | 201-252 | 610-669 |
| 2 | 376-427 | 291-350 | 12 | 201-252 | 726-785 |
| 3 | 344-395 | 291-350 | 13 | 569-620 | 291-350 |
| 4 | 201-252 | 1248-1307 | 14 | 369-420 | 291-351 |
| 5 | 576-627 | 291-350 | 15 | 251-302 | 291-352 |
| 6 | 44-95 | 291-350 | 16 | 376-427 | 276-335 |
| 7 | 619-670 | 276-335 | 17 | 451-502 | 291-350 |
| 8 | 201-252 | 871-930 | 18 | 626-677 | 291-351 |
| 9 | 201-252 | 1146-1205 | 19 | 394-445 | 1248-1307 |
| 10 | 469-520 | 291-350 | 20 | 201-252 | 900-959 |

d

**Amino acid sequence of wild type MCM7:**

MALKDYALEKEKVKKFLQEFYQDDELGKKQFKYGNQLVRLAHREQVALYVDLDDVAEDDPELVDSICENARRY
AKLFADAVQELLPQYKEREVVNKDVLDVYIEHRLMMEQRSRDPGMVRSPQNQYPAELMRRFELYFQGPSSNKP
RVIREVRADSVGKLVTVRGIVTRVSEVKPKMVVATYTCDQCGAETYQPIQSPTFMPLIMCPSQECQTNRSGGRLY
LQTRGSRFIKFQEMKMQEHSDQVPVGNIPRSITVLVEGENTRIAQPGDHVSVTGIFLPILRTGFRQVVQGLLSETYL
EAHRIVKMNKSEDDESGAGELTREELRQIAEEDFYEKLAASIAPEIYGHEDVKKALLLLLVGGVDQSPRGMKIRGN
INICLMGDPGVAKSQLLSYIDRLAPRSQYTTGRGSSGVGLTAAVLRDSVSGELTLEGGALVLADQGVCCIDEFDKM
AEADRTAIHEVMEQQTISIAKAGILTTLNARCSILAAANPAYGRYNPRRSLEQNIQLPAALLSRFDLLWLIQDRPDRD
NDLRLAQHITYVHQHSRQPPSQFEPLDMKLMRRYIAMCREKQPMVPESLADYITAAYVEMRREAWASKDATYTSA
RTLLAILRLSTALARLRMVDVVEKEDVNEAIRLMEMSKDSLLGDKGQTARTQRPADVIFATVRELVSGGRSVRFSE
AEQRCVSRGFTPAQFQAALDEYEELNVWQVNASRTRITFV

**Amino acid sequence of mutant MCM7:**

MALKDYALEKEKVKKFLQEFYQDDELGKKQFKYGNQLVRLAHREQVALYVDLDDVAEDDPELVDSICENARRY
AKLFADAVQELLPQYKEREVVNKDVLDVYIEHRLMMEQRSRDPGMVRSPQNQYPAELMRRFELYFQGPSSNKP
RVIREVRADSVGKLVTVRGIVTRVSEVKPKMVVATYTCDQCGAETYQPIQSPTFMPLIMCPSQECQTNRSGGRLY
LQTRGSRFIKFQEMKMQEHSDQVPVGNIPRSITVLVEGENTRIAQPGDHVSVTGIFLPILRTGFRQVVQGLLSETYL
EAHRIVKMNKSEDDESGAGELTREELRQIAEEDFYEKLAASIAPEIYGHEDVKKALLLLLVGGVDQSPRGMKIRGN
INICLMGDPGVAKSQLLSYIDRLAPRSQYTTGRGSSGVGLTAAVLRDSVSGELTLEGGALVLADQGVCCIDEFDKM
AEADRTAIHEVMEQQTISIAKAGILTTLNARCSILAAANPAYGRYNPRRSLEQNIQLPAALLSRFDLLWLIQDRPDRD
NDLRLAQHITYVHQHSRQPPSQFEPLDMKLMRRYIAMCREKQPMVPESLADYITAAYVEMRREAWASKDATYTSA
RTLLAILRLSTALARLRMVDVVEKEDVNEAIRLMEMDKDDAAGDKGQTARTQRPADVIFATVRELVSGGRSVRFSE
AEQRCVSRGFTPAQFQAALDEYEELNVWQVNASRTRITFV

**Supplementary Fig 3. CircKIF18A interacts with MCM7 to prevent UBE3A-mediated degradation in NPCs.** (a) The interaction profile between circKIF18A and MCM7. (b) The interaction matrix between circKIF18A and MCM7.

(c) The segment of interaction between circKIF18A and MCM7. Red frame: the most potential binding regions of circKIF18A and MCM7. (d) The sequences of wild type MCM7 and mutant MCM7. Underline: the L2G box; Red: mutant amino acids.

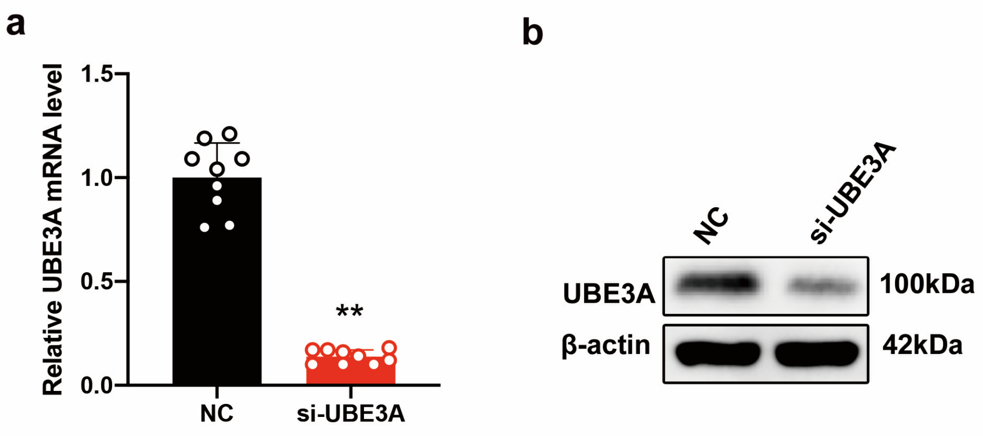


**Supplementary Fig 4. The mRNA expression and protein level in NPCs transfected with or without si-UBE3A.** (a) The mRNA level of UBE3A in NPCs transfected with or without si-UBE3A (n=3, three different donors for three individual experiments; *: versus NC, * P < 0.05, ** P < 0.01). (b) The protein level of UBE3A in NPCs transfected with or without si-UBE3A (n=3, three different donors). All data represent the mean ± SD.

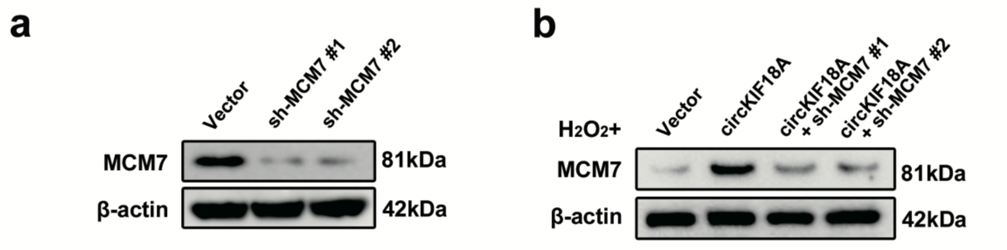


**Supplementary Fig 5. The effect of sh-MCM7 and circKIF18A overexpression in NPCs treated with or without $H_2O_2$.** (a) The protein level of MCM7 in NPCs transfected with or without sh-MCM7 lentivirus (n=3, three different donors). (b) The MCM7 level in NPCs treated with or without lentiviruses of circKIF18A overexpression and sh-MCM7 under oxidative stress (n=3, three different donors).

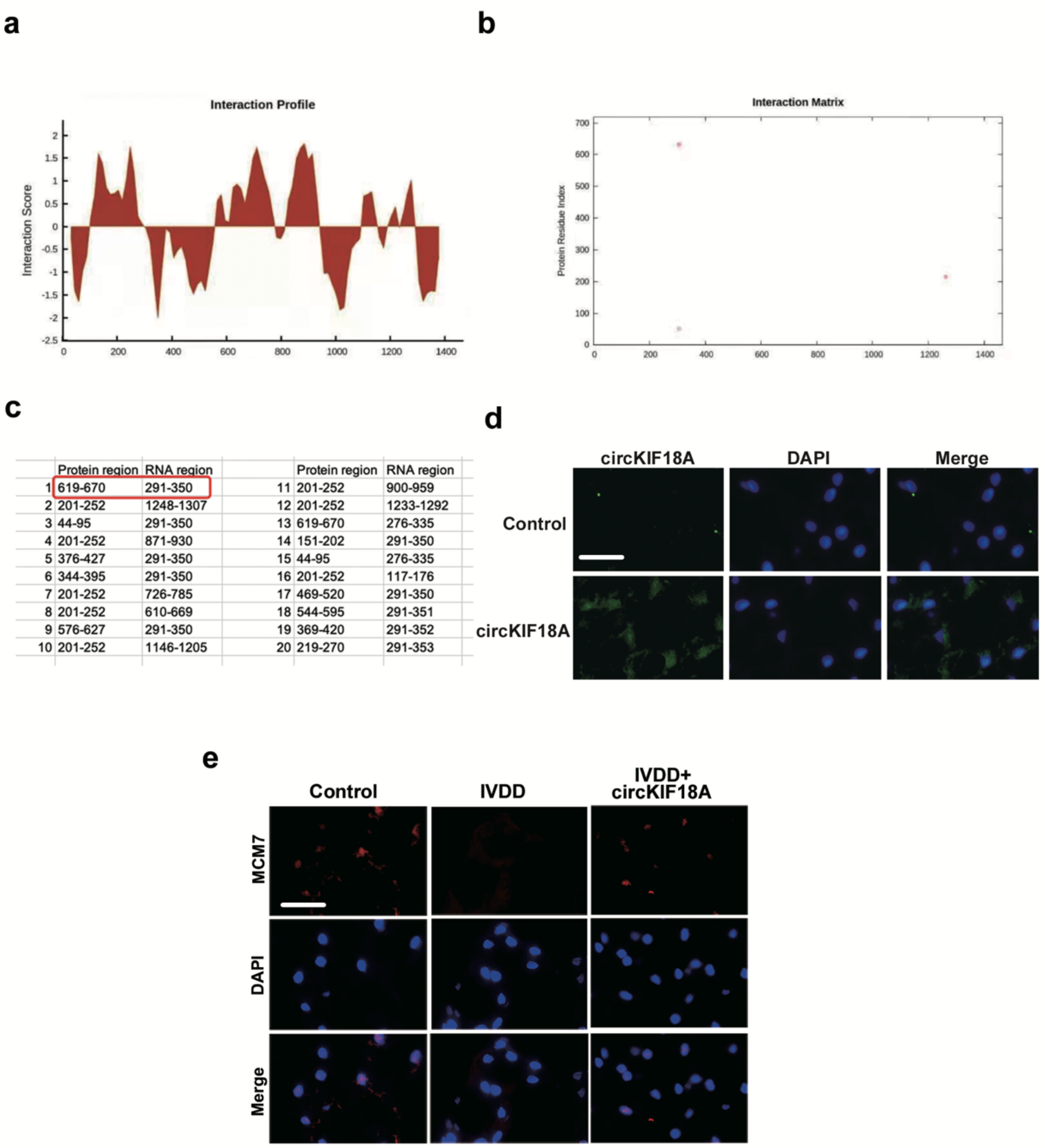


**Supplementary Fig 6. CircKIF18A regulates MCM7 in rat IVDD model.** (a) The predicted interaction information between human circKIF18A and rat MCM7 according to catRAPID database. The interaction profile between human circKIF18A and rat MCM7. (b) The interaction matrix between human circKIF18A and rat MCM7. (c) The predicted segments of interaction between human circKIF18A and rat MCM7. Red frame: the most potential binding segments of human circKIF18A and rat MCM7. (d) Representative images of FISH assay about circKIF18A in IVDs

injected with human circKIF18A adenovirus (n=3; Scale bar: 50 μm). (e) Representative images of immunofluorescence about MCM7 in IVDs treated as described above (n=5; Scale bar: 50 μm).

**Supplementary Table 5. Clinicopathologic features of patients involved in this study.**

| Pfirrmann Grades | Number | Age (years) | BMI (kg/m$^2$) |
|---|---|---|---|
| I | 2 | 45.5±27.6 | 24.52±0.4206 |
| II | 5 | 58.2±11.54 | 24.86±0.7969 |
| III | 12 | 45.56±14.66 | 24.56±2.037 |
| IV | 36 | 58.22±10.64 | 23.72±2.615 |
| V | 45 | 58.91±15.05 | 24.24±2.763 |

*L: Lumber vertebrae; S: Sacral vertebrae

**Supplementary Table 6. Sequences of primers, shRNAs and probes**

| Primers | |
|---|---|
| CircKIF18A forward primer | GCTGACCAAACTGCCGAACA |
| CircKIF18A reverse primer | CGACCACCCCTTTTTGGGTAT |
| KIF18A forward primer | TACCCAAAAAGGGGTGGTCG |
| KIF18A reverse primer | TCCTGCCAGGTCAATGAGTG |
| MCM7 forward primer | ACTCTCAGAAACCTACCTGGAAG |
| MCM7 reverse primer | CAGCTTTTCGTAGAAATCCTCCT |
| CDR1as forward primer | TACCCAGTCTTCCATCAACTGG |
| CDR1as Reverse primer | ACACAGGTGCCATCGGAAAC |
| Human ACAN forward primer | GGGACCTGCAAGGAGACAGAG |
| Human ACAN reverse primer | TCAATCTCACACAGGTCCCCTTC |

| | |
|---|---|
| Human COL2A1 forward primer | CCAGATGACCTTCCTACGCC |
| Human COL2A1 reverse primer | TTCAGGGCAGTGTACGTGAAC |
| Human MMP3 forward primer | CCTACAAGGAGGCAGGCAAG |
| Human MMP3 reverse primer | CCCGTCACCTCCAATCCAAG |
| Human MMP13 forward primer | TCGGCCACTCCTTAGGTCTT |
| Human MMP13 reverse primer | AAGTGGCTTTTGCCGGTGTA |
| Human ACTB forward primer | AGAGCTACGAGCTGCCTGAC |
| Human ACTB reverse primer | AGCACTGTGTTGGCGTACAG |
| hsa_circRNA_101645 forward primer | GCGCGACTGTCAAAACTACA |
| hsa_circRNA_101645 reverse primer | TGGAGATGTGTTCAGCTTCG |
| hsa_circRNA_104508 forward primer | TCCACACTTCATCCTCACCA |
| hsa_circRNA_104508 reverse primer | CTCCCATTTGCCTCTATCCA |
| hsa_circRNA_102116 forward primer | TGGGCACAAACAGTTCATGT |
| hsa_circRNA_102116 reverse primer | TGCGTTTGAATGATTTTCCA |
| hsa_circRNA_103838 forward primer | TGCACTCAGCTCTTCAGCAT |

| | |
|---|---|
| hsa_circRNA_103838 reverse primer | GATGTTATCAGCCCCTCCAA |
| hsa_circRNA_101557 forward primer | TCCATTCCCTAATCCTCTGC |
| hsa_circRNA_101557 reverse primer | GGGGAACTGTACCTCTAAACCA |
| hsa_circRNA_104630 forward primer | CTTTTGCACGAGAACATGGA |
| hsa_circRNA_104630 reverse primer | TAAAGCTCCTGCTGCACCTC |
| hsa_circRNA_101709 forward primer | GATATGGGCACTGTGGTCCT |
| hsa_circRNA_101709 reverse primer | GCCTGATTGGTCCATCAACT |
| hsa_circRNA_104019 forward primer | ACGTTTTGTTCCCTTTGCTG |
| hsa_circRNA_104019 reverse primer | AAAGTGCTTTGATCCCATCG |
| hsa_circRNA_101370 forward primer | CGCATGTCTCCTTTTCCTCT |
| hsa_circRNA_101370 reverse primer | CACCTGAACACCACGAGAAA |

| | |
|---|---|
| hsa_circRNA_105031 forward primer | GATGCACGGTGCTACACCTA |
| hsa_circRNA_105031 reverse primer | CATGGGAGGATTAGCTGGAA |
| hsa_circRNA_103486 forward primer | AATGCACATTGTGTTCTGCAA |
| hsa_circRNA_103486 reverse primer | ACAGCATTTCTCCCAAGCAC |
| hsa_circRNA_101558 forward primer | TCTTTTTCCGGCAGATTTTG |
| hsa_circRNA_101558 reverse primer | GGGGAACTGTACCTCTAAACCA |
| hsa_circRNA_100427 forward primer | TGAATTCAAATTCCGAAGCA |
| hsa_circRNA_100427 reverse primer | CCTTTCTCGTAGGGGGTCTC |
| hsa_circRNA_102618 forward primer | AACTCAGGCATGCTTCTGAC |
| hsa_circRNA_102618 reverse primer | GGGGTAGAGACAAACATCATTAAG |
| hsa_circRNA_103139 forward primer | CATCCCTACCCAACAGAGGA |

| | |
|---|---|
| hsa_circRNA_103139 reverse primer | GCCATCATGGTTCATCAGAG |
| hsa_circRNA_103198 forward primer | GCATGGAGGTCTATGCCAGT |
| hsa_circRNA_103198 reverse primer | TCAGCCACTCTTGACACTGC |
| hsa_circRNA_102492 forward primer | GACCGTGTGCTGCTGAAGTA |
| hsa_circRNA_102492 reverse primer | GATTCCTTTGGTCCAGGATG |
| hsa_circRNA_103634 forward primer | TCCTGAAACCACTCAAGAGTCA |
| hsa_circRNA_103634 reverse primer | GGCACACTGGTACAAAGCAG |
| hsa_circRNA_102126 forward primer | ATTCCATCCGTTGCATGACT |
| hsa_circRNA_102126 reverse primer | TTTCCTCCACTGGGCTACAG |
| | |
| shRNAs | |
| hsa_vector | UUUGUACUACACAAAAGUACUG |
| hsa_circ_0021535 shRNA #1 | GCCTATTCCTTGTTGTATATA |

| | |
|---|---|
| hsa_ circ_0021535 shRNA #2 | AGCCTATTCCTTGTTGTATAT |
| hsa_MCM7 shRNA #1 | GTGGAGAAAGAAGATGTGAA |
| hsa_MCM7 shRNA #2 | CCGAGTTGGTGGACTCAATT |
| hsa_UBE3A shRNA | GTCCCTGTATCTAACTCAAAT |
| | |
| RNA pulldown probe | |
| hsa_circ_0021535 probe | AAUCUGUUCAUUAUAUACAACAAGG |
| Oligo probe | UUUUCAGAGAUAAUCUAGUCAAAAC |
| | |
| FISH probe | |
| hsa_circ_0021535 probe | AAUCUGUUCAUUAUAUACAACAAGG |
